# TFG Promotes Organization of Transitional ER and Efficient Collagen Secretion

**DOI:** 10.1016/j.celrep.2016.04.062

**Published:** 2016-05-12

**Authors:** Janine McCaughey, Victoria J. Miller, Nicola L. Stevenson, Anna K. Brown, Annika Budnik, Kate J. Heesom, Dominic Alibhai, David J. Stephens

**Affiliations:** 1 Cell Biology Laboratories, School of Biochemistry, Faculty of Biomedical Sciences, University of Bristol, University Walk, Bristol BS8 1TD, UK; 2 Institut für Biophysik, Leibniz Universität Hannover, Herrenhäuserstraβe 2, 30419 Hannover, Germany; 3 Wolfson Bioimaging Facility, Faculty of Biomedical Sciences, University of Bristol, University Walk, Bristol BS8 1TD, UK; 4 Proteomics Facility, Faculty of Biomedical Sciences, University of Bristol, University Walk, Bristol BS8 1TD, UK

## Abstract

Collagen is the most abundant protein in the animal kingdom. It is of fundamental importance during development for cell differentiation and tissue morphogenesis as well as in pathological processes such as fibrosis and cancer cell migration. However, our understanding of the mechanisms of procollagen secretion remains limited. Here, we show that TFG organizes transitional ER (tER) and ER exit sites (ERESs) into larger structures. Depletion of TFG results in dispersion of tER elements that remain associated with individual ER-Golgi intermediate compartments (ERGICs) as largely functional ERESs. We show that TFG is not required for the transport and packaging of small soluble cargoes but is necessary for the export of procollagen from the ER. Our work therefore suggests a key relationship between the structure and function of ERESs and a central role for TFG in optimizing COPII assembly for procollagen export.

## Highlights

• TFG is required to organize transitional ER into larger structures
• Following depletion of TFG, ERESs remain in close apposition to the ERGIC
• Mini-ERESs support secretion of small soluble cargo
• Large ERESs are required for procollagen secretion

## Graphical Abstract

**Figure:**
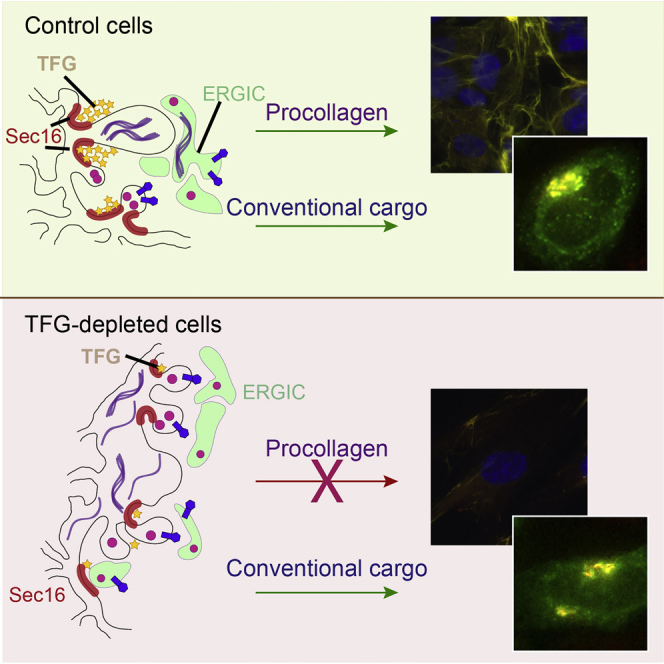

## Teaser

McCaughey et al. show that TFG is required to support the organization of ER exit sites (ERESs) into larger structures. This higher-order organization is required for efficient secretion of procollagen.

## Introduction

Collagen is the most abundant protein in the animal kingdom. Its secretion and assembly into a fibrillar extracellular matrix underpins core aspects of development such as directed cell migration, tissue morphogenesis, cell polarization, and bone formation. The fidelity of continued secretion and assembly of collagen throughout life is central to its role in key pathological states including fibrosis and cancer cell invasion. The earliest stages of procollagen assembly take place within the ER (Canty and Kadler, 2005). Fibril-forming collagens such as types I, II, III, V, XI, XXIV, and XXVII assemble as large trimeric structures within the ER that must then be packaged into vesicular carriers for transport to the Golgi and onward to the extracellular space (Mouw et al., 2014). This packaging of cargo and formation of vesicles at the ER membrane is mediated by the COPII complex.

COPII-dependent export of secretory cargo from the ER is essential for the normal function of all mammalian cells (Zanetti et al., 2011). COPII acts at specific sites on the ER membrane defined as transitional ER (tER). COPII drives the concentration of secretory cargo, deformation of the membrane, and subsequent formation of COPII-coated vesicles and tubules. COPII vesicles bud from the tER but remain localized within the immediate vicinity of it. These membrane carriers subsequently form fuse with (or fuse to form) the first post-ER compartment, the ER-Golgi intermediate compartment (ERGIC). These three structures, the tER, COPII-coated membranes, and the ERGIC, together form a unit known as an ER exit site (ERES). These cup-shaped structures (Bannykh et al., 1996) consist of a defined tER membrane with which Sec16 is closely associated (Hughes et al., 2009).

The process for the formation of COPII-coated vesicles is described classically as Sec12-dependent loading of GTP onto Sar1 followed by sequential recruitment of Sec23-Sec24 and Sec13-Sec31 and is sufficient for the generation of small 60- to 80- nm vesicles in vitro (Zanetti et al., 2011). Although these vesicles can accommodate the majority of cargo, additional flexibility is required, both physically and in terms of the completion of budding, to encapsulate larger cargo such as procollagen (Malhotra and Erlmann, 2011). Fibrillar collagen precursors (such as procollagen I at 300 nm; Bächinger et al., 1982) are too large to fit within classically described 60- to 80-nm vesicles, which has led to large amount of work to define the molecular requirements for COPII-dependent collagen secretion. From this, many factors have been identified that act either on COPII or immediately downstream of it that are required for collagen secretion. Mutations in the Sec23A subunit of COPII led to craniofacial development defects attributable to aberrant collagen secretion (Boyadjiev et al., 2006). Detailed mechanistic work has shown that the F382L mutation results in a loss of efficient coupling of Sec23-Sec24 to Sec13-31 (Bi et al., 2007, Fromme et al., 2007). Our own work showed a particular requirement for the outer layer Sec13-Sec31 in procollagen export in vitro and in vivo (Townley et al., 2008). The conclusion from this work is that highly efficient assembly of COPII is essential for normal secretion of procollagen (Saito and Katada, 2015).

Over time, many other factors have been described that act in concert with the core COPII machinery to enhance its efficiency or, in many cases, provide specialized mechanisms for the incorporation of particular cargo into COPII-coated carriers (Venditti et al., 2014). The majority of such accessory factors act either by directly modulating the COPII machinery through physical interaction or through engaging cargo directly. Many of these additional components of the ER export process have been shown to be involved in the efficient export of procollagen from the ER. For example, KLHL12 is a Cullin-3 adaptor that directs mono-ubiquitylation of Sec31A, which in turn is required for efficient formation procollagen-containing carriers (Jin et al., 2012). Further downstream of COPII lies the tethering machinery that includes the TRAPPI complex. A key subunit of this complex, Sedlin, is also required for the packaging of procollagen (Venditti et al., 2012), reinforcing the concept of efficient secretory pathway activity as a prerequisite for this process. Other proteins appear to be specific for subsets of procollagen isotypes, with TANGO1 in particular being required selectively for the packaging and secretion of procollagen VII (Saito et al., 2009). The selectivity of TANGO1 for procollagen VII is debated by some. A knockout mouse for TANGO1/Mia3 shows a diversity of defects that relate to defects in the deposition of multiple collagens including I, II, and VII. In addition, cTAGE5, shown previously to facilitate the role of TANGO1 in export of procollagen VII (Saito et al., 2011), has now been shown to act in localization of Sec12 (Saito et al., 2014) and therefore presumably as a more direct component of the canonical COPII budding machinery. Whether this is due to a direct role for TANGO1/Mia3 in packaging of multiple collagen isotypes or results from defects in the assembly of a complete extracellular matrix because of a selective loss of collagen VII remains unclear. In support of a dedicated pathway for procollagen VII export, Sly1 and Syntaxin-17 were also shown to be required (Nogueira et al., 2014). This also provides further evidence of the importance of the fusion machinery in the trafficking of procollagen VII through the early secretory pathway. This work also led to the suggestion that the ERGIC could provide additional membrane to facilitate the export of atypically large cargo from the ER through a process of maturation of the nascent carrier. In support of such models, Nakano and colleagues showed that a ‘hug-and-kiss’ mode of direct contact of the (unstacked) *cis*-Golgi with newly forming COPII-coated carriers in the yeast *S. cerevisiae* provides a mechanism for ER-to-Golgi transport (Kurokawa et al., 2014). This process also has interesting parallels in the role of ERES and ERGIC membranes in the formation of autophagosomes at the ER membrane (Ge and Schekman, 2014, Graef et al., 2013, Wang et al., 2014). In all these cases, the ERGIC is in some way providing membrane to the newly forming carrier.

Although many tethering and fusion factors have been defined that direct the consumption of COPII-derived vesicles, it is only recently that we have gained mechanistic insight into what could maintain the structure of the ERES itself. TFG was identified as a Sec16-interacting protein that modulates COPII-dependent budding from the ER (Witte et al., 2011). TFG appears to coordinate the spatial organization of the budding event and has been proposed to act by forming oligomeric assembly that acts as a meshwork to physically connect COPII-derived vesicles and the nascent ERGIC compartment (Johnson et al., 2015). Mutations in TFG cause sensory axon degeneration, hereditary spastic paraplegia, and Charcot-Marie-Tooth disease type 2 (Beetz et al., 2013, Lee et al., 2013, Tsai et al., 2014). These defects appear to be related to structural changes in the ER (Beetz et al., 2013) that could arise from defects in protein secretion.

Here, we show that TFG directs the organization of the very earliest stages of COPII-dependent budding, the assembly of the tER. Suppression of TFG expression results in small ERES that remain functional for the export of many secretory cargoes, but not for the trafficking of procollagen. TFG therefore provides a mechanistic link between the spatial organization of the ERES and the secretion of procollagen.

## Results

**Figure 1:**
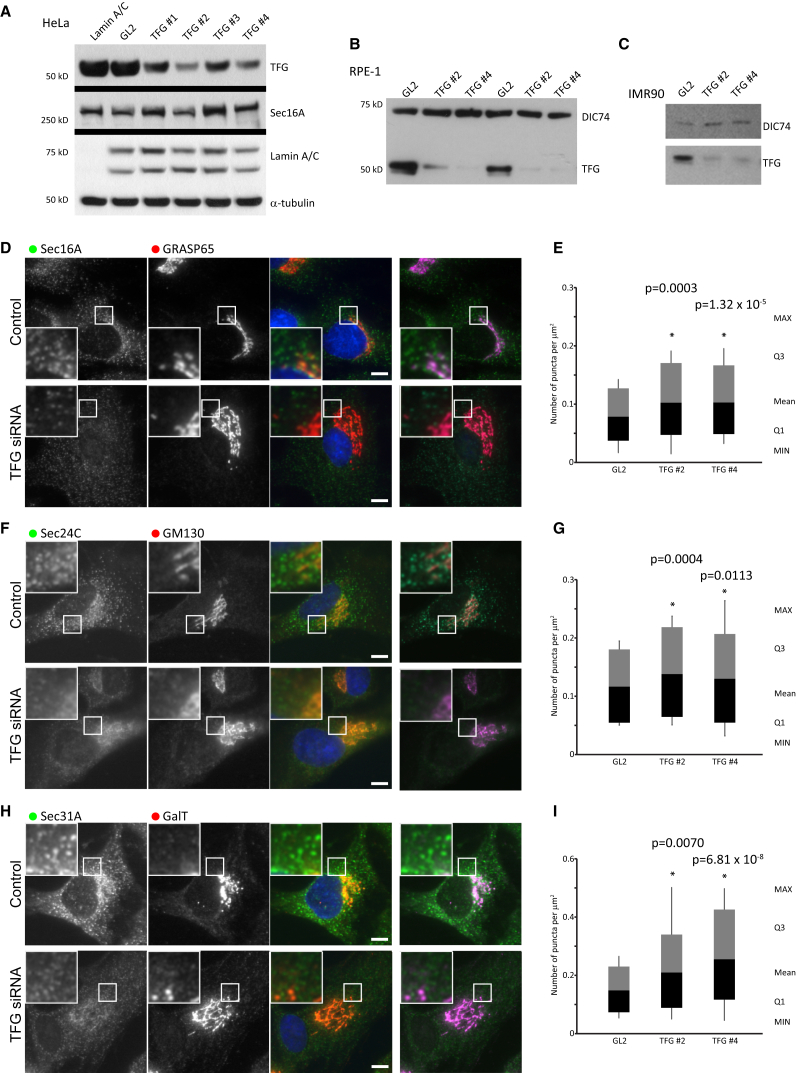
Validation of Depletion of TFG using siRNA (A) Immunoblotting of HeLa cell lysates from cells transfected with siRNAs targeting Lamin A/C or TFG or with a non-targeting control (GL2) as indicated. Four independent duplexes were used against TFG. Blots were probed to detect TFG, Sec16A, Lamin A/C, or α-tubulin as indicated. (B and C) Immunoblotting of (B) RPE-1 or (C) IMR-90 cell lysates from cells transfected with siRNAs targeting GL2 or TFG as indicated showing effective depletion of TFG compared to the dynein intermediate chain subunit DIC74. (D–I) Early secretory pathway organization in TFG-depleted cells. Widefield immunofluorescence microscopy of control or TFG-depleted cells labeled to detect (D) Sec16A and GRASP65, (F) Sec24C and GM130, and (H) Sec31A and GalT. RGB merges include DAPI labeling of nuclei. Green-magenta merges are also shown. (D), (F), and (H) show automated quantification of puncta numbers per μm^2^ for (E) Sec16A, (G) Sec24C, and (I) Sec31A.

To examine the role of TFG in the organization of mammalian ERESs and membrane traffic through the early secretory pathway, we used an RNAi approach. In line with recently published data, we found that effective depletion of TFG was possible without any overt signs of cell death. Longer-term depletion of TFG was found to cause extensive cell death. We have not been able to isolate a clone with a knockout of TFG using CRISPR (clustered regularly interspaced short palindromic repeats)-Cas9, which we suspect is due to lethality. Figure S1 shows the outcome of a transient knockout of TFG using this approach, but we have been unable to amplify cells showing this phenotype. Figure 1 demonstrates an effective knockdown of TFG expression in three human cell lines used in this study: HeLa (cervical carcinoma), RPE-1 (telomerase immortalized retinal pigment epithelial cells), and IMR-90 (fibroblasts) cells. Two of the four small interfering RNA (siRNA) duplexes used (TFG #2 and TFG #4) were consistently more effective, and these were used in all further studies. Analysis of scanned immunoblots using ImageJ showed a routine depletions were between 65% and 92% effective. Experiments showing a lower level of depletion were not included in the analysis.

Depletion of human TFG was originally shown to delay exit of tsO45-G-GFP from the ER (Witte et al., 2011). More recently, it was shown that depletion of TFG caused an increase in the number of COPII-positive structures in cells compared to tER sites (marked with Sec16A) (Johnson et al., 2015). Intriguingly, this did not appear to cause any obvious structural changes to the Golgi. Using conventional light microscopy, we observed that depletion of TFG caused not only an obvious reduction in the intensity of Sec16A labeling at tER sites but also an increase in the number of individual structures visible at this level of resolution (Figure 1D). This increase was statistically detectable using automated counting (Figure 1E). Similar data were seen for Sec24C (Figure 1F, quantified in Figure 1G) and Sec31A (Figure 1H, quantified in Figure 1I), classical markers of COPII-coated membranes. In all cases, COPII labeling was more diffuse and individual structures were less well defined, but automated quantification of the brightest 66% of objects showed the increase in number of structures for Sec16A, Sec24C, and Sec31A. This could arise either from an increase in ERES number from de novo biogenesis (Stephens, 2003) or from a disruption of the assembly of smaller ERESs into larger structures (as also described in Bevis et al., 2002).

**Figure 2:**
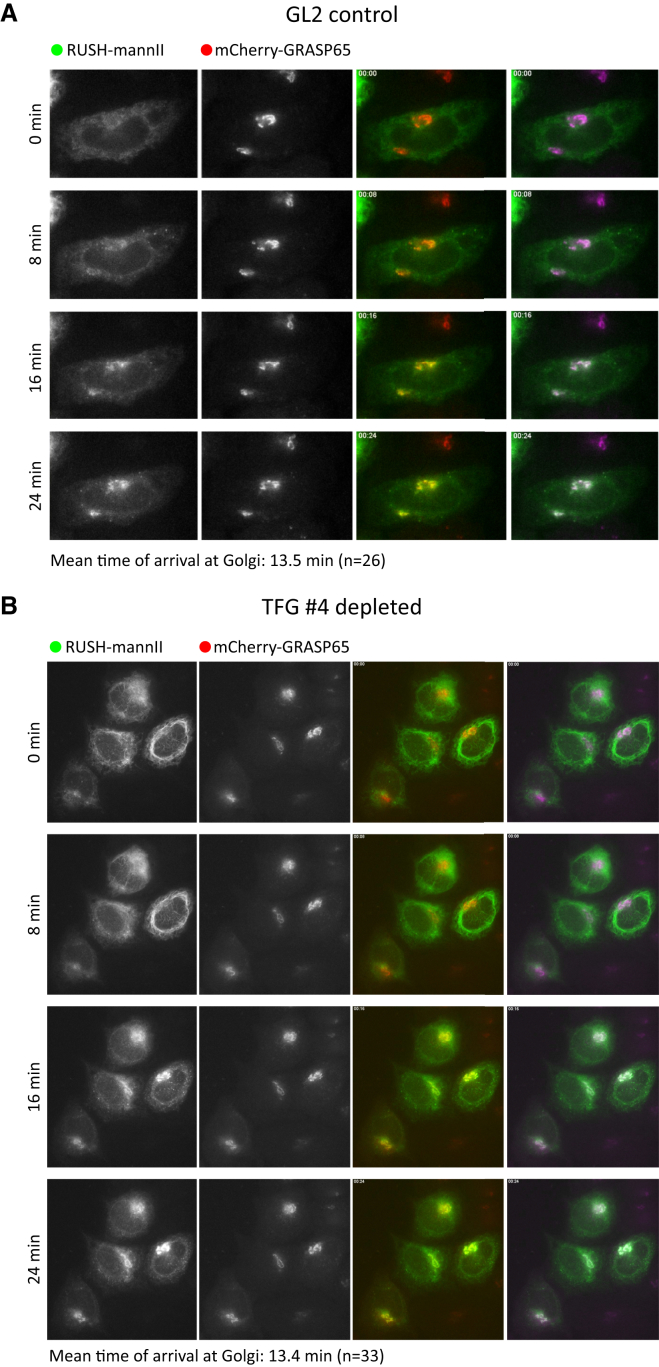
ER-to-Golgi Transport of Mannosidase II-GFP Is Unperturbed on TFG Depletion (A and B) Analysis of trafficking from ER-to-Golgi of GFP-tagged mannosidase II (mannII-GFP, green) using the RUSH system. Cells stably expressing GRASP65-mCherry (red) were transfected with (A) control (GL2) or (B) TFG siRNA duplexes and subsequently with constructs to retain mannII-GFP in the ER. Addition of biotin at time = 0 releases mannII-GFP from the ER. Images are shown from time-lapse movies that are included as supplemental material (Movies S1 and S2). RGB and green-magenta merges are shown.

In these datasets, we also noticed an obvious increase in the size of the Golgi when visualized using a variety of antibodies to classical markers of the Golgi (Figure 1D: Golgi reassembly and stacking protein of 65 kDa [GRASP65]; Figure 1F: Golgi matrix protein of 130 kDa [GM130]; and Figure 1H: galactosyltransferase T [GalT]). This was not manifest as a fragmentation but is more accurately described as a “loosening” of Golgi structure, reminiscent of that seen following depletion of the COPII coat component Sec13 (Townley et al., 2008). An enlarged Golgi is often associated with a moderate defect in anterograde protein trafficking from the ER to the Golgi. Indeed, depletion of TFG has been described to slow the export of cargo from the Golgi (Witte et al., 2011) and delay the reassembly of the Golgi following washout of brefeldin A (Johnson et al., 2015). Both of these assays require substantial perturbation to the cells (temperature shifts or pharmacological treatments). We examined anterograde traffic from the ER to Golgi using the RUSH (retention using selective hooks) system (Boncompain et al., 2012) (Figure 2). This system uses a streptavidin-binding peptide (SBP) fused to an ER-resident “hook” that selectively retains a GFP-tagged reporter (in our case mannosidase II) in the ER until biotin is added exogenously. We expressed a construct encoding an ER-localized hook (invariant chain SBP) and a truncated form of mannosidase II fused to EGFP (mannII-GFP) in cells depleted of TFG and measured ER-to-Golgi transport time-lapse microscopy. Arrival at the Golgi was measured by colocalization with GRASP65-mCherry, which was expressed stably in these cells (Cheng et al., 2010). We observed no detectable difference in the arrival of mannII-GFP at the Golgi in TFG-depleted cells (Figure 2B) versus controls (Figure 2A). Indistinguishable data were obtained for other secretory cargo reporters including a non-temperature-sensitive version of the VSV-G glycoprotein, E-cadherin, and GalT (not shown). The mean time of arrival of the peak of mannII-GFP at the Golgi was 14 min in both cases.

**Figure 3:**
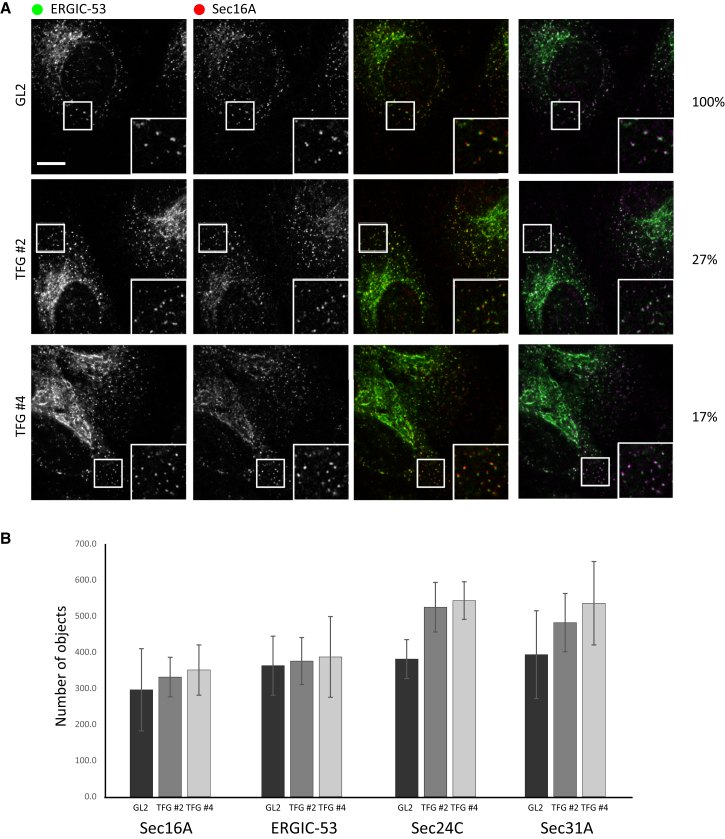
Super-resolution Imaging using STED of ERGIC-53 and Sec16A in Cells Depleted of TFG Cells depleted of TFG were immunolabeled as described and imaged using STED microscopy. (A) Images of cells include enlargements of boxed regions. Percentages indicate the approximate amount of TFG remaining in these experiments as monitored by immunoblotting. (B) Quantification of these data using MATLAB to define the number of individual objects including analysis of Sec24C and Sec31A as in (A).

In a recent paper, depletion of TFG was reported to increase the number of COPII-positive structures in cells without increasing the number of tER sites labeled with Sec16A (Johnson et al., 2015). A knock-on effect of this was shown to be a decrease in functional coupling of tER sites to the ERGIC as monitored by the close apposition (within 500 nm) of Sec16A and ERGIC-53. Our data from conventional light microscopy (Figure 1) suggested that we did indeed see an increase in COPII-positive structures (labeled for either Sec24C or Sec31A) but in our hands this was concomitant with an increase in tER (Sec16A). ERES are of the order of 500 nm in diameter, and therefore, to increase the resolution of our data beyond that of diffraction limited microscopy, we used gated stimulated emission depletion (gSTED) imaging to replicate this experiment. Using diffraction-limited fluorescent beads, we measured the full width at half maximum of our gSTED system as 90 nm (data not shown). We took tile scans of large areas of the coverslip to include multiple cells labeled for endogenous Sec16A and ERGIC-53 (Figure 3). We measured the numbers of Sec16A and ERGIC-53 positive structures and the distance between them using a custom-written MATLAB code (see Supplemental Information). In all experiments, we validated the efficiency of TFG suppression by immunoblotting (Figure S2).

Figure 3A shows the localization of Sec16A and ERGIC-53 in control cells as well as those depleted of TFG using gSTED microscopy. Visual inspection of the data in Figure 3A suggested no obvious difference between these conditions with a possible increase in number of objects labeled with each marker following depletion of TFG. Figure 3B shows the outcome of our automated image analysis of these data, showing the number of objects identified. These data do not reveal any increase in the number of Sec16A puncta or of ERGIC-53 puncta in cells depleted of TFG. We repeated these experiments labeling for Sec24C and Sec31A. Here (Figure 3B), we do define a statistically detectable increase in the number of Sec24C structures identified and a trend toward more individual structures (albeit not statistically detectable as an increase) for Sec31A. In our widefield data, we observed an apparent increase in the number of Sec16A and ERGIC-53-positive structures. These data differ from that in Figure 1 obtained using widefield microscopy. Our interpretation here is that by imaging using the higher-resolution gSTED method, we can now identify individual ERES substructures in greater detail. Thus, using gSTED, we can identify the same number of Sec16A/ERGIC-53-positive structures, regardless of whether these individual elements are clustered into larger structures (as we see in unperturbed cells) or more dispersed (as we see following TFG depletion).

**Figure 4:**
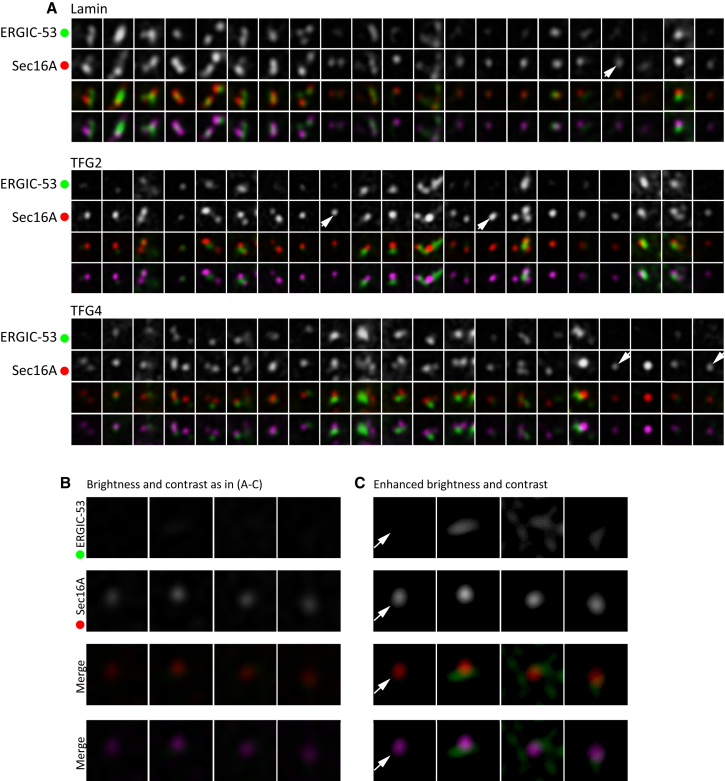
STED Imaging Shows Close Juxtaposition of Sec16A and ERGIC-53 Remains following TFG Depletion (A) Enlargements of individual structures from STED imaging as in Figure 4 following TFG depletion. Arrowheads indicate cases where Sec16A appears not to have an associated ERGC-53 puncta. (B and C) Stretching brightness and contrast reveals ERGIC-53 in close proximity to Sec16A following depletion of TFG in nearly all cases; one example of a “lone” Sec16A puncta is shown (arrow).

We then examined the proximity of tER to ERGIC as previous work suggested the two become decoupled on suppression of TFG (Johnson et al., 2015). Figure 4A shows enlargements of individual ERES from these cells; this illustrates the point that almost all Sec16A-positive structures have an ERGIC-53 positive structure in close apposition. In some rare cases (examples indicated by arrowheads in Figure 4A), it appears that Sec16A-positive structures exist in isolation. We then examined these more closely. Figure 4B shows that if one selects such objects from cells depleted of TFG (Figure 4B) and enhances the brightness and contrast of these images (Figure 4C), then in nearly all cases (three of four shown, arrowhead indicating a lone Sec16A puncta), one can then indeed define ERGIC-53-positive structures in close apposition.

**Figure 5:**
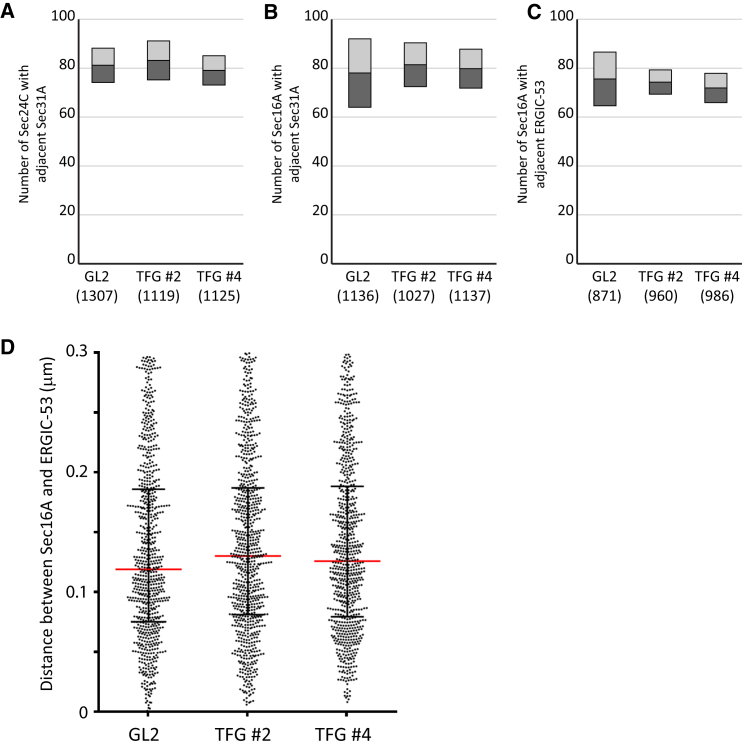
Automated Quantification of STED Data Show No Significant Change in Organization or Distribution of ERES Markers (A–C) Quantification of puncta within 300 nm of one another for (A) Sec24C and Sec31A, (B) Sec16A and Sec31A, or (C) Sec16A and ERGIC-53. (D) Measurements of the proximity of Sec16A and ERGIC-53 following depletion of TFG.

We repeated these experiments to measure the distribution of Sec24C relative to Sec31A and of Sec16A relative to Sec31A. Our analysis defines the centroid of each object according to a 2D Gaussian function. This allowed us to measure the distance between centroids to define whether individual labels are uncoupled from one another following TFG depletion. Automated counting of individual structures was used to define those structures within 300 nm of one another. These data (Figures 5A–5C) show no statistically detectable difference in the numbers of Sec16A puncta with adjacent Sec24C (Figure 5A), Sec31A (Figure 5B), or indeed ERGIC-53 (Figure 5C) puncta following depletion of TFG. From these data, we also determined that there was no detectable change in the distance between Sec16A and ERGIC-53-positive structures in cells (Figure 5D). Our data show that depletion of TFG causes a change in ERES organization. We then used our automated analysis to measure the distance between Sec16A-positive puncta (Figure S3A). Despite a trend toward an increased separation, this was not statistically detectable as a difference (Figure S3B). This is perhaps not surprising, as an increased distance away from an original structure would result in a decrease in distance to the next closest neighbor. We do however detect a significant decrease in the size of Sec16A-positive puncta (Figure S3C) consistent with our interpretation of a dispersion of tER elements following TFG depletion. It is also possible that very small structures are below the detection limit.

To define further the effect of TFG in secretory pathway function, we then examined the trafficking of extracellular matrix proteins. Considerable previous work has demonstrated a central role for COPII function in the secretion of procollagen. Given our inability to detect any defect in canonical ER-to-Golgi traffic following depletion of TFG using the RUSH system, we took a proteomic approach to give a broader picture of the role for TFG in the secretion of extracellular matrix proteins. We used stable isotope labeling of amino acids in culture (SILAC) to compare the cell-derived matrix secreted by control and TFG-depleted cells. We considered it possible that any defects we observed here could be due to disruption of Golgi architecture as an indirect consequence of TFG depletion. Therefore, we included cells depleted of the Golgi matrix protein giantin (using a previously validated siRNA targeting giantin, Gi#1; Asante et al., 2013). Giantin is required to maintain Golgi structure but does not affect ERES organization (not evident in Koreishi et al., 2013; see Figure S4) and therefore provides an additional control. We used RPE1 cells for these experiments to complement those described above. Table S1 lists those proteins that were found in either control or giantin-depleted cell-derived matrix but that were below the level of detection following depletion of TFG (removing any proteins where only one peptide was detected). These data identify a large number of proteins associated not only with the ECM itself but also with cell adhesions. Table S2 shows a list of those proteins that were decreased in abundance >3-fold more following TFG depletion than giantin-depletion. This list is rank-ordered according to the greatest difference in abundance between TFG- and giantin-depleted cells. In addition to several unexpected proteins, including the COPII subunit Sec13 (along with other nuclear pore components that might be related to the coupling of nuclear pore complexes to adhesion complexes; Mellad et al., 2011), we observe multiple collagen subunits including those of collagens I, IV, and VI. The identification of these particular collagen isotypes likely reflects their expression in RPE-1 cells.

**Figure 6:**
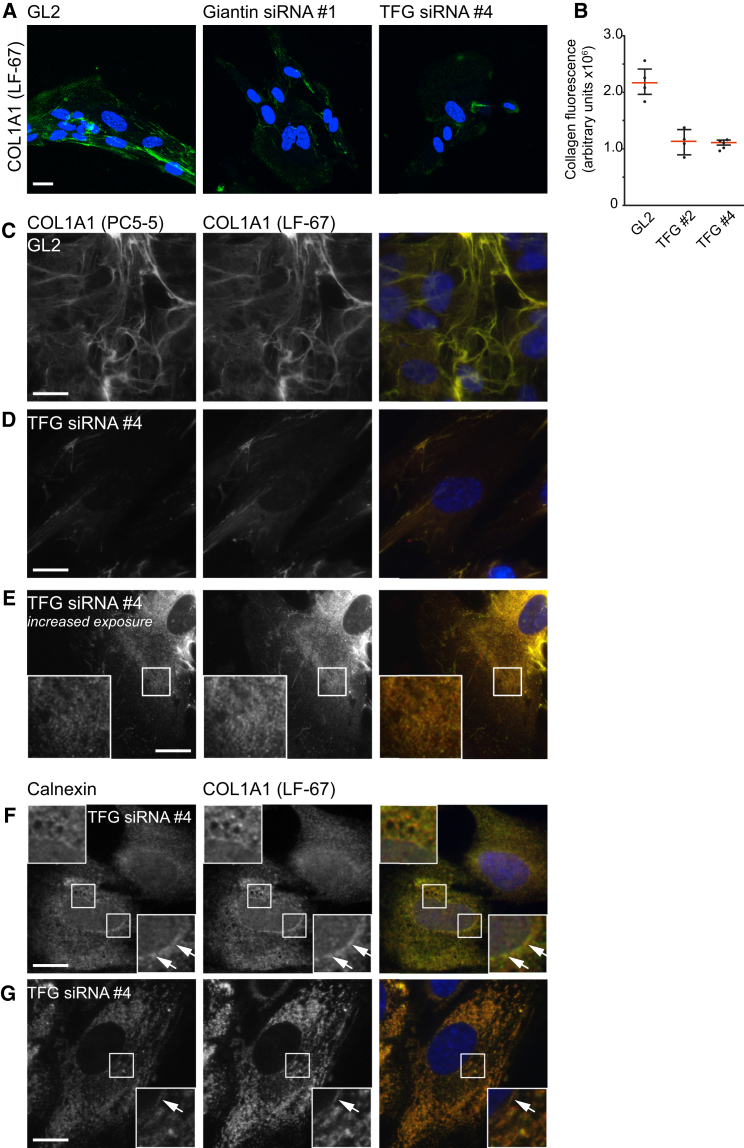
TFG Depletion Causes Defects in the Secretion of Procollagen Cells were fixed, but not permeabilized, and labeled with an antibody to detect the C-terminal telopeptide of collagen type IαI. (A) Images of cells showing extracellular collagen fibrils. (B) Quantification of the cellular fluorescence of extracellular procollagen. (C–G) Intracellular localization of procollagen in RPE1 cells. Cells were depleted using (C) control or (D–F) TFG siRNA duplexes, incubated for 6 hr in cycloheximide to prevent further protein synthesis, and subsequently with ascorbate for 90 min to promote procollagen folding and trafficking. Cells were fixed using paraformaldehyde, permeabilized and labeled to detect procollagen as indicated, and imaged by widefield microscopy. (C) Extracellular collagen fibrils are evident in controls as in (A). Following depletion of TFG (D), these fibrils are largely absent. Increasing the exposure time enables visualization of intracellular collagen. (E) Accumulation in puncta and reticular structures consistent with an ER localization. (F) Procollagen colocalizes with calnexin in TFG-depleted cells even in the presence of ascorbate. Two examples are shown following incubations with ascorbate for (F) 30 min and (G) 90 min. Scale bars represent 10 μm.

We then sought to validate these findings in IMR-90 human fibroblast cells, commonly used to study secretion of procollagen I. Using an antibody to the C-terminal telopeptide of collagen 1α1, we found that depletion of either giantin or TFG (Figure 6) leads to a defect in the assembly of extracellular collagen I-positive fibrils (Figure 6A). Image quantification (from experiments using two independent TFG siRNA duplexes; Figure 6B) demonstrates that this effect is significant. We included cells depleted of giantin in these experiments to further validate our proteomics and because there is clear evidence of a role of giantin in matrix assembly and collagen secretion (Katayama et al., 2011). Further analysis using widefield microscopy using an additional procollagen-specific antibody confirmed that depletion of TFG largely prevented the extracellular deposition of collagen fibrils (Figures 6C and 6D). Analysis of permeabilized cells with longer exposure times showed that procollagen accumulated inside the cells in a punctate and reticular distribution consistent with an ER localization (Figure 6E). Labeling of cells for both procollagen and the ER marker calnexin demonstrates that even after the addition of ascorbate, procollagen is retained in the ER in TFG-depleted cells (Figures 6F and 6G; arrowheads indicate nuclear membrane staining which is typical of ER). We do note that not all labeling coincides perfectly, suggesting that procollagen may accumulate in a subdomain of the ER in these cells. Thus, while depletion of TFG does not appear to affect the trafficking of small soluble cargoes, these data define a clear role in the secretion and/or assembly of procollagen.

## Discussion

**Figure 7:**
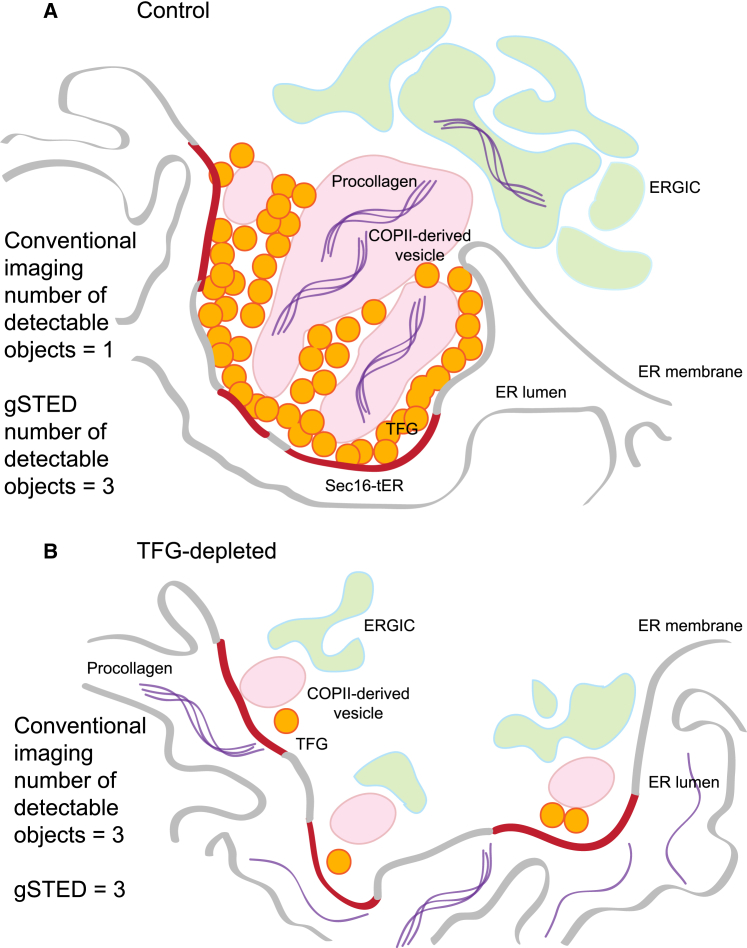
TFG-Dependent Higher-Order Organization of ERESs Supports Export of Procollagen from the ER (A) Normally, TFG organizes the ERES to cluster elements of tER together. These are not resolvable by conventional light microscopy but can be discriminated using super-resolution methods. This clustering supports the formation of procollagen-containing carriers in a COPII-dependent manner. These then mature to the ERGIC. (B) On depletion of TFG, individual tER elements become separated and detectable by conventional light microscopy and super-resolution imaging (gSTED). The proximity of ERGIC membranes to these smaller tER elements is unperturbed. Our model proposes that clustering of tER elements promotes efficient COPII assembly to support the formation of larger procollagen-containing carriers. It remains possible that tER clustering and procollagen export are not linked directly and that TFG acts in tER organization and in collagen secretion through separate mechanisms.

Our data suggest that TFG acts to tether or organize tER elements and identify a clear role for TFG in the secretion of type I procollagen from non-transformed human fibroblasts. We propose a model for this in Figure 7 in which TFG organizes Sec16-positive tER elements together to provide a larger, more effective platform for collagen secretion (Figure 7A). This is consistent with its identification as a Sec16-binding protein (Witte et al., 2011). The depletion of TFG results in a fragmentation of the minimal elements that make up an ERES (Figure 7B). Only when in this dispersed state are these individual elements resolvable by conventional light microscopy hence why this approach gives us an apparent increase in object number. In contrast, super-resolution imaging allows us to resolve these structures (for Sec16A and ERGIC-53) in both the unperturbed and TFG-depleted states, defining the change in organization rather than object number per se here. The increase in the number of Sec24 and Sec31-coated structures that we see is in close agreement with the work of Johnson et al. (2015) and suggests that TFG serves to tether these COPII-coated post-ER structures to the underlying tER. Many studies have demonstrated an increase in ERES number with increasing cargo load (Forster et al., 2006, Guo and Linstedt, 2006). The ability to change the organization of ERES units could also be relevant under increased cargo load as a means to provide a rapid increase in ERES number. Specifically, these studies examined the ER export of small, conventional cargo. Our analysis of controlled transport assays shows no change in the rate of delivery of small soluble cargo from the ER to the Golgi following TFG depletion. Adaptation by “untethering” ERES could be beneficial for such cargoes on a more rapid timescale than biogenesis of new ERES.

Our proteomic analysis of cell-derived matrix and immunofluorescence labeling of extracellular, processed collagen demonstrates a clear defect in matrix secretion, notably that of fibrillar collagens (collagen VI in RPE-1 and collagen I in IMR-90 cells) on depletion of TFG. As discussed above, many factors have been identified that are either required for or support the ER export of one or more procollagen isotypes (Malhotra and Erlmann, 2015). The link we define here between the organization and function of ERES in collagen secretion suggests that many of these factors might act in a similar manner by optimizing the organization and/or efficiency of these sites for the assembly of large COPII cages and the transition of these cages to the ERGIC. Defects in the Sedlin component of the TRAPPI complex (Venditti et al., 2012), TANGO1 (Saito et al., 2009), cTAGE5 (Saito et al., 2011), the KLHL12 ubiquitylation machinery (Jin et al., 2012), or Sec13-Sec31 (Townley et al., 2008) have all been reported to cause defects in collagen secretion. We note that any individual defect appears sufficient to inhibit procollagen export suggesting a concerted mode of action of all such factors. Loss of function of these proteins is not necessarily linked to changes in tER or ERES organization. However, changes in the recruitment of Sec12 by cTAGE5 (Saito et al., 2014) or of the Sar1 GTPase cycle by sedlin (Venditti et al., 2012) could be lined to changes in ERES organization, although this has not been tested systematically. Loss of function of giantin also results in apparent defects in collagen secretion (Katayama et al., 2011; our data here), but we have not defined a clear role for giantin in ERES organization (Figure S4). This reinforces the fact that while loss of TFG results in a defect in both tER organization and collagen secretion, this is not necessarily a causative relationship.

Many of these components should therefore be considered, along with TFG, as key factors in a functional minimal secretion system that must act together to optimize the system for the export of atypical cargo including fibrillar procollagens. In other cases as well, defects in secretory pathway machineries appear to cause primary defects in extracellular matrix deposition, chondrogenesis, and bone formation (Boyadjiev et al., 2006, Garbes et al., 2015, Katayama et al., 2011, Lang et al., 2006, Melville et al., 2011, Smits et al., 2010).

We currently have a very detailed understanding of the mechanisms of COPII-dependent budding from the ER, much of which has come from yeast genetics, in vitro reconstitution, and biochemical experiments (Zanetti et al., 2011). This has led to a detailed understanding of how COPII drives the accumulation of cargo into nascent buds and the fission of transport carriers from the ER. Newly emerging data have added to the complexity of this system to explain how it is adapted for more complex situations (Malhotra and Erlmann, 2015, Miller and Schekman, 2013, Saito and Katada, 2015). Models have been proposed that posit roles for post-ER membrane, notably the ERGIC, in facilitating this process for atypical membrane remodelling steps. It is widely accepted that COPII-coated vesicles uncoat and fuse either with each other (homotypically) or with a pre-existing ERGIC (heterotypically). In yeast, high-resolution light microscopy experiments indicated that mobile Golgi elements associate with COPII-coated carriers emerging from ERESs to mediate direct, efficient transfer of secretory cargo from the ER to the Golgi (Kurokawa et al., 2014). In mammalian cells, the ERGIC might play such a role, enabling the formation of larger pre-Golgi structures at peripheral sites.

An obvious means to achieve this with high efficiency would be to physically tether the tER from where COPII vesicles bud to the ERGIC. TFG has been proposed to mediate this tethering of COPII-coated membranes to the ERGIC. TFG assembles an oligomeric mesh that surrounds COPII vesicles (Johnson et al., 2015); it was suggested that this also tethers them to the ERGIC. This model was based in part on an apparent loss of connectivity between COPII-coated ERESs and ERGIC membranes following depletion of TFG. Our data do not support this model, since we do not see any change in the juxtaposition of tER and ERGIC on depletion of TFG. Our super-resolution imaging shows that nearly all Sec16-postive tER sites sit adjacent to an ERGIC, even following depletion of TFG. No increase in the number of ERGIC-53-positive structures is seen following depletion of TFG at this higher level of resolution, nor does the average distance between these structures change. Combined with our extensive validation of TFG depletion, we conclude that TFG does not tether the ERGIC to the rest of the ERES. It remains possible that an even greater level of depletion of TFG than we achieved might result in a loss of tER-ERGIC coupling. However, no physical linker has been defined that could link TFG to the ERGIC. Although our data do not preclude a role for the ERGIC in providing membrane to support budding of large carriers from the ER (Santos et al., 2015), our data suggest that TFG does not mediate this directly.

Recent data implicate the ERGIC as an active player in budding from the ER, notably in what one might consider atypical ER export events. Both autophagosome biogenesis (Ge et al., 2013, Ge and Schekman, 2014, Graef et al., 2013) and procollagen export (Nogueira et al., 2014) have been shown to involve ERGIC-derived membranes. In autophagosome biogenesis, this has been defined as the site of LC3 lipidation (Ge et al., 2013). During the export of procollagen from the ER, specific SNARE proteins Sly1 and Syntaxin-18 (Nogueira et al., 2014) mediate fusion of procollagen-containing carriers from the ER with ERGIC-derived membranes to enlarge the nascent transport carrier such that it can encapsulate the atypically large cargo (Santos et al., 2015). Importantly the recruitment of ERGIC membranes during this process was found to be mediated by TANGO1, providing a physical link between the cargo (procollagen VII), the COPII coat, and the ERGIC membranes required for this step. Our data are consistent with this model but show that physical coupling of ERGIC to tER alone is not sufficient to support procollagen export in the absence of large ERESs.

In summary, we favor a model (Figure 7) in which TFG serves to organize ERES membranes, specifically the tER and COPII-coated carriers, to support procollagen export. Our data support the concept that TFG acts as a scaffold to maintain larger clusters of Sec16A and retain COPII-coated elements in close proximity to one another. In its simplest form, this model would suggest that larger platforms of Sec16 are more competent for efficient COPII assembly at the earliest stages of budding to ensure encapsulation of a nascent procollagen-containing carrier within a COPII-coated structure. Alternatively (or in addition), TFG could act to scaffold carrier expansion during emergence of procollagen from the ER or support homotypic fusion of COPII-derived vesicles to expand nascent carriers. Our data show that TFG is not required to maintain the proximity of ERGIC membranes to tER sites, and therefore, we conclude that this juxtaposition of membranes is not sufficient to support procollagen secretion. Instead, our data are consistent with models in which the higher-order organization of these membranes into a larger ERES optimizes COPII-dependent budding to facilitate procollagen packaging at the ER.

## Experimental Procedures

### Widefield, Confocal, and Super-resolution Microscopy

Widefield microscopy on live cells was performed using Olympus IX81 microscope with 60× 1.42 numerical aperture (NA) oil-immersion lens, Sutter DG4 illumination with excitation filters, and multi-pass dichoric and multi-pass emission filters (Semrock). Images were collected using an Orca Flash 2.8 sCMOS controlled using Volocity 5.4.2 (PerkinElmer). Fixed cells were imaged using an Olympus IX70 microscope with 60× 1.42 NA oil-immersion lens, Exfo 120 metal halide illumination with excitation, dichroic and emission filters (Semrock), and a Photometrics Coolsnap HQ2 CCD, controlled by Volocity 5.4.1 (PerkinElmer). Chromatic shifts in images were registration corrected using TetraSpek fluorescent beads. Confocal images were obtained with Leica SP5II, and for super-resolution microscopy, a Leica Microsystems STED SPX was used. STED microscopy samples were mounted with Prolong Diamond, and Alexa-Fluor-532-and Alexa4-88-conjugated secondary antibodies were used (all from Thermo Fisher Scientific). For localization analysis, antibody combinations were imaged as two-color STED tile scans consisting of 42 tiles containing approximately ten cells in focus. The field of view was randomly picked, but if necessary, it was moved to an area with more cells in the same focal plane. For data analysis, an area of 9.65 μm^2^ in the cell periphery of ten cells per sample was selected to even out the number of puncta and background noise between analyzed fields. Laser intensities were optimized for GL2 samples for each replicate. Settings among GL2, T2, and T4 samples within one set were not changed. Tile scans were performed by using the sequential scanning mode between frames, where the 488-labeled proteins were imaged first. To ensure that chromatic shift has no influence on the data analysis, four color 40-nm beads (TetraSpek, Life Technologies) were imaged with the mentioned settings and the automatic chromatic shift calculator of Volocity determined a −1 shift in y (not detectable by eye), which was adjusted for in the MATLAB code used for the data analysis.

### Localization of ERES Components After TFG Depletion via Super-resolution Microscopy

With the help of a MATLAB code (see Data S1) the 9.65 μm^2^ fields of view of ten cells per sample were analyzed together and the distance between the centroids of puncta from channel 0 (Ch0) and channel 1 (Ch1) was measured and the number of puncta, as well as neighbors in the other channel counted. For a distance analysis a cut off of 300 nm was chosen to prevent measurement of distances from puncta too far away from each other. This range corresponds to the estimated maximum size of large COPII vesicles (∼400 nm), as the diameter of measured puncta is ∼90–100 nm. Also, if a distance above 300 nm is allowed, measurements showed distances between groups of puncta, that already have a corresponding partner in the other channel.

### MATLAB Code for Analysis of STED Data

Images were automatically analyzed with custom-written code (MATLAB 2014a, MathWorks). Images were first corrected for any chromatic aberrations or shifts as identified through imaging of multi-color 100-μm fluorospheres (TetraSpek, Life Technologies). For each channel, images were pre-processed using a 2D wiener with a kernel radius of 2 (denoising) and then converted to frequency space through use of a 2D Fourier transform. Data were thresholded in the frequency domain (smoothed), and after applying an inverse Fourier transform, an adaptive local intensity threshold was applied to convert the image to binary. Touching objects were then separated through use of a watershed algorithm and objects parameters were quantified (area, centroid, and intensity parameters). A k-nearest-neighbor search was performed to look for the nearest 20 neighbors within 500 nm of each ERGIC-53. A further k-nearest-neighbor search was then performed within this search area to measure the distances between Sec16A proteins. All measured distances were then outputted to a spreadsheet for further statistical analysis.

### Statistical Analysis

Statistical analysis was done to estimate differences in protein localization between control and TFG-depleted samples. As the measured distances between centroids are non-parametrically distributed, statistical analysis was performed using Kruskal-Wallis test followed by Dunnett’s multiple comparison test using Prism 4.03 on the grouped values of all at least three independent replicates (n = 3).

## Author Contributions

D.J.S. conceived the project, performed experiments and data analysis, and wrote the paper. J.M. performed TFG depletion immunoblots and with D.A. performed the STED microscopy and data analysis. N.L.S. performed experiments and analyzed data relating to ERES distribution. V.J.M., J.M., and D.J.S. performed and analyzed collagen secretion experiments. A.B. and A.K.B. performed initial validation of TFG depletion and experiments in Figure 1. V.J.M. performed the CRISPR knockout experiments and all RUSH and proteomics experiments. K.J.H. helped design and perform the proteomic experiments and initial data analysis.

## Supplemental Information

Document S1. Supplemental Experimental Procedures, Figures S1–S4, Tables S1 and S2, and Supplemental Note

Movie S1. ER-to-Golgi Transport of Mannosidase II-GFP in GL2 siRNA-Transfected Cells, Related to Figure 3

Movie S2. ER-to-Golgi Transport of Mannosidase II-GFP in TFG-Depleted Cells, Related to Figure 3

Data S1. Analysis of STED Data, Related to Figures 4, 5, and S3 The Matlab code used to analyze data in Figures 4, 5, and S3 is included in this file. A description of how to implement this code is included as a Supplemental Note.

Document S2. Article plus Supplemental Information
