# Supplementary material for: TFG Promotes Organization of Transitional ER and Efficient Collagen Secretion
Source: Cell Rep. 2016 May 12;15(8):1648–59. doi: 10.1016/j.celrep.2016.04.062 (PMC4885023; doi:10.1016/j.celrep.2016.04.062)
Supplement: Document S1. Supplemental Experimental Procedures, Figures S1–S4, Tables S1 and S2, and Supplemental Note [file mmc1.pdf]

Cell Reports, Volume *15*

Supplemental Information

TFG Promotes Organization of Transitional ER and Efficient Collagen Secretion

Janine McCaughey, Victoria J. Miller, Nicola L. Stevenson, Anna K. Brown, Annika Budnik, Kate J. Heesom, Dominic Alibhai, and David J. Stephens

List of supplementary materials

Supplemental Experimental Procedures.

Supplementary Figure S1: Transfection of RPE1 cells with Cas9 and guide RNA to knockout TFG expression. Relates to the rationale for Figure 1.

Supplementary Figure S2: Efficacy of depletion of TFG was monitored for each STED experiment to ensure representative image data. Relates to Figure 4 and 5.

Supplementary Figure S3: Additional data in response to reviewers. Relates to multiple figures, notably 4, 5, and 6.

Supplementary Figure S4: Additional data in response to reviewers. Relates to Figures 6 and 8.

Supplementary Movie 1: Relates to Figure 3. Movie for Fig 3A.avi
ER-to-Golgi transport of mannosidase II-GFP in control (GL2 siRNA-transfected) cells.

Supplementary Movie 2: Relates to Figure 3. Movie for Fig 3B.avi
ER-to-Golgi transport of mannosidase II-GFP in TFG-depleted cells.

Supplemental Table 1: Relates to text and rationale for experiments in Figure 7.

Supplemental Table 2: Relates to text and rationale for experiments in Figure 7.

Supplemental note: Matlab code for STED image analysis. This provides an explanation of implementation of the MatLab code used in Figure 4, 5, and S3.

Analysis_of_STED_data_2015_10_29.m: The code used to analyse data in figures 4, 5, and S3 is included in this file.

Supplemental Experimental Procedures

All reagents were purchased from Sigma-Aldrich (Poole, UK) or Thermo Fisher (Paisley, UK) unless stated otherwise.

Cell culture

The hTERT-RPE-1 (human retinal pigment epithelial cells immortalised with human telomerase reverse transcriptase, (Clontech)) cells were grown in DMEM F12 supplemented with 10 % heat-inactivated FCS (Thermo Fisher, UK). HeLa cells (ATCC CCL-2) were cultured in DMEM supplemented with 10 % heat-inactivated FCS. IMR-90 cells (ATCC CCL-186) were grown in minimal essential media supplemented with 5 ml l-glutamine and 10 % heat-inactivated FCS.

Depletion in RPE-1 and HeLa cells

TFG depletion via siRNA was performed using calcium phosphate buffer. On the first day 300 µL of cell suspension were seeded into a 6-well plate and incubated overnight. On the second day cells with a confluence of about 60 – 80 % were transfected with GL2, T2 or T4 siRNA. For the transfection buffer all stated components (using either T2 or T4 anti-TFG siRNA or GL2 anti-luciferase for control samples) were mixed and incubated at RT for 20 min to enable proper complex formation. Subsequently 200 µL of transfection buffer were added drop wise with a considerate distance onto the cells while rocking the plate, to provide finer distribution of the formed granulates. Transfected cells were incubated at 37 °C and 3 % CO_2 to help formation and dispersion of the calcium phosphate precipitate (Chen and Okayama, 1987). After 15 - 16 h the media was changed to remove excessive siRNA and transfection buffer, preventing toxic effects and the cultivation was presumed at normal conditions stated above. Fixation via ice cold methanol took place after an incubation of about 72 h after transfection at a confluence of about 70 %. If cells reached confluence within this period of time, they were passaged. siRNA duplexes were synthesised by MWG Eurofins or Sigma-Aldrich). Sequences used were GL2 (targeting luciferase) 5'-CGUACGCGGAAUACUUCGAUUTT-3'; TFG #2 5'-CUUCUCAGCCUACUAAUUA-3'; TFG #4 5'-ACUUCUGAGUAAUGAUGAATT-3'. Duplexes targeting giantin are described in (Asante et al., 2013).

For localisation of ERES components a total of 8 independent transfection experiments were performed and the level of TFG depletion was determined by semi-quantitative analysis via WB and subsequent ECL. Only depletions at or above 65 % relative to control samples GL2 were considered as sufficient, labelled for proteins of interest and imaged using super resolution microscopy (STED).

Depletion in IMR-90 cells and induction of collagen secretion

For TFG depletion in IMR-90 1 ml of cell suspension was seeded in a 6 cm dish with 5 ml of media and incubated for 2 days until a confluence of about 60 % was reached. Subsequent transfection was performed using calcium phosphate buffer as mentioned before with 600 µL buffer containing 5 µL GL2, T2 or T4 siRNA. Further treatment was conducted as described previously, with the exception that cells where split 24 h before fixation into 6-well plates with small cover slips using 950 µL cell suspension in 2 ml media containing 50 $\mu g \cdot ml^{-1}$ L-ascorbate-2-phosphate to trigger collagen secretion. Therefore, the labelled collagen corresponds to 24 h after induction of collagen secretion and a total of 48 h of TFG depletion. To visualise extracellular collagen type I, TFG depleted IMR-90 were fixed with PFA without subsequent permeabilisation and stained with LF-67. Images of cells labelled with LF-67 were quantified as corrected cellular fluorescence following background subtraction.

CRISPR-Cas9

Guide RNAs used were designed using the online tool from the Broad institute. hCas9 was a gift from George Church (Addgene plasmid # 41815, (Mali et al., 2013)). The gRNAs used were taken from the work of the Church lab described in (Mali et al., 2013) and found here: http://arep.med.harvard.edu/human_crispr/. Sequences were generated by gene synthesis downstream of a U6 promoter by MWG Eurofins. Targets were on chromosome 3 as follows: chr3:100447602-100447624 (minus strand) GAAGTTCTATCAGTTCTCGACGG and chr3:100455431-100455453 (plus strand) GCTCCTGCAGAAGATCGTTCAGG.

Analysis of efficacy of depletion

Semi-quantification of TFG levels was performed using the remaining cells in the 6-wells plates after removal of 13 mm cover slips used for immunofluorescence. Cells were washed with ice cold PBS and 200 µL of lysis buffer containing protease-inhibitor cocktail against serine and cysteine proteases was added per well and plates were incubated for 15 min on ice. Cells were scraped off the dish surface and cell lysate was centrifuged at 13000 rpm and 4 °C for 10 min. The cell pellet was discarded and the supernatant was stored at -20 °C until further analysis. A BCA protein assay was performed and measured using a BioPhotometer at a wavelength of 562 nm and total protein concentrations were calculated using a calibration curve.

Immunofluorescence of ERES and Golgi

For methanol fixation, media was aspirated, cells were washed with PBS and cold (-20°C) methanol was added. The cells were fixed for 4 min at -20 °C, following repeated rinsing with PBS. For paraformaldehyde fixation, cells were washed in PBS and fixed for 20 minutes in 4% paraformaldehyde in PBS. Cells were then washed for 5 minutes in PBS containing 30 mM glycine and permeabilized using PBS containing 0.1% Triton X-100. Storage of fixed cells till further processing was performed in blocking media (3 % BSA in PBS) overnight or for 0.5 – 1 h at RT. Samples were then rinsed three times for 5 min with PBS and cells were incubated upside down in primary antibody dilutions on parafilm, to ensure even labelling, for 0.5 – 1 h at RT. Rinsing steps with PBS were repeated and incubation with secondary antibodies against rabbit, conjugated with Alexa532 or 568, or mouse, conjugated with Alexa488, (1:400 diluted in PBS-BSA) was performed as described for primary antibodies following wash with PBS.

Samples were mounted using ProLong Gold Antifade with DAPI (Thermo Fisher) or ProLong Diamond (for super resolution microscopy) mounting media and stored overnight in the dark at RT to dry. Finally, slides were stored at 4 °C in the dark till microscopic analysis.

Immunofluorescence of ERES and Golgi

For immunofluorescence, cells were grown on 0.17 mm thick cover slips (sterilised using 70 % ethanol) in 6-well plates, fixed with ice-cold methanol for 4 minutes except for procollagen detection where 4% paraformaldehyde in PBS was used and immunostained using antibodies as indicated. Antibodies used: ERGIC-53 (monoclonal clone G1/93, Alexis Biochemicals), procollagen type I (LF67, recognizing the human collagen α1 (I) carboxy-telopeptide, Larry Fisher, NIH (Bernstein et al., 1995)), procollagen type I C-peptide (clone PC5-5, QED Bioscience, San Diego, CA), giantin (polyclonal, Cambridge Bioscience, Cambridge, UK), Sec16A (KIAA0310, Bethyl Labs, Montgomery, TX), Sec24C (Stephens Lab (Townley et al., 2008)), Sec24D (Stephens Lab (Palmer et al., 2005)), Sec31A (rabbit polyclonal, Stephens Lab (Townley et al., 2008) and mouse monoclonal, Transduction Labs), calnexin

(mouse monoclonal clone (BD Transductions Labs clone 37/Calnexin)), and TFG (Novus Biologicals, Cambridge, UK).

Imaging parameters for STED microscopy

Pinhole: 1 AU; unidirectional scanning. Tile size was 1976 x 1976 pixels, pixel size of 19.62 nm, scan speed 400 Hz. For 488 nm imaging: laser power set to 10%, hybrid detector set to 457-492nm, gating 0.3-6 ns, 100.5% gain, 1x line accumulation, 2 fold frame averaging, 592nm depletion laser set to 45% power. For 532 nm imaging, laser power was 15%, hybrid detector set to 544-590 nm, gating to 1.8 – 6 ns, gain to 120%, 1x line accumulation, 2 fold frame averaging. The depletion laser (660 nm) was set to 60%.

Controlled trafficking assays using RUSH

The Retention Using Selective Hooks system (Boncompain et al., 2012) was kindly provided by Franck Perez and Gaelle Boncompain (Institut Curie, Paris, FR) and is available through Addgene. We used a plasmid (Addgene #65255) encoding invariant chain fused to SBP and the minimal targeting region of mannosaidase II fused to GFP (mannII-GFP). Both proteins are expressed from the same plasmid using an internal ribosomal entry site. Other plasmids used included the following GFP-tagged reporters VSV-glycoprotein (Addgene #65300), E-cadherin (Addgene #65288), and GalT (Addgene #65272) each within RUSH system vectors. HeLa cells stably expressing GRASP65-mCherry ((Cheng et al., 2010) a kind gift from Jon Lane, University of Bristol) were depleted with siRNA for 48 hours followed by transfection with the relevant RUSH plasmids. After a further 24 hours, cells were washed in pre-warmed imaging medium (DMEM without phenol red and supplemented with 30 nM HEPES, pH 7.4) and transferred to a live cell imaging microscope at 37°C. After equilibration for 10 mins cells were imaged by time-lapse microscopy (1 frame per minute to minimize photodamage) following the addition of biotin (40 µm final concentration).

Proteomics of cell-derived matrix

Cells were grown in SILAC medium for 6 cell division cycles prior to siRNA transfection. Amino-acids stock R0, R6 and R10 were prepared at 8.4 $mg.ml^{-1}$ and K0, K6 and K8 at 14.6 $mg.ml^{-1}$. To make amino-acid stocks, 6 ml of sterile PBS for arginine or 3.5ml for lysine was added directly to the vial then filter sterilised using a 0.22 µm filter. For HeLa cells we used DMEM high glucose medium (Thermo Fisher) containing 5ml dialyzed FCS to which was added for "Light" 500 µl R0 + 500 µl K0 for "Medium" 500 µl R6 + 500 µl K4, and for "Heavy" 500 µl R10 + 500 µl K8. After siRNA transfection cells were grown for a further 72 hours. Cell derived matrix was prepared from these cells as follows. Cells were washed in PBS and extracted using pre-warmed extraction buffer (20 mM NH_4OH, 0.5% Triton X-100 in PBS; 3 ml per 6 cm plate for 2 minutes). Extraction buffer was aspirated and plates washed with PBS containing calcium and magnesium twice. Residual DNA was digested with 10 ug/ml DNase I (Roche) in PBS containing calcium and magnesium for 30 min at 37°C. Extracts were washed again twice with PBS containing calcium and magnesium and finally solubilized in 80 µl of SDS-PAGE sample buffer, resolved on precast PAGE gels (NuPAGE 4–12%; Invitrogen) and analyzed by liquid chromatography–MS/MS-based quantification as described in (Steinberg et al., 2012). Briefly, gel lanes were cut into 6 slices, each of which was digested in-gel with trypsin using an automated digestion unit (ProGest; Digilab UK) and the resulting peptides. Peptides were then fractionated using a nano-HPLC system (UltiMate 3000; DionexThermo Scientific) in 1% (vol/vol) formic acid. Peptides in 1% (vol/vol) formic acid were injected onto a C18 nanotrap column (Acclaim PepMap; Dionex, Thermo Scientific). After washing with 0.5% (vol/vol) acetonitrile, 0.1% (vol/vol) formic acid peptides were resolved on a 250 mm × 75–µm C18 reverse-phase analytical column

(Acclaim PepMap) over a 120-min organic gradient with a flow rate of 300 nl/min−1. Peptides were ionized by nano-electrospray ionization at 2.3 1 kV using a stainless steel emitter with an internal diameter of 30 μm (ES542; ProxeonThermo Scientific). Tandem MS analysis was performed on a mass spectrometer (LTQ Orbitrap Velos; Thermo Fisher Scientific). The Orbitrap was set to analyze the survey scans at 60,000 resolution, and the top six multiply charged ions in each duty cycle were selected for MS/MS in the LTQ linear ion trap. Data were acquired using the Xcalibar Xcalibur v2.1 software (Thermo Fisher Scientific). The raw data files were processed and quantified using Proteome Discoverer software v1.2 (Thermo Fisher Scientific) with searches performed against the UniProt human database by using the SEQUEST algorithm with the following criteria: Peptide precursor mass tolerance at 10ppm and MS/MS tolerance at 0.8Da peptide tolerance at 10 ppm, trypsin as the enzyme, and carboxyamidomethylation of cysteine as a fixed modification and oxidation of methionine and appropriate SILAC labels as variable modifications. The reverse database search option was enabled, and all data were filtered to satisfy a false discovery rate of <5%.

Supplemental References:

Bernstein, E.F., Fisher, L.W., Li, K., LeBaron, R.G., Tan, E.M., and Uitto, J. (1995). Differential expression of the versican and decorin genes in photoaged and sun-protected skin. Comparison by immunohistochemical and northern analyses. Lab. Invest. *72*, 662–669.

Mali, P., Yang, L., Esvelt, K.M., Aach, J., Guell, M., DiCarlo, J.E., Norville, J.E., and Church, G.M. (2013). RNA-guided human genome engineering via Cas9. Science *339*, 823–826.

Palmer, K.J., Konkel, J.E., and Stephens, D.J. (2005). PCTAIRE protein kinases interact directly with the COPII complex and modulate secretory cargo transport. J. Cell Sci. *118*, 3839–3847.

Steinberg, F., Heesom, K.J., Bass, M.D., and Cullen, P.J. (2012). SNX17 protects integrins from degradation by sorting between lysosomal and recycling pathways. J. Cell Biol. *197*, 219–230.

Legends to Supplemental Figures

Supplementary Figure S1: Transfection of RPE1 cells with Cas9 and guide RNA to knockout TFG expression. Cells were transfected with Cas9 and CRISPR guides and 72 hours later immunolabelled for giantin to identify the Golgi and TFG. The asterisk indicates an example of a cell with apparent loss of TFG expression (or at least depletion possible through one allele being hit) and concomitant disorganization of the Golgi as one might expect from a defect in ER export. Labelling of TFG is diffuse and likely non-specific in cells in which there is a sever disruption to Golgi structure. Despite repeated attempts, we were never able to clone out these cells.

Supplementary Figure S2: Efficacy of depletion of TFG was monitored for each STED experiment to ensure representative image data. The "loosening" of the Golgi was also used as a proxy for efficacy.

Supplementary Figure S3: (A) Images from the STED data showing the variation in distances between Sec16A puncta. This variability confounds the analysis of proximity. While the distance to the original nearest neighbour is increased, that object will lie closer to another (different) Sec16A-positive puncta. (B) The distance between Sec16A puncta is not statistically detectable as a significant difference. While there is a trend towards more separated objects, our automated quantification does not detect a significant difference on TFG depletion. (C) Analysis of the size of Sec16A-positive puncta shows a statistically detectable decrease in puncta size following depletion of TFG. The analysis is complicated here by the fact that brighter objects would appear larger. Data are pooled from 3 independent experiments, >600 puncta measured in each case. Asterisks indicate statistical significance.

Supplementary Figure S4: (A, B) Widefield images of control cells and cells depleted of giantin by (A) siRNA or (B) shRNA and labelled for giantin and Sec31A as indicated. Equivalent images were acquired with the same settings. (C, D) Quantification of >100 cells in each case shows no statistically detectable difference between control and giantin-depleted cells in terms of the number of individual puncta for (C) Sec24C or (D) Sec31A. Bars = 10 µm.

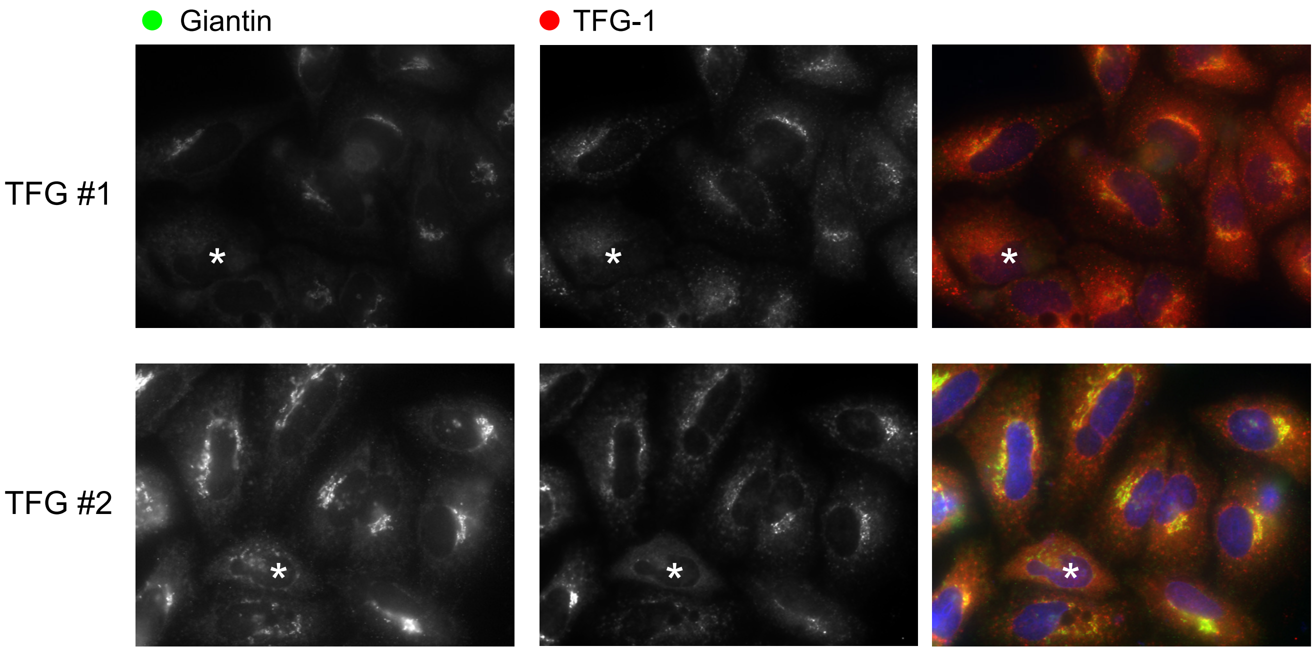
Giantin
TFG-1
TFG #1
TFG #2
*
*
*
*
*
*

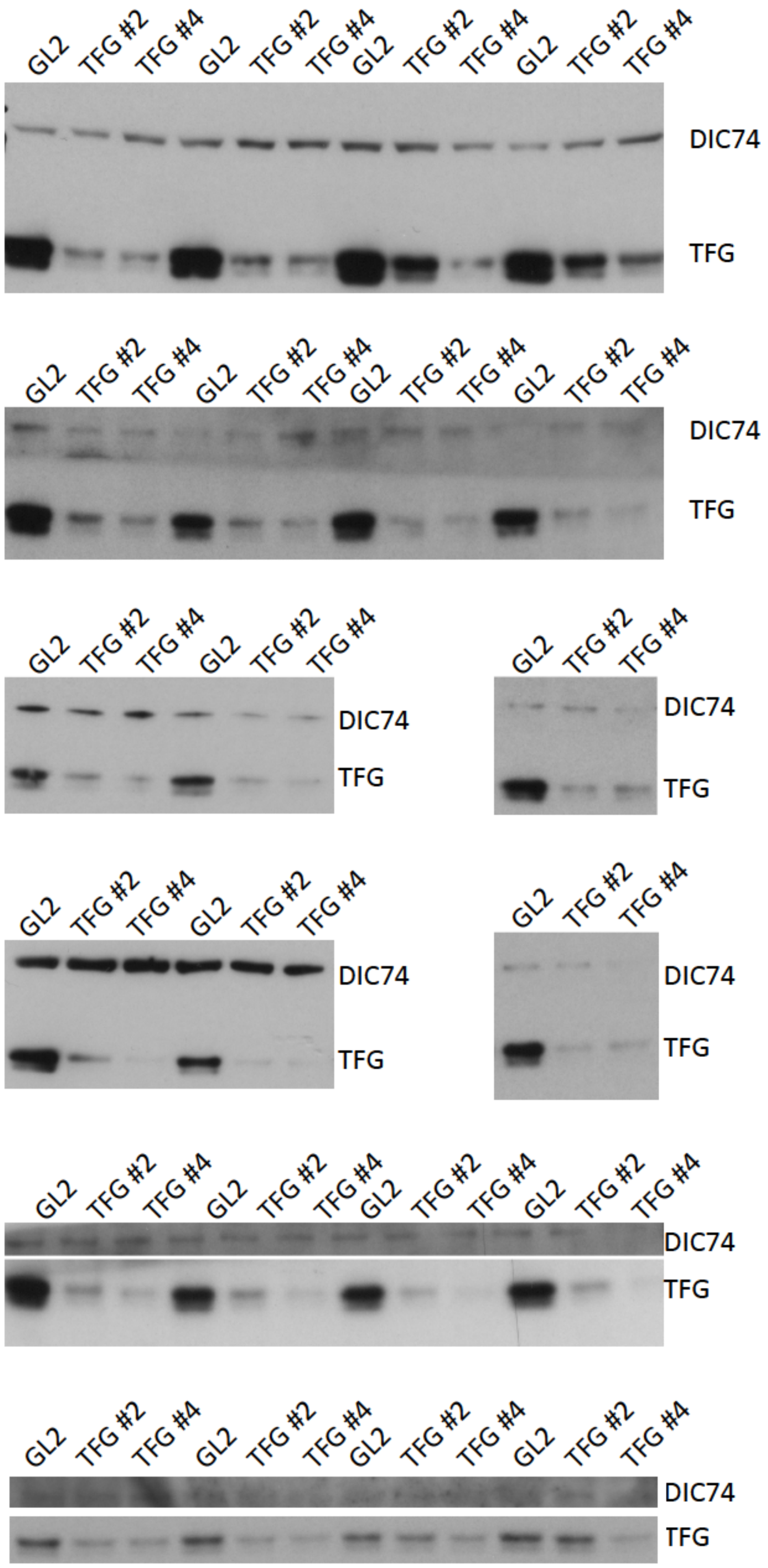
GL2 TFG #2 TFG #4 GL2 TFG #2 TFG #4 GL2 TFG #2 TFG #4 GL2 TFG #2 TFG #4
DIC74
TFG
GL2 TFG #2 TFG #4 GL2 TFG #2 TFG #4 GL2 TFG #2 TFG #4 GL2 TFG #2 TFG #4
DIC74
TFG
GL2 TFG #2 TFG #4 GL2 TFG #2 TFG #4
DIC74
TFG
GL2 TFG #2 TFG #4
DIC74
TFG
GL2 TFG #2 TFG #4 GL2 TFG #2 TFG #4
DIC74
TFG
GL2 TFG #2 TFG #4
DIC74
TFG
GL2 TFG #2 TFG #4 GL2 TFG #2 TFG #4 GL2 TFG #2 TFG #4 GL2 TFG #2 TFG #4
DIC74
TFG
GL2 TFG #2 TFG #4 GL2 TFG #2 TFG #4 GL2 TFG #2 TFG #4 GL2 TFG #2 TFG #4
DIC74
TFG

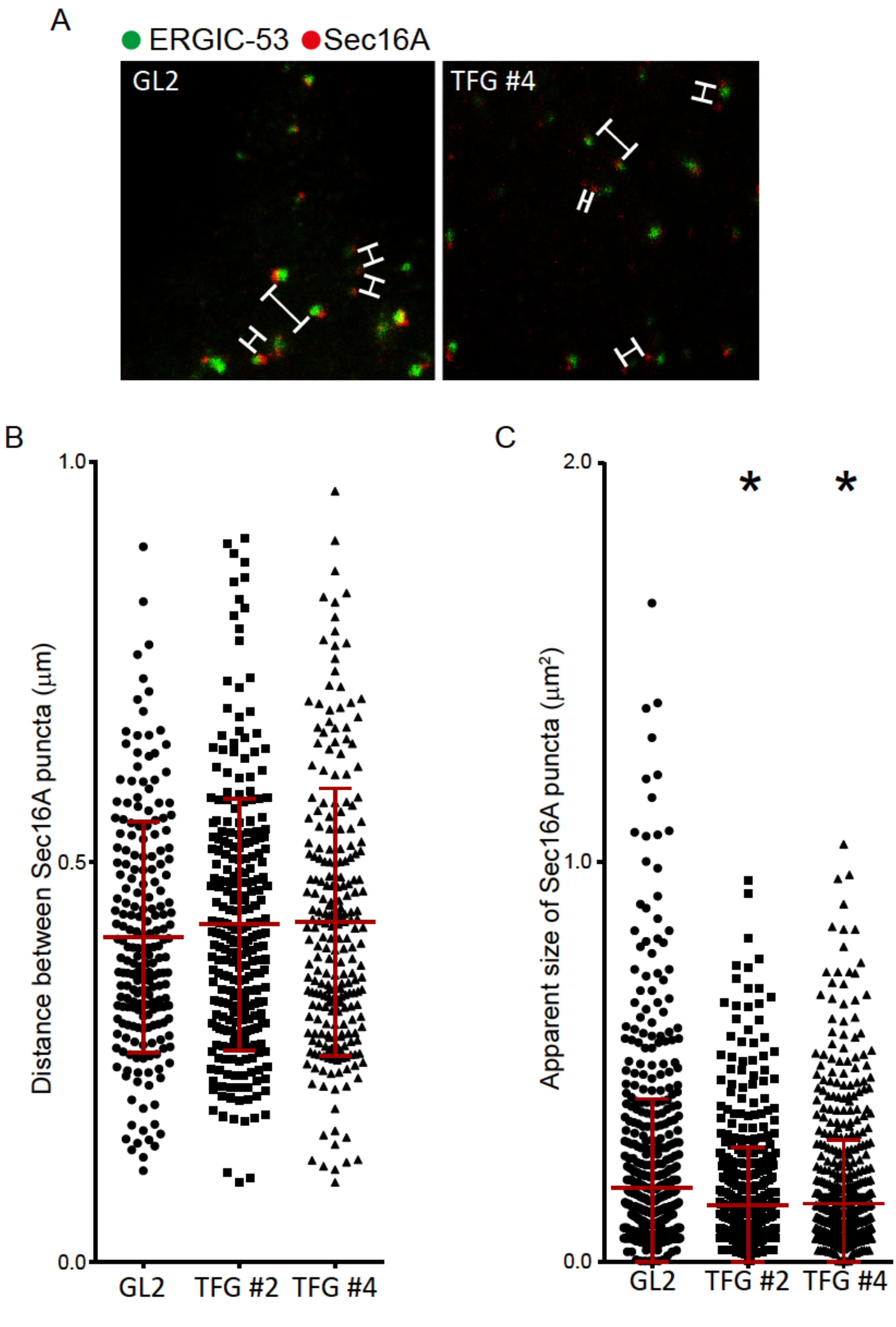
A
ERGIC-53
Sec16A
GL2
TFG #4
B
Distance between Sec16A puncta (µm)
1.0
0.5
0.0
GL2
TFG #2
TFG #4
C
Apparent size of Sec16A puncta (µm²)
2.0
1.0
0.0
*
*
GL2
TFG #2
TFG #4

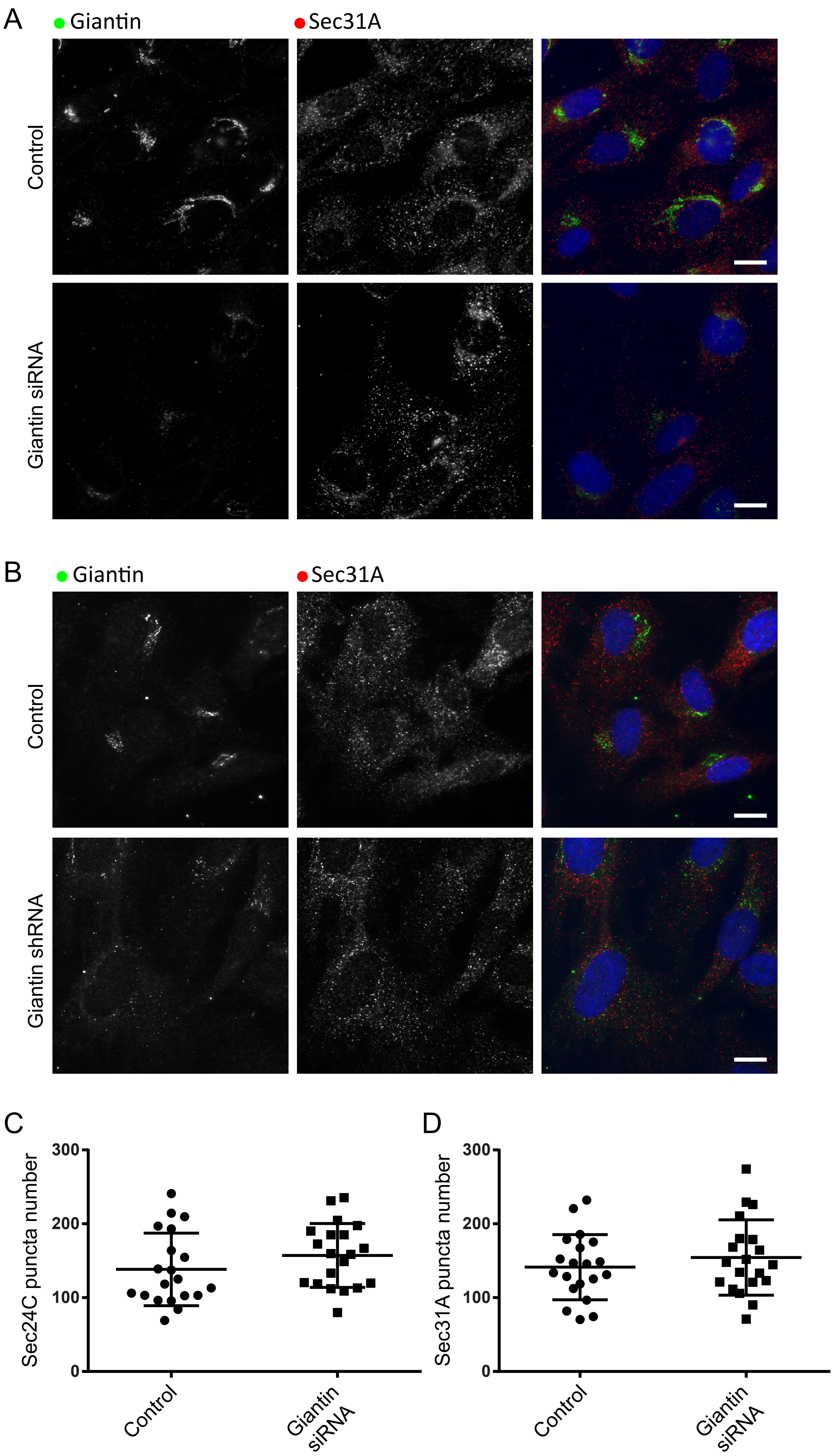
A
Giantin
Sec31A
Control
Giantin siRNA
B
Giantin
Sec31A
Control
Giantin shRNA
C
Sec24C puncta number
300
200
100
0
Control
Giantin siRNA
D
Sec31A puncta number
300
200
100
0
Control
Giantin siRNA

Legends to Supplemental Tables

Supplemental Table 1: List of proteomic hits from SILAC experiment showing those proteins identified in controls that were below the level of detection in both TFG-depleted and giantin-depleted samples. Highlighted in yellow are multiple key adhesion or matrix components. This identifies those proteins whose secretion and/or assembly into ECM is most affected by either TFG or giantin depletion. Data were filtered to remove proteins identified from a single peptide.

Supplemental Table 2: List of proteins that were decreased >3-fold more in TFG-depleted samples compared to giantin-depleted samples. This identifies those proteins who secretion and/or assembly in ECM depends more on TFG (tER organization) than giantin (Golgi organization). Highlighted in yellow are fibrillar collagens VI and I as well as collagen IV and other known collagen-binding proteins. Sec13 does appears high up on this list but we believe that this is related to its role in nuclear pore complexes for which see other components in this data set as well as other cell-derived matrix proteomes we have analysed.

Supplemental Table 1

Proteins that were detected in control but were below the level of detection following depletion of TFG.

ID	Description
TSP1_HUMAN	Thrombospondin-1 GN=THBS1 PE=1 SV=2 - [TSP1_HUMAN]
HSP7C_HUMAN	Heat shock cognate 71 kDa protein GN=HSPA8 PE=1 SV=1 - [HSP7C_HUMAN]
Q53GG0_HUMAN	Epithelial protein lost in neoplasm beta variant (Fragment) PE=2 SV=1 - [Q53GG0_HUMAN]
Q5TCU6_HUMAN	Talin 1 GN=TLN1 PE=2 SV=1 - [Q5TCU6_HUMAN]
K22E_HUMAN	Keratin, type II cytoskeletal 2 epidermal GN=KRT2 PE=1 SV=2 - [K22E_HUMAN]
K1C9_HUMAN	Keratin, type I cytoskeletal 9 GN=KRT9 PE=1 SV=3 - [K1C9_HUMAN]
K1C10_HUMAN	Keratin, type I cytoskeletal 10 GN=KRT10 PE=1 SV=6 - [K1C10_HUMAN]
B7Z2X4_HUMAN	cDNA FLJ53327, highly similar to Gelsolin PE=2 SV=1 - [B7Z2X4_HUMAN]
K1C16_HUMAN	Keratin, type I cytoskeletal 16 GN=KRT16 PE=1 SV=4 - [K1C16_HUMAN]
PLAK_HUMAN	Junction plakoglobin GN=JUP PE=1 SV=3 - [PLAK_HUMAN]
F6KPG5_HUMAN	Albumin (Fragment) PE=2 SV=1 - [F6KPG5_HUMAN]
I1VZV6_HUMAN	Hemoglobin alpha 1 GN=HBA1 PE=3 SV=1 - [I1VZV6_HUMAN]
DESP_HUMAN	Desmoplakin GN=DSP PE=1 SV=3 - [DESP_HUMAN]
DSG1_HUMAN	Desmoglein-1 GN=DSG1 PE=1 SV=2 - [DSG1_HUMAN]
F8WD96_HUMAN	Cathepsin D light chain GN=CTSD PE=3 SV=1 - [F8WD96_HUMAN]
J9ZVQ3_HUMAN	Apolipoprotein E (Fragment) GN=APOE PE=4 SV=1 - [J9ZVQ3_HUMAN]
B3KR41_HUMAN	Mitotic spindle assembly checkpoint protein MAD1 GN=MAD1L1 PE=2 SV=1 - [B3KR41_HUMAN]
K1C14_HUMAN	Keratin, type I cytoskeletal 14 GN=KRT14 PE=1 SV=4 - [K1C14_HUMAN]
K2C78_HUMAN	Keratin, type II cytoskeletal 78 GN=KRT78 PE=2 SV=2 - [K2C78_HUMAN]
TGM3_HUMAN	Protein-glutamine gamma-glutamyltransferase E GN=TGM3 PE=1 SV=4 - [TGM3_HUMAN]
Q5T985_HUMAN	Inter-alpha (Globulin) inhibitor H2 GN=ITIH2 PE=2 SV=1 - [Q5T985_HUMAN]
Q5JQ13_HUMAN	Vinculin (Fragment) GN=VCL PE=2 SV=1 - [Q5JQ13_HUMAN]
Q9NVF8_HUMAN	cDNA FLJ10762, moderately similar to CHLORINE CHANNEL PROTEIN P64 PE=2 SV=1 - [Q9NVF8_HUMAN]
Q0IIN1_HUMAN	Keratin 77 GN=KRT77 PE=2 SV=1 - [Q0IIN1_HUMAN]
FA5_HUMAN	Coagulation factor V GN=F5 PE=1 SV=4 - [FA5_HUMAN]
E9PIT3_HUMAN	Thrombin light chain GN=F2 PE=3 SV=1 - [E9PIT3_HUMAN]
LDHB_HUMAN	L-lactate dehydrogenase B chain GN=LDHB PE=1 SV=2 - [LDHB_HUMAN]
B4E054_HUMAN	cDNA FLJ58444, highly similar to Vacuolar ATP synthase subunit H PE=2 SV=1 - [B4E054_HUMAN]
B4DRW1_HUMAN	cDNA FLJ55805, highly similar to Keratin, type II cytoskeletal 4 PE=2 SV=1 - [B4DRW1_HUMAN]
Q9HB00_HUMAN	Desmocollin 1, isoform CRA_b GN=DSC1 PE=2 SV=1 - [Q9HB00_HUMAN]
S10A8_HUMAN	Protein S100-A8 GN=S100A8 PE=1 SV=1 - [S10A8_HUMAN]
Q1RMG2_HUMAN	Adenosylhomocysteinase GN=AHCY PE=2 SV=1 - [Q1RMG2_HUMAN]
PRDX1_HUMAN	Peroxiredoxin-1 GN=PRDX1 PE=1 SV=1 - [PRDX1_HUMAN]
E9PG15_HUMAN	14-3-3 protein theta (Fragment) GN=YWHAQ PE=4 SV=1 - [E9PG15_HUMAN]
I0B0K8_HUMAN	Truncated profilaggrin GN=FLG PE=3 SV=1 - [I0B0K8_HUMAN]
LPP3_HUMAN	Lipid phosphate phosphohydrolase 3 GN=PPAP2B PE=1 SV=1 - [LPP3_HUMAN]
LEG7_HUMAN	Galectin-7 GN=LGALS7 PE=1 SV=2 - [LEG7_HUMAN]
Q5HY40_HUMAN	Deoxyribonuclease-1-like 1 (Fragment) GN=DNASE1L1 PE=2 SV=1 - [Q5HY40_HUMAN]
F2Z393_HUMAN	Transaldolase GN=TALDO1 PE=3 SV=1 - [F2Z393_HUMAN]
K2C5_HUMAN	Keratin, type II cytoskeletal 5 GN=KRT5 PE=1 SV=3 - [K2C5_HUMAN]
K1C17_HUMAN	Keratin, type I cytoskeletal 17 GN=KRT17 PE=1 SV=2 - [K1C17_HUMAN]
CATA_HUMAN	Catalase GN=CAT PE=1 SV=3 - [CATA_HUMAN]
FILA2_HUMAN	Filaggrin-2 GN=FLG2 PE=1 SV=1 - [FILA2_HUMAN]
SPTB1_HUMAN	Spectrin beta chain, erythrocytic GN=SPTB PE=1 SV=5 - [SPTB1_HUMAN]
SPB12_HUMAN	Serpin B12 GN=SERPINB12 PE=1 SV=1 - [SPB12_HUMAN]
EHD3_HUMAN	EH domain-containing protein 3 GN=EHD3 PE=1 SV=2 - [EHD3_HUMAN]

FETUA_HUMAN	Alpha-2-HS-glycoprotein GN=AHSG PE=1 SV=1 - [FETUA_HUMAN]
HUTH_HUMAN	Histidine ammonia-lyase GN=HAL PE=1 SV=1 - [HUTH_HUMAN]
B4DF70_HUMAN	cDNA FLJ60461, highly similar to Peroxiredoxin-2 (EC 1.11.1.15) PE=2 SV=1 - [B4DF70_HUMAN]
F5H308_HUMAN	L-lactate dehydrogenase GN=LDHA PE=3 SV=1 - [F5H308_HUMAN]
Q8NBH6_HUMAN	cDNA PSEC0266 fis, clone NT2RP3003649, highly similar to Homo sapiens fibulin-1D mRNA PE=2 SV=1 - [Q8NBH6_HUMAN]
ARGI1_HUMAN	Arginase-1 GN=ARG1 PE=1 SV=2 - [ARGI1_HUMAN]
PSA3_HUMAN	Proteasome subunit alpha type-3 GN=PSMA3 PE=1 SV=2 - [PSA3_HUMAN]
APOC3_HUMAN	Apolipoprotein C-III GN=APOC3 PE=1 SV=1 - [APOC3_HUMAN]
B4E147_HUMAN	cDNA FLJ50342, highly similar to Myosin-5A (Fragment) PE=2 SV=1 - [B4E147_HUMAN]

Supplemental Table 2
Proteins that decreased >3-fold more following depletion of TFG than depletion of giantin.

Ranked according to the most affected first.

Gene name	Description
Q5U0B9_HUMAN	Stem cell growth factor; lymphocyte secreted C-type lectin GN=CLEC11A PE=2 SV=1 - [Q5U0B9_HUMAN]
A8MV37_HUMAN	Protein SEC13 homolog GN=SEC13 PE=4 SV=1 - [A8MV37_HUMAN]
HSPB1_HUMAN	Heat shock protein beta-1 GN=HSPB1 PE=1 SV=2 - [HSPB1_HUMAN]
B7Z641_HUMAN	V-type proton ATPase 116 kDa subunit a isoform 1 GN=ATP6V0A1 PE=2 SV=1 - [B7Z641_HUMAN]
B4DNM8_HUMAN	cDNA FLJ53395, highly similar to Prolyl 3-hydroxylase 1 (EC 1.14.11.7) PE=2 SV=1 - [B4DNM8_HUMAN]
B7Z381_HUMAN	cDNA FLJ55066, highly similar to LAS1-like protein PE=2 SV=1 - [B7Z381_HUMAN]
NOL6_HUMAN	Nucleolar protein 6 GN=NOL6 PE=1 SV=2 - [NOL6_HUMAN]
SYNEM_HUMAN	Synemin GN=SYNM PE=1 SV=2 - [SYNEM_HUMAN]
Q4W4Y1_HUMAN	Dopamine receptor interacting protein 4 GN=DRIP4 PE=2 SV=1 - [Q4W4Y1_HUMAN]
E7ESV4_HUMAN	Ras-related protein Rap-1b (Fragment) GN=RAP1B PE=4 SV=1 - [E7ESV4_HUMAN]
RAN_HUMAN	GTP-binding nuclear protein Ran GN=RAN PE=1 SV=3 - [RAN_HUMAN]
B4DUI5_HUMAN	Triosephosphate isomerase PE=2 SV=1 - [B4DUI5_HUMAN]
Q6PJT4_HUMAN	MSN protein (Fragment) GN=MSN PE=2 SV=1 - [Q6PJT4_HUMAN]
H3BTN5_HUMAN	Pyruvate kinase (Fragment) GN=PKM PE=3 SV=1 - [H3BTN5_HUMAN]
B2RDI5_HUMAN	cDNA, FLJ96627, highly similar to Homo sapiens calpain 1, (mu/I) large subunit (CAPN1), mRNA PE=2 SV=1 - [B2RDI5_HUMAN]
B4DN87_HUMAN	cDNA FLJ52569, highly similar to Collagen-binding protein 2 PE=2 SV=1 - [B4DN87_HUMAN]
RAB8A_HUMAN	Ras-related protein Rab-8A GN=RAB8A PE=1 SV=1 - [RAB8A_HUMAN]
CO6A1_HUMAN	Collagen alpha-1(VI) chain GN=COL6A1 PE=1 SV=3 - [CO6A1_HUMAN]
CO6A2_HUMAN	Collagen alpha-2(VI) chain GN=COL6A2 PE=1 SV=4 - [CO6A2_HUMAN]
B3KVN0_HUMAN	cDNA FLJ16785 fis, clone NT2RI2015342, highly similar to Solute carrier family 2, facilitated glucose transporter member 1 PE=2 SV=1 - [B3KVN0_HUMAN]
B4DT13_HUMAN	Ras-related protein Rab-11A GN=RAB11A PE=2 SV=1 - [B4DT13_HUMAN]
CO6A3_HUMAN	Collagen alpha-3(VI) chain GN=COL6A3 PE=1 SV=5 - [CO6A3_HUMAN]
TIMP3_HUMAN	Metalloproteinase inhibitor 3 GN=TIMP3 PE=1 SV=2 - [TIMP3_HUMAN]
LAMA5_HUMAN	Laminin subunit alpha-5 GN=LAMA5 PE=1 SV=8 - [LAMA5_HUMAN]
I6L965_HUMAN	KRT18 protein (Fragment) GN=KRT18 PE=2 SV=1 - [I6L965_HUMAN]
Q59GA0_HUMAN	Thy-1 cell surface antigen variant (Fragment) PE=2 SV=1 - [Q59GA0_HUMAN]
ARF6_HUMAN	ADP-ribosylation factor 6 GN=ARF6 PE=1 SV=2 - [ARF6_HUMAN]
Q59F15_HUMAN	COL4A1 protein variant (Fragment) PE=2 SV=1 - [Q59F15_HUMAN]
CO4A2_HUMAN	Collagen alpha-2(IV) chain GN=COL4A2 PE=1 SV=4 - [CO4A2_HUMAN]
NEXN_HUMAN	Nexilin GN=NEXN PE=1 SV=1 - [NEXN_HUMAN]
E9PCY7_HUMAN	Heterogeneous nuclear ribonucleoprotein H GN=HNRNPH1 PE=4 SV=1 - [E9PCY7_HUMAN]
ANXA2_HUMAN	Annexin A2 GN=ANXA2 PE=1 SV=2 - [ANXA2_HUMAN]
CO1A2_HUMAN	Collagen alpha-2(I) chain GN=COL1A2 PE=1 SV=7 - [CO1A2_HUMAN]
K7EM73_HUMAN	Calpain small subunit 1 (Fragment) GN=CAPNS1 PE=4 SV=1 - [K7EM73_HUMAN]
B4DM82_HUMAN	cDNA FLJ53060, moderately similar to Peptidyl-prolyl cis-trans isomerase A (EC 5.2.1.8) PE=2 SV=1 - [B4DM82_HUMAN]
DKC1_HUMAN	H/ACA ribonucleoprotein complex subunit 4 GN=DKC1 PE=1 SV=3 - [DKC1_HUMAN]
Q65ZQ3_HUMAN	FBRNP GN=D10S102 PE=2 SV=1 - [Q65ZQ3_HUMAN]
D6R904_HUMAN	Tropomyosin alpha-3 chain GN=TPM3 PE=4 SV=1 - [D6R904_HUMAN]
B4E3T7_HUMAN	cDNA FLJ55448, highly similar to Nuclear pore complex protein Nup133 PE=2 SV=1 - [B4E3T7_HUMAN]

DDX21_HUMAN	Nucleolar RNA helicase 2 GN=DDX21 PE=1 SV=5 - [DDX21_HUMAN]
CLH1_HUMAN	Clathrin heavy chain 1 GN=CLTC PE=1 SV=5 - [CLH1_HUMAN]
A8K7F6_HUMAN	cDNA FLJ78244, highly similar to Homo sapiens eukaryotic translation initiation factor 4A, isoform 1 (EIF4A1), mRNA PE=2 SV=1 - [A8K7F6_HUMAN]
B2R6X5_HUMAN	cDNA, FLJ93166, highly similar to Homo sapiens heat shock 70kDa protein 6 (HSP70B') (HSPA6), mRNA PE=2 SV=1 - [B2R6X5_HUMAN]
LMNB1_HUMAN	Lamin-B1 GN=LMNB1 PE=1 SV=2 - [LMNB1_HUMAN]
E9PDF6_HUMAN	Unconventional myosin-Ib GN=MYO1B PE=4 SV=1 - [E9PDF6_HUMAN]
A4QPB0_HUMAN	IQ motif containing GTPase activating protein 1 GN=IQGAP1 PE=2 SV=1 - [A4QPB0_HUMAN]
CATK_HUMAN	Cathepsin K GN=CTSK PE=1 SV=1 - [CATK_HUMAN]
PGBM_HUMAN	Basement membrane-specific heparan sulfate proteoglycan core protein GN=HSPG2 PE=1 SV=4 - [PGBM_HUMAN]
B1AK87_HUMAN	Capping protein (Actin filament) muscle Z-line, beta GN=CAPZB PE=4 SV=1 - [B1AK87_HUMAN]
GLE1_HUMAN	Nucleoporin GLE1 GN=GLE1 PE=1 SV=2 - [GLE1_HUMAN]
LMNA_HUMAN	Prelamin-A/C GN=LMNA PE=1 SV=1 - [LMNA_HUMAN]
ARC1B_HUMAN	Actin-related protein 2/3 complex subunit 1B GN=ARPC1B PE=1 SV=3 - [ARC1B_HUMAN]
NOP2_HUMAN	Putative ribosomal RNA methyltransferase NOP2 GN=NOP2 PE=1 SV=2 - [NOP2_HUMAN]
ARPC4_HUMAN	Actin-related protein 2/3 complex subunit 4 GN=ARPC4 PE=1 SV=3 - [ARPC4_HUMAN]
Q96BS4_HUMAN	FBL protein (Fragment) GN=FBL PE=2 SV=2 - [Q96BS4_HUMAN]
Q14784_HUMAN	Myosin (Fragment) PE=2 SV=1 - [Q14784_HUMAN]
ARP3_HUMAN	Actin-related protein 3 GN=ACTR3 PE=1 SV=3 - [ARP3_HUMAN]
A8K0T9_HUMAN	cDNA FLJ75422, highly similar to Homo sapiens capping protein (actin filament) muscle Z-line, alpha 1, mRNA PE=2 SV=1 - [A8K0T9_HUMAN]
B7ZAF0_HUMAN	cDNA, FLJ79164, highly similar to Tubulin beta-7 chain PE=2 SV=1 - [B7ZAF0_HUMAN]
ARP2_HUMAN	Actin-related protein 2 GN=ACTR2 PE=1 SV=1 - [ARP2_HUMAN]
ARPC2_HUMAN	Actin-related protein 2/3 complex subunit 2 GN=ARPC2 PE=1 SV=1 - [ARPC2_HUMAN]
PLEC_HUMAN	Plectin GN=PLEC PE=1 SV=3 - [PLEC_HUMAN]
SDPR_HUMAN	Serum deprivation-response protein GN=SDPR PE=1 SV=3 - [SDPR_HUMAN]
PCBP1_HUMAN	Poly(rC)-binding protein 1 GN=PCBP1 PE=1 SV=2 - [PCBP1_HUMAN]
C9J0I9_HUMAN	Nuclear-interacting partner of ALK GN=ZC3HC1 PE=4 SV=1 - [C9J0I9_HUMAN]
1433E_HUMAN	14-3-3 protein epsilon GN=YWHAE PE=1 SV=1 - [1433E_HUMAN]
EF1A1_HUMAN	Elongation factor 1-alpha 1 GN=EEF1A1 PE=1 SV=1 - [EF1A1_HUMAN]
PRP6_HUMAN	Pre-mRNA-processing factor 6 GN=PRPF6 PE=1 SV=1 - [PRP6_HUMAN]
B3GQE6_HUMAN	DEAD box polypeptide 27 GN=DDX27 PE=2 SV=1 - [B3GQE6_HUMAN]
C9J9K3_HUMAN	40S ribosomal protein SA (Fragment) GN=RPSA PE=3 SV=1 - [C9J9K3_HUMAN]
VIME_HUMAN	Vimentin GN=VIM PE=1 SV=4 - [VIME_HUMAN]
B5BU24_HUMAN	14-3-3 protein beta/alpha GN=YWHAB PE=2 SV=1 - [B5BU24_HUMAN]
B7Z1R5_HUMAN	V-type proton ATPase catalytic subunit A GN=ATP6V1A PE=2 SV=1 - [B7Z1R5_HUMAN]
H3BM89_HUMAN	60S ribosomal protein L4 GN=RPL4 PE=4 SV=1 - [H3BM89_HUMAN]
SMOC1_HUMAN	SPARC-related modular calcium-binding protein 1 GN=SMOC1 PE=1 SV=1 - [SMOC1_HUMAN]
E9PF58_HUMAN	Actin-related protein 2/3 complex subunit 1A GN=ARPC1A PE=4 SV=1 - [E9PF58_HUMAN]
RPF1_HUMAN	Ribosome production factor 1 GN=RPF1 PE=1 SV=2 - [RPF1_HUMAN]
B7Z9C0_HUMAN	cDNA FLJ52405, highly similar to Myosin Ic PE=2 SV=1 - [B7Z9C0_HUMAN]
F8VWC5_HUMAN	60S ribosomal protein L18 GN=RPL18 PE=3 SV=1 - [F8VWC5_HUMAN]
I3L1L3_HUMAN	Myb-binding protein 1A (Fragment) GN=MYBBP1A PE=4 SV=1 - [I3L1L3_HUMAN]
B3KS36_HUMAN	cDNA FLJ35376 fis, clone SKMUS2004044, highly similar to Homo sapiens ribosomal protein L3 (RPL3), transcript variant 2, mRNA PE=2 SV=1 - [B3KS36_HUMAN]
B4DT35_HUMAN	Nucleoporin p54 GN=NUP54 PE=2 SV=1 - [B4DT35_HUMAN]
FLOT1_HUMAN	Flotillin-1 GN=FLOT1 PE=1 SV=3 - [FLOT1_HUMAN]

D3DN97_HUMAN	Myosin, light polypeptide kinase, isoform CRA_d GN=MYLK PE=4 SV=1 - [D3DN97_HUMAN]
VATB2_HUMAN	V-type proton ATPase subunit B, brain isoform GN=ATP6V1B2 PE=1 SV=3 - [VATB2_HUMAN]
B3KN82_HUMAN	cDNA FLJ13913 fis, clone Y79AA1000231, highly similar to Nucleolar protein NOP5 PE=2 SV=1 - [B3KN82_HUMAN]
B7Z5H9_HUMAN	cDNA FLJ53291, highly similar to Beta-galactosidase-related protein PE=2 SV=1 - [B7Z5H9_HUMAN]
B4E0K0_HUMAN	cDNA FLJ52820, highly similar to Nucleoporin p58/p45 PE=2 SV=1 - [B4E0K0_HUMAN]
MRT4_HUMAN	mRNA turnover protein 4 homolog GN=MRTO4 PE=1 SV=2 - [MRT4_HUMAN]
B2RAX6_HUMAN	cDNA, FLJ95176, Homo sapiens CGI-48 protein (CGI-48), mRNA PE=2 SV=1 - [B2RAX6_HUMAN]
H12_HUMAN	Histone H1.2 GN=HIST1H1C PE=1 SV=2 - [H12_HUMAN]
CAV1_HUMAN	Caveolin-1 GN=CAV1 PE=1 SV=4 - [CAV1_HUMAN]
E9PMV1_HUMAN	Plectin (Fragment) GN=PLEC PE=4 SV=1 - [E9PMV1_HUMAN]
C9J592_HUMAN	Ras-related protein Rab-7a (Fragment) GN=RAB7A PE=3 SV=1 - [C9J592_HUMAN]
A4FU77_HUMAN	SNRNP200 protein (Fragment) GN=SNRNP200 PE=2 SV=1 - [A4FU77_HUMAN]
WDR12_HUMAN	Ribosome biogenesis protein WDR12 GN=WDR12 PE=1 SV=2 - [WDR12_HUMAN]
H0Y6E7_HUMAN	RNA-binding motif protein, X chromosome, N-terminally processed (Fragment) GN=RBMX PE=4 SV=2 - [H0Y6E7_HUMAN]
DDX18_HUMAN	ATP-dependent RNA helicase DDX18 GN=DDX18 PE=1 SV=2 - [DDX18_HUMAN]
IF6_HUMAN	Eukaryotic translation initiation factor 6 GN=EIF6 PE=1 SV=1 - [IF6_HUMAN]
Q9H9J5_HUMAN	cDNA FLJ12694 fis, clone NT2RP1000358, highly similar to Homo sapiens mRNA; cDNA DKFZp564C186 (from clone DKFZp564C186) PE=2 SV=1 - [Q9H9J5_HUMAN]
B4DKM5_HUMAN	Voltage-dependent anion-selective channel protein 2 GN=VDAC2 PE=2 SV=1 - [B4DKM5_HUMAN]
K7EMV3_HUMAN	Histone H3.3 GN=H3F3B PE=4 SV=1 - [K7EMV3_HUMAN]
HNRPM_HUMAN	Heterogeneous nuclear ribonucleoprotein M GN=HNRNPM PE=1 SV=3 - [HNRPM_HUMAN]
B4DHC4_HUMAN	cDNA FLJ51843, highly similar to 14-3-3 protein gamma PE=2 SV=1 - [B4DHC4_HUMAN]
RBM8A_HUMAN	RNA-binding protein 8A GN=RBM8A PE=1 SV=1 - [RBM8A_HUMAN]
Q5JR95_HUMAN	40S ribosomal protein S8 GN=RPS8 PE=4 SV=1 - [Q5JR95_HUMAN]
D9YZV7_HUMAN	Tropomyosin 1 (Alpha) isoform 6 GN=TPM1 PE=3 SV=1 - [D9YZV7_HUMAN]
B4E3M6_HUMAN	cDNA FLJ55446, highly similar to Superkiller viralicidic activity 2-like 2 (EC 3.6.1.-) PE=2 SV=1 - [B4E3M6_HUMAN]
B7Z5I6_HUMAN	cDNA FLJ56516, highly similar to Nuclear pore complex protein Nup88 PE=2 SV=1 - [B7Z5I6_HUMAN]
B1ARP8_HUMAN	Mago-nashi homolog, proliferation-associated (Drosophila) GN=MAGOH PE=4 SV=1 - [B1ARP8_HUMAN]
A8K330_HUMAN	cDNA FLJ77055, highly similar to Homo sapiens WD repeat domain 75 (WDR75), mRNA (Fragment) PE=2 SV=1 - [A8K330_HUMAN]
VATG1_HUMAN	V-type proton ATPase subunit G 1 GN=ATP6V1G1 PE=1 SV=3 - [VATG1_HUMAN]
B5BUB1_HUMAN	RuvB-like 1 (Fragment) GN=RUVBL1 PE=2 SV=1 - [B5BUB1_HUMAN]
A8K897_HUMAN	cDNA FLJ78686, highly similar to Homo sapiens nucleoporin 93kDa (NUP93), mRNA PE=2 SV=1 - [A8K897_HUMAN]
NH2L1_HUMAN	NHP2-like protein 1 GN=NHP2L1 PE=1 SV=3 - [NH2L1_HUMAN]

Legend to Supplemental Note

Supplemental note: Matlab code for STED image analysis: The code for Matlab that we used to analyse the STED data are included as a supplemental file. A short description of how to implement it follows.

Supplemental note:

Instructions for use of Matlab code from McCaughey et al.

Organisation and installation

The analysis code allows the user to perform automatic analysis over multiple datasets. Images should be in TIFF format and contain a channel identifier within the name (C=0 for channel 0 and C=1 for channel 1). Each experiment can be placed in a separate folder, with multiple images allowed within each folder. The code makes use of an adaptive thresholding routine created by Guangeli Xiong which is available through the Matlab Central file exchange (http://uk.mathworks.com/matlabcentral/fileexchange/8647-local-adaptive-thresholding/content/adaptivethreshold/adaptivethreshold.m) users should download this file, unzip it and place it somewhere on the Matlab path prior to running the analysis code.

Users should navigate (1) to show the list of folders to be analysed in the "current folder" window of Matlab (2) as shown below:

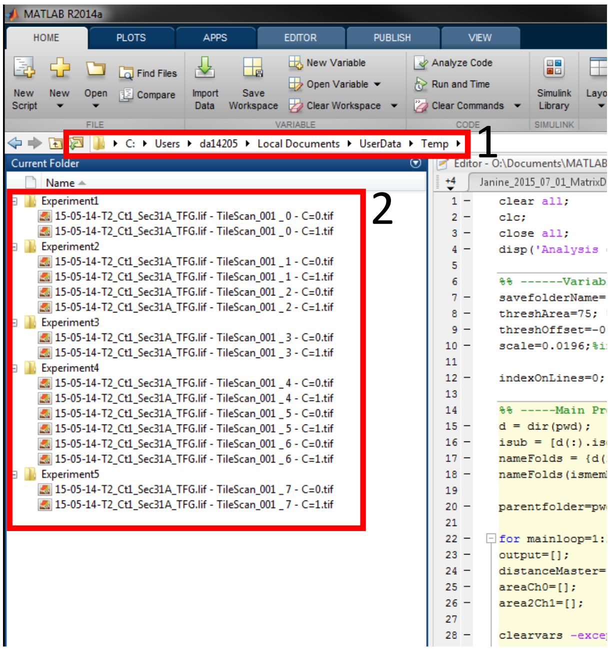

Running of code

At the top of the .m file is a section called "Variables to edit".

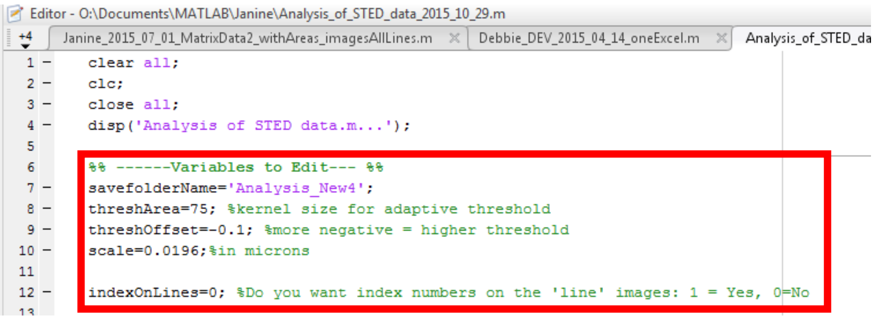
Editor - O:\Documents\MATLAB\Janine\Analysis_of_STED_data_2015_10_29.m

Janine_2015_07_01_MatrixData2_withAreas_imagesAllLines.m | Debbie_DEV_2015_04_14_oneExcel.m | Analysis_of_STED_da

```
clear all;
clc;
close all;
disp('Analysis of STED data.m...');

%% ------Variables to Edit--- %%
savefolderName='Analysis_New4';
threshArea=75; %kernel size for adaptive threshold
threshOffset=-0.1; %more negative = higher threshold
scale=0.0196;%in microns

indexOnLines=0; %Do you want index numbers on the 'line' images: 1 = Yes, 0=No
```

Here the user may adjust thresholding parameters used in the segmentation as well as the scale (pixel size) of the images used. Note the programme assumes the same pixel size across all images. If images with different pixel sizes were acquired, these would need to be analysed independently. Below is a summary of the adjustable variables and their meaning.

Variable name	description
savefolderName	Name of results folder (one created per experiment folder)
threshArea	Kernel size for adaptive threshold
threshOffset	Adjustment to automatic thresholding, more negative= higher threshold
scale	Pixel size in microns
indexOnLines	Display index numbers on images. 1=Yes, 0=No.

More variable can be found throughout the main programme (size of Weiner kernel etc.) which the user is free to edit, but these have been tuned for this particular dataset and were therefore kept constant.

Once the required variable have been inputted, the user simply runs the entire code by clicking "Run" whilst on the Editor tab.

<u>Data Output</u>

For each field of view the programme create three images which are saved with the field of view name plus one of the following:

1. "filteredMerge2" – shows the smoothed data, overlaid with the indexes of the detected objects and the lines joining any neighbouring objects.
2. "layered" – shows each layer of the above image on a separate page of a multipage TIFF file.
3. "originalMerge2" – shows the original (unsmoothed) data overlaid with the indexes o of the detected objects and the lines joining any neighbouring objects.

The programme also creates an excel spreadsheet for each field of view analysed containing tabs for the measure area of the objects and the distance between objects, all values are given in microns. The name given to the excel file will be the same as the folder containing the images

Legends to Supplemental Movies

Supplemental Movie 1: ER-to-Golgi transport of mannosidase II-GFP in control (GL2 siRNA-transfected) cells.

Imaging of biotin-dependent export of mannII-GFP from the ER in cells expressing GRASP65-mCherry to monitor delivery of cargo from the ER to the Golgi in control (GL2 siRNA-transfected) cells.

Supplemental Movie 2: ER-to-Golgi transport of mannosidase II-GFP in TFG-depleted cells.

Imaging of biotin-dependent export of mannII-GFP from the ER in cells expressing GRASP65-mCherry to monitor delivery of cargo from the ER to the Golgi in TFG-depleted cells.
