# Supplementary figures and images for: TFG Promotes Organization of Transitional ER and Efficient Collagen Secretion
Source: Cell Rep. 2016 May 12;15(8):1648–59. doi: 10.1016/j.celrep.2016.04.062 (PMC4885023; doi:10.1016/j.celrep.2016.04.062)

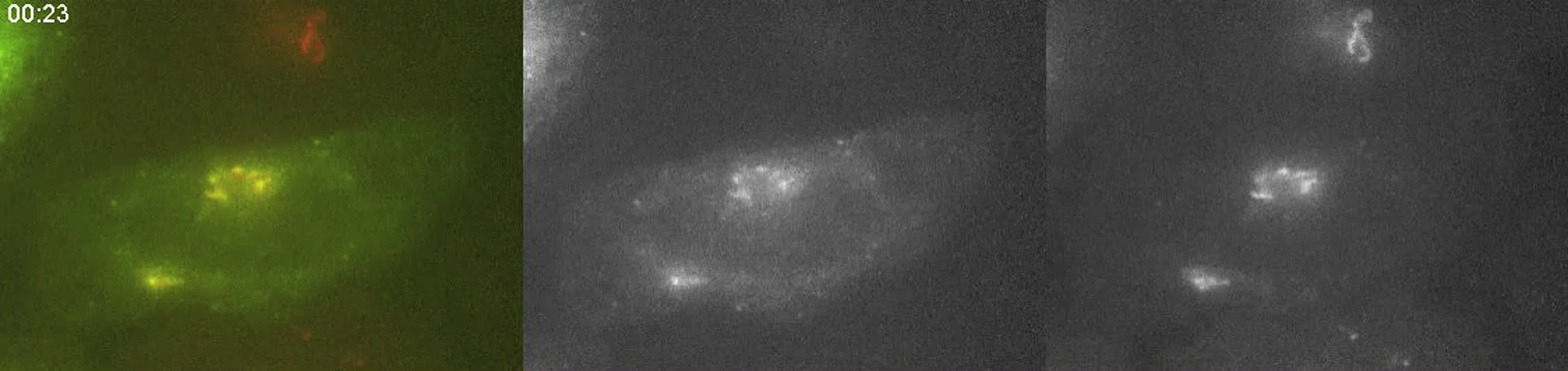

Supplement: Movie S1. ER-to-Golgi Transport of Mannosidase II-GFP in GL2 siRNA-Transfected Cells, Related to Figure 3 [file mmc2.jpg]

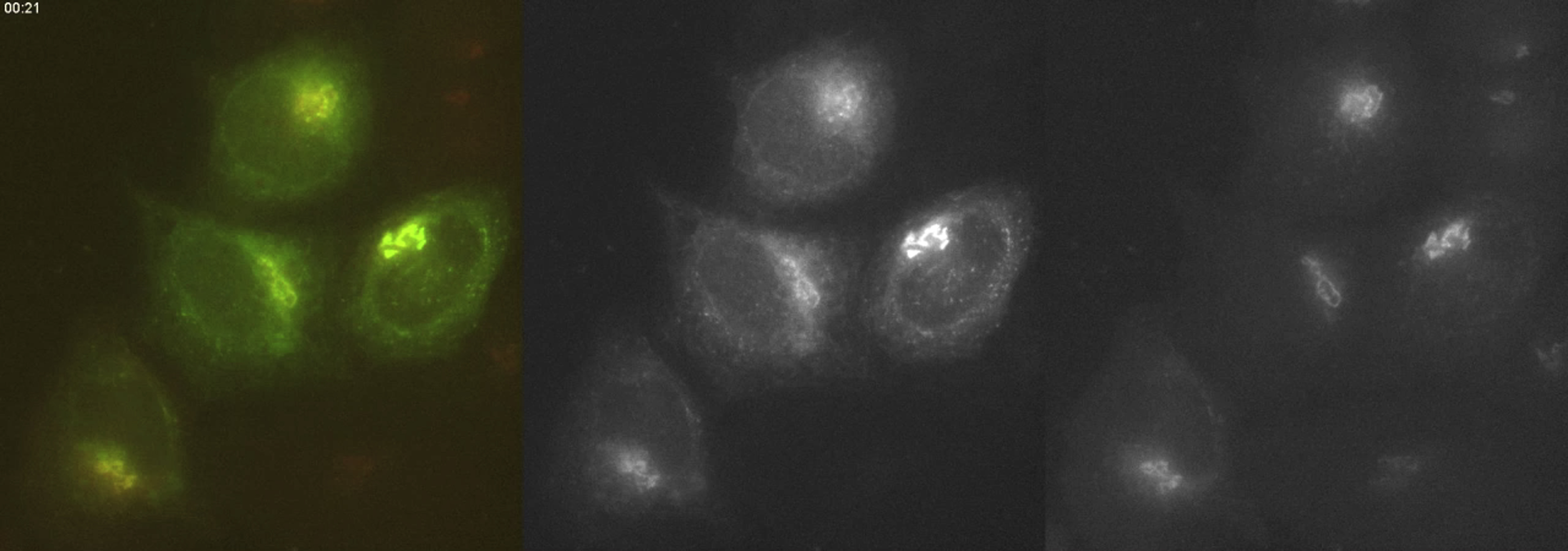

Supplement: Movie S2. ER-to-Golgi Transport of Mannosidase II-GFP in TFG-Depleted Cells, Related to Figure 3 [file mmc3.jpg]
