# Supplementary material for: TFG Promotes Organization of Transitional ER and Efficient Collagen Secretion
Source: Cell Rep. 2016 May 12;15(8):1648–59. doi: 10.1016/j.celrep.2016.04.062 (PMC4885023; doi:10.1016/j.celrep.2016.04.062)
Supplement: Document S2. Article plus Supplemental Information [file mmc5.pdf]

Cell Reports

Article

TFG Promotes Organization of Transitional ER and Efficient Collagen Secretion

Graphical Abstract

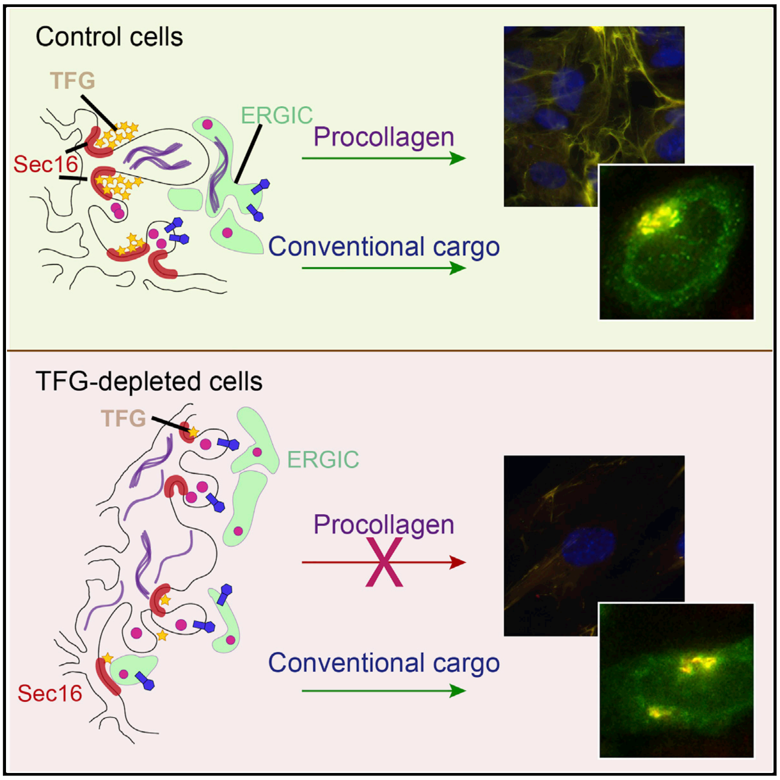

Authors

Janine McCaughey, Victoria J. Miller, Nicola L. Stevenson, ..., Kate J. Heesom, Dominic Alibhai, David J. Stephens

Correspondence

david.stephens@bristol.ac.uk

In Brief

McCaughey et al. show that TFG is required to support the organization of ER exit sites (ERESs) into larger structures. This higher-order organization is required for efficient secretion of procollagen.

Highlights

- TFG is required to organize transitional ER into larger structures
- Following depletion of TFG, ERESs remain in close apposition to the ERGIC
- Mini-ERESs support secretion of small soluble cargo
- Large ERESs are required for procollagen secretion

McCaughey et al., 2016, Cell Reports *15*, 1648–1659
May 24, 2016 © 2016 The Author(s)
http://dx.doi.org/10.1016/j.celrep.2016.04.062

OPEN ACCESS
CellPress

Cell Reports
Article

TFG Promotes Organization of Transitional ER and Efficient Collagen Secretion

Janine McCaughey,[1,2,5] Victoria J. Miller,[1,5] Nicola L. Stevenson,[1] Anna K. Brown,[1] Annika Budnik,[1] Kate J. Heesom,[4] Dominic Alibhai,[3] and David J. Stephens[1,*]
[1]Cell Biology Laboratories, School of Biochemistry, Faculty of Biomedical Sciences, University of Bristol, University Walk, Bristol BS8 1TD, UK
[2]Institut für Biophysik, Leibniz Universität Hannover, Herrenhäuserstraβe 2, 30419 Hannover, Germany
[3]Wolfson Bioimaging Facility, Faculty of Biomedical Sciences, University of Bristol, University Walk, Bristol BS8 1TD, UK
[4]Proteomics Facility, Faculty of Biomedical Sciences, University of Bristol, University Walk, Bristol BS8 1TD, UK
[5]Co-first author
*Correspondence: david.stephens@bristol.ac.uk
http://dx.doi.org/10.1016/j.celrep.2016.04.062

SUMMARY

Collagen is the most abundant protein in the animal kingdom. It is of fundamental importance during development for cell differentiation and tissue morphogenesis as well as in pathological processes such as fibrosis and cancer cell migration. However, our understanding of the mechanisms of procollagen secretion remains limited. Here, we show that TFG organizes transitional ER (tER) and ER exit sites (ERESs) into larger structures. Depletion of TFG results in dispersion of tER elements that remain associated with individual ER-Golgi intermediate compartments (ERGICs) as largely functional ERESs. We show that TFG is not required for the transport and packaging of small soluble cargoes but is necessary for the export of procollagen from the ER. Our work therefore suggests a key relationship between the structure and function of ERESs and a central role for TFG in optimizing COPII assembly for procollagen export.

INTRODUCTION

Collagen is the most abundant protein in the animal kingdom. Its secretion and assembly into a fibrillar extracellular matrix underpins core aspects of development such as directed cell migration, tissue morphogenesis, cell polarization, and bone formation. The fidelity of continued secretion and assembly of collagen throughout life is central to its role in key pathological states including fibrosis and cancer cell invasion. The earliest stages of procollagen assembly take place within the ER (Canty and Kadler, 2005). Fibril-forming collagens such as types I, II, III, V, XI, XXIV, and XXVII assemble as large trimeric structures within the ER that must then be packaged into vesicular carriers for transport to the Golgi and onward to the extracellular space (Mouw et al., 2014). This packaging of cargo and formation of vesicles at the ER membrane is mediated by the COPII complex.

COPII-dependent export of secretory cargo from the ER is essential for the normal function of all mammalian cells (Zanetti et al., 2011). COPII acts at specific sites on the ER membrane defined as transitional ER (tER). COPII drives the concentration of secretory cargo, deformation of the membrane, and subsequent formation of COPII-coated vesicles and tubules. COPII vesicles bud from the tER but remain localized within the immediate vicinity of it. These membrane carriers subsequently form fuse with (or fuse to form) the first post-ER compartment, the ER-Golgi intermediate compartment (ERGIC). These three structures, the tER, COPII-coated membranes, and the ERGIC, together form a unit known as an ER exit site (ERES). These cup-shaped structures (Bannykh et al., 1996) consist of a defined tER membrane with which Sec16 is closely associated (Hughes et al., 2009).

The process for the formation of COPII-coated vesicles is described classically as Sec12-dependent loading of GTP onto Sar1 followed by sequential recruitment of Sec23-Sec24 and Sec13-Sec31 and is sufficient for the generation of small 60- to 80- nm vesicles in vitro (Zanetti et al., 2011). Although these vesicles can accommodate the majority of cargo, additional flexibility is required, both physically and in terms of the completion of budding, to encapsulate larger cargo such as procollagen (Malhotra and Erlmann, 2011). Fibrillar collagen precursors (such as procollagen I at 300 nm; Bächinger et al., 1982) are too large to fit within classically described 60- to 80-nm vesicles, which has led to large amount of work to define the molecular requirements for COPII-dependent collagen secretion. From this, many factors have been identified that act either on COPII or immediately downstream of it that are required for collagen secretion. Mutations in the Sec23A subunit of COPII led to craniofacial development defects attributable to aberrant collagen secretion (Boyadjiev et al., 2006). Detailed mechanistic work has shown that the F382L mutation results in a loss of efficient coupling of Sec23-Sec24 to Sec13-31 (Bi et al., 2007; Fromme et al., 2007). Our own work showed a particular requirement for the outer layer Sec13-Sec31 in procollagen export in vitro and in vivo (Townley et al., 2008). The conclusion from this work is that highly efficient assembly of COPII is essential for normal secretion of procollagen (Saito and Katada, 2015).

Over time, many other factors have been described that act in concert with the core COPII machinery to enhance its efficiency or, in many cases, provide specialized mechanisms for the incorporation of particular cargo into COPII-coated carriers (Venditti

 © 2016 The Author(s)
This is an open access article under the CC BY license (http://creativecommons.org/licenses/by/4.0/).

OPEN ACCESS
CellPress

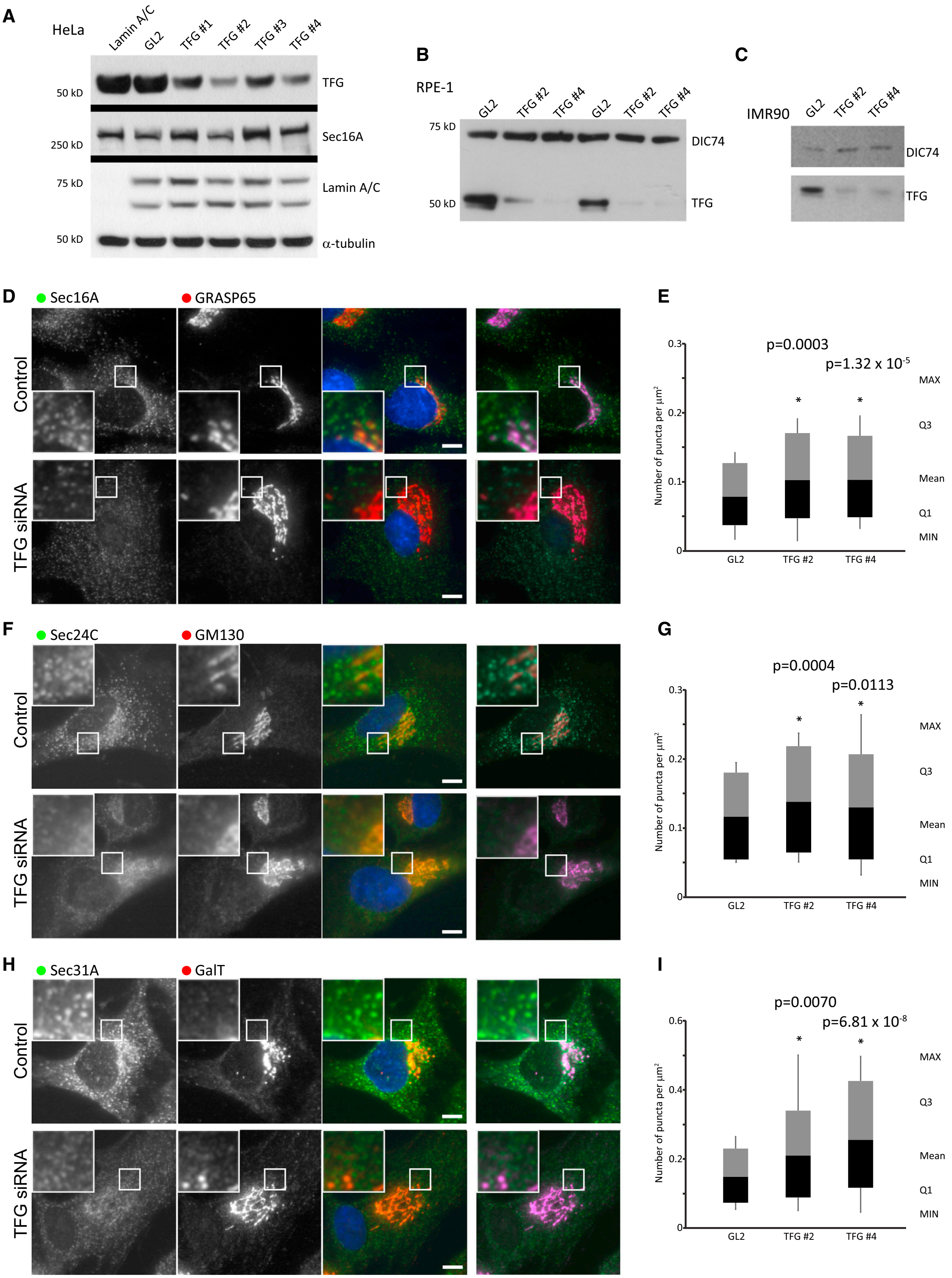

(legend on next page)

et al., 2014). The majority of such accessory factors act either by directly modulating the COPII machinery through physical interaction or through engaging cargo directly. Many of these additional components of the ER export process have been shown to be involved in the efficient export of procollagen from the ER. For example, KLHL12 is a Cullin-3 adaptor that directs mono-ubiquitylation of Sec31A, which in turn is required for efficient formation procollagen-containing carriers (Jin et al., 2012). Further downstream of COPII lies the tethering machinery that includes the TRAPPI complex. A key subunit of this complex, Sedlin, is also required for the packaging of procollagen (Venditti et al., 2012), reinforcing the concept of efficient secretory pathway activity as a prerequisite for this process. Other proteins appear to be specific for subsets of procollagen isotypes, with TANGO1 in particular being required selectively for the packaging and secretion of procollagen VII (Saito et al., 2009). The selectivity of TANGO1 for procollagen VII is debated by some. A knockout mouse for TANGO1/Mia3 shows a diversity of defects that relate to defects in the deposition of multiple collagens including I, II, and VII. In addition, cTAGE5, shown previously to facilitate the role of TANGO1 in export of procollagen VII (Saito et al., 2011), has now been shown to act in localization of Sec12 (Saito et al., 2014) and therefore presumably as a more direct component of the canonical COPII budding machinery. Whether this is due to a direct role for TANGO1/Mia3 in packaging of multiple collagen isotypes or results from defects in the assembly of a complete extracellular matrix because of a selective loss of collagen VII remains unclear. In support of a dedicated pathway for procollagen VII export, Sly1 and Syntaxin-17 were also shown to be required (Nogueira et al., 2014). This also provides further evidence of the importance of the fusion machinery in the trafficking of procollagen VII through the early secretory pathway. This work also led to the suggestion that the ERGIC could provide additional membrane to facilitate the export of atypically large cargo from the ER through a process of maturation of the nascent carrier. In support of such models, Nakano and colleagues showed that a 'hug-and-kiss' mode of direct contact of the (unstacked) *cis*-Golgi with newly forming COPII-coated carriers in the yeast *S. cerevisiae* provides a mechanism for ER-to-Golgi transport (Kurokawa et al., 2014). This process also has interesting parallels in the role of ERES and ERGIC membranes in the formation of autophagosomes at the ER membrane (Ge and Schekman, 2014; Graef et al., 2013; Wang et al., 2014). In all these cases, the ERGIC is in some way providing membrane to the newly forming carrier.

Although many tethering and fusion factors have been defined that direct the consumption of COPII-derived vesicles, it is only recently that we have gained mechanistic insight into what could maintain the structure of the ERES itself. TFG was identified as a Sec16-interacting protein that modulates COPII-dependent budding from the ER (Witte et al., 2011). TFG appears to coordinate the spatial organization of the budding event and has been proposed to act by forming oligomeric assembly that acts as a meshwork to physically connect COPII-derived vesicles and the nascent ERGIC compartment (Johnson et al., 2015). Mutations in TFG cause sensory axon degeneration, hereditary spastic paraplegia, and Charcot-Marie-Tooth disease type 2 (Beetz et al., 2013; Lee et al., 2013; Tsai et al., 2014). These defects appear to be related to structural changes in the ER (Beetz et al., 2013) that could arise from defects in protein secretion.

Here, we show that TFG directs the organization of the very earliest stages of COPII-dependent budding, the assembly of the tER. Suppression of TFG expression results in small ERES that remain functional for the export of many secretory cargoes, but not for the trafficking of procollagen. TFG therefore provides a mechanistic link between the spatial organization of the ERES and the secretion of procollagen.

RESULTS

To examine the role of TFG in the organization of mammalian ERESs and membrane traffic through the early secretory pathway, we used an RNAi approach. In line with recently published data, we found that effective depletion of TFG was possible without any overt signs of cell death. Longer-term depletion of TFG was found to cause extensive cell death. We have not been able to isolate a clone with a knockout of TFG using CRISPR (clustered regularly interspaced short palindromic repeats)-Cas9, which we suspect is due to lethality. Figure S1 shows the outcome of a transient knockout of TFG using this approach, but we have been unable to amplify cells showing this phenotype. Figure 1 demonstrates an effective knockdown of TFG expression in three human cell lines used in this study: HeLa (cervical carcinoma), RPE-1 (telomerase immortalized retinal pigment epithelial cells), and IMR-90 (fibroblasts) cells. Two of the four small interfering RNA (siRNA) duplexes used (TFG #2 and TFG #4) were consistently more effective, and these were used in all further studies. Analysis of scanned immunoblots using ImageJ showed a routine depletions were between 65% and 92% effective. Experiments showing a lower level of depletion were not included in the analysis.

Depletion of human TFG was originally shown to delay exit of tsO45-G-GFP from the ER (Witte et al., 2011). More recently, it was shown that depletion of TFG caused an increase in the number of COPII-positive structures in cells compared to tER sites (marked with Sec16A) (Johnson et al., 2015). Intriguingly, this did not appear to cause any obvious structural changes to the Golgi. Using conventional light microscopy, we observed that

Figure 1. Validation of Depletion of TFG using siRNA

(A) Immunoblotting of HeLa cell lysates from cells transfected with siRNAs targeting Lamin A/C or TFG or with a non-targeting control (GL2) as indicated. Four independent duplexes were used against TFG. Blots were probed to detect TFG, Sec16A, Lamin A/C, or α-tubulin as indicated.

(B and C) Immunoblotting of (B) RPE-1 or (C) IMR-90 cell lysates from cells transfected with siRNAs targeting GL2 or TFG as indicated showing effective depletion of TFG compared to the dynein intermediate chain subunit DIC74.

(D–I) Early secretory pathway organization in TFG-depleted cells. Widefield immunofluorescence microscopy of control or TFG-depleted cells labeled to detect (D) Sec16A and GRASP65, (F) Sec24C and GM130, and (H) Sec31A and GalT. RGB merges include DAPI labeling of nuclei. Green-magenta merges are also shown. (D), (F), and (H) show automated quantification of puncta numbers per μm^2 for (E) Sec16A, (G) Sec24C, and (I) Sec31A.

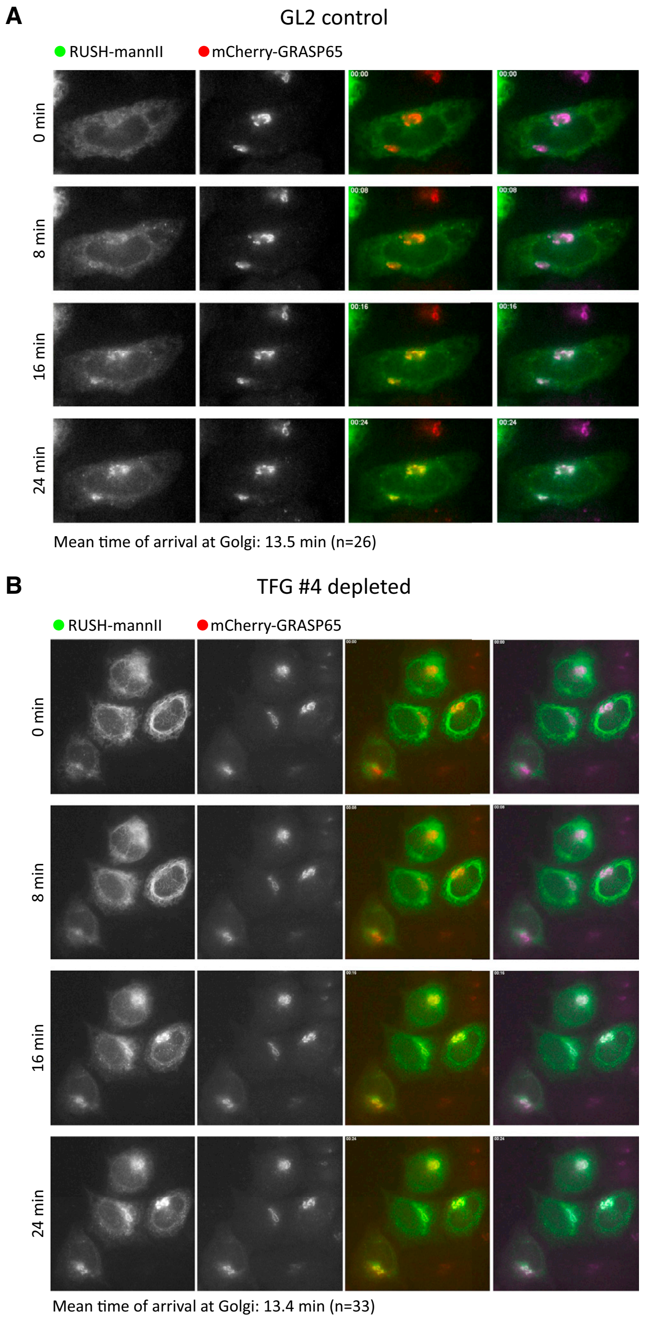

Figure 2. ER-to-Golgi Transport of Mannosidase II-GFP Is Unperturbed on TFG Depletion

(A and B) Analysis of trafficking from ER-to-Golgi of GFP-tagged mannosidase II (mannII-GFP, green) using the RUSH system. Cells stably expressing GRASP65-mCherry (red) were transfected with (A) control (GL2) or (B) TFG siRNA duplexes and subsequently with constructs to retain mannII-GFP in the ER. Addition of biotin at time = 0 releases mannII-GFP from the ER. Images are shown from time-lapse movies that are included as supplemental material (Movies S1 and S2). RGB and green-magenta merges are shown.

depletion of TFG caused not only an obvious reduction in the intensity of Sec16A labeling at tER sites but also an increase in the number of individual structures visible at this level of resolution (Figure 1D). This increase was statistically detectable using automated counting (Figure 1E). Similar data were seen for Sec24C (Figure 1F, quantified in Figure 1G) and Sec31A (Figure 1H, quantified in Figure 1I), classical markers of COPII-coated membranes. In all cases, COPII labeling was more diffuse and individual structures were less well defined, but automated quantification of the brightest 66% of objects showed the increase in number of structures for Sec16A, Sec24C, and Sec31A. This could arise either from an increase in ERES number from de novo biogenesis (Stephens, 2003) or from a disruption of the assembly of smaller ERESs into larger structures (as also described in Bevis et al., 2002).

In these datasets, we also noticed an obvious increase in the size of the Golgi when visualized using a variety of antibodies to classical markers of the Golgi (Figure 1D: Golgi reassembly and stacking protein of 65 kDa [GRASP65]; Figure 1F: Golgi matrix protein of 130 kDa [GM130]; and Figure 1H: galactosyltransferase T [GalT]). This was not manifest as a fragmentation but is more accurately described as a “loosening” of Golgi structure, reminiscent of that seen following depletion of the COPII coat component Sec13 (Townley et al., 2008). An enlarged Golgi is often associated with a moderate defect in anterograde protein trafficking from the ER to the Golgi. Indeed, depletion of TFG has been described to slow the export of cargo from the Golgi (Witte et al., 2011) and delay the reassembly of the Golgi following washout of brefeldin A (Johnson et al., 2015). Both of these assays require substantial perturbation to the cells (temperature shifts or pharmacological treatments). We examined anterograde traffic from the ER to Golgi using the RUSH (retention using selective hooks) system (Boncompain et al., 2012) (Figure 2). This system uses a streptavidin-binding peptide (SBP) fused to an ER-resident “hook” that selectively retains a GFP-tagged reporter (in our case mannosidase II) in the ER until biotin is added exogenously. We expressed a construct encoding an ER-localized hook (invariant chain SBP) and a truncated form of mannosidase II fused to EGFP (mannII-GFP) in cells depleted of TFG and measured ER-to-Golgi transport time-lapse microscopy. Arrival at the Golgi was measured by colocalization with GRASP65-mCherry, which was expressed stably in these cells (Cheng et al., 2010). We observed no detectable difference in the arrival of mannII-GFP at the Golgi in TFG-depleted cells (Figure 2B) versus controls (Figure 2A). Indistinguishable data were obtained for other secretory cargo reporters including a non-temperature-sensitive version of the VSV-G glycoprotein, E-cadherin, and GalT (not shown). The mean time of arrival of the peak of mannII-GFP at the Golgi was 14 min in both cases.

In a recent paper, depletion of TFG was reported to increase the number of COPII-positive structures in cells without increasing the number of tER sites labeled with Sec16A (Johnson et al., 2015). A knock-on effect of this was shown to be a decrease in functional coupling of tER sites to the ERGIC as monitored by the close apposition (within 500 nm) of Sec16A and ERGIC-53. Our data from conventional light microscopy (Figure 1) suggested that we did indeed see an increase in COPII-positive structures (labeled for either Sec24C or Sec31A) but in our hands this was concomitant with an increase in tER (Sec16A). ERES are of the order of 500 nm in

OPEN
ACCESS
CellPress

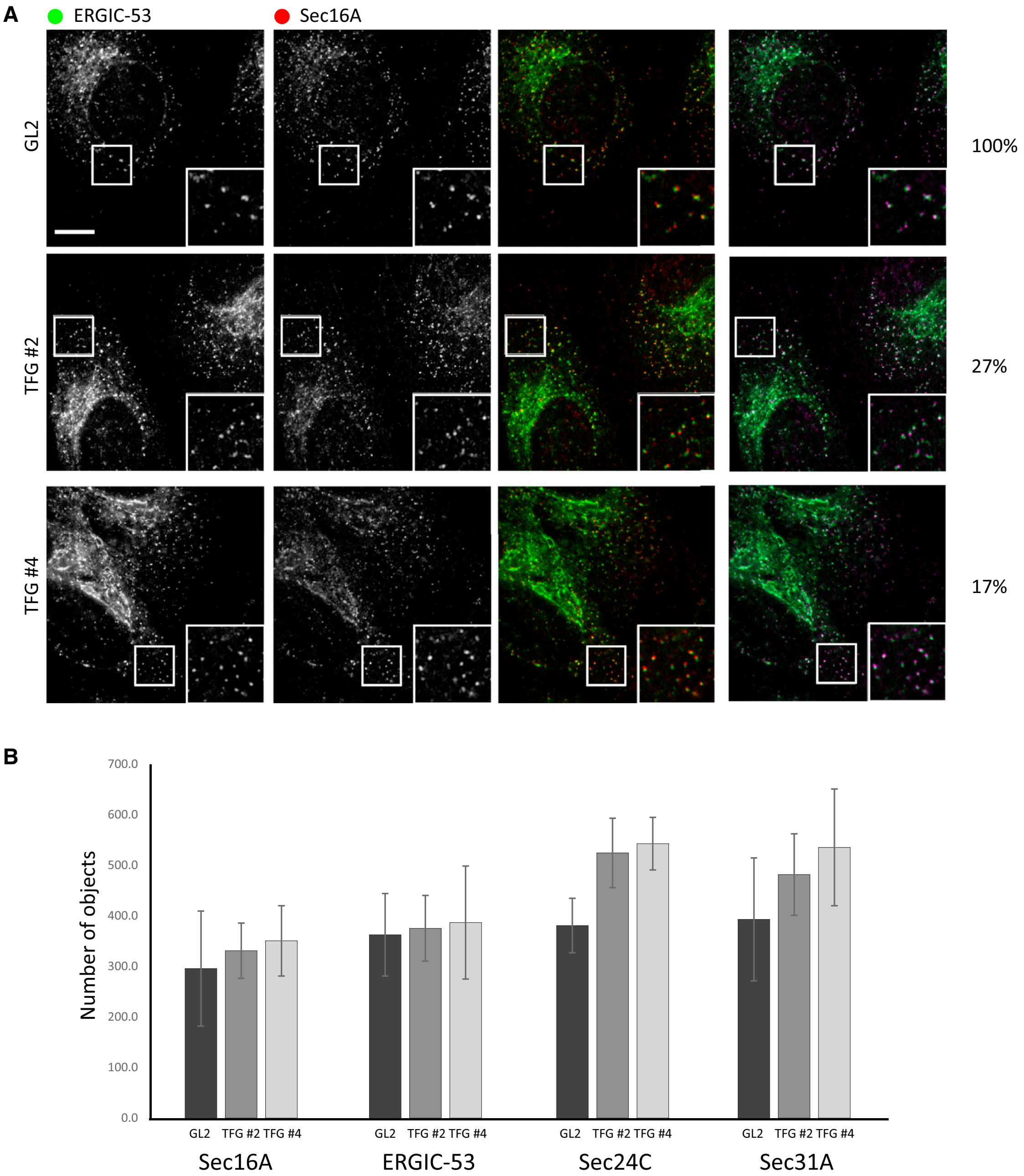

Figure 3. Super-resolution Imaging using STED of ERGIC-53 and Sec16A in Cells Depleted of TFG

Cells depleted of TFG were immunolabeled as described and imaged using STED microscopy.

(A) Images of cells include enlargements of boxed regions. Percentages indicate the approximate amount of TFG remaining in these experiments as monitored by immunoblotting.

(B) Quantification of these data using MATLAB to define the number of individual objects including analysis of Sec24C and Sec31A as in (A).

diameter, and therefore, to increase the resolution of our data beyond that of diffraction limited microscopy, we used gated stimulated emission depletion (gSTED) imaging to replicate this experiment. Using diffraction-limited fluorescent beads, we measured the full width at half maximum of our gSTED system as 90 nm (data not shown). We took tile scans of large areas of the coverslip to include multiple cells labeled for endogenous Sec16A and ERGIC-53 (Figure 3). We measured the numbers of Sec16A and ERGIC-53 positive structures and the distance between them using a custom-written MATLAB code (see Supplemental Information). In all experiments, we validated the efficiency of TFG suppression by immunoblotting (Figure S2).

Figure 3A shows the localization of Sec16A and ERGIC-53 in control cells as well as those depleted of TFG using gSTED

microscopy. Visual inspection of the data in Figure 3A suggested no obvious difference between these conditions with a possible increase in number of objects labeled with each marker following depletion of TFG. Figure 3B shows the outcome of our automated image analysis of these data, showing the number of objects identified. These data do not reveal any increase in the number of Sec16A puncta or of ERGIC-53 puncta in cells depleted of TFG. We repeated these experiments labeling for Sec24C and Sec31A. Here (Figure 3B), we do define a statistically detectable increase in the number of Sec24C structures identified and a trend toward more individual structures (albeit not statistically detectable as an increase) for Sec31A. In our widefield data, we observed an apparent increase in the number of Sec16A and ERGIC-53-positive structures. These data differ from that in Figure 1 obtained using widefield microscopy. Our interpretation here is that by imaging using the higher-resolution gSTED method, we can now identify individual ERES substructures in greater detail. Thus, using gSTED, we can identify the same number of Sec16A/ERGIC-53-positive structures, regardless of whether these individual elements are clustered into larger structures (as we see in unperturbed cells) or more dispersed (as we see following TFG depletion).

We then examined the proximity of tER to ERGIC as previous work suggested the two become decoupled on suppression of TFG (Johnson et al., 2015). Figure 4A shows enlargements of individual ERES from these cells; this illustrates the point that almost all Sec16A-positive structures have an ERGIC-53 positive structure in close apposition. In some rare cases (examples indicated by arrowheads in Figure 4A), it appears that Sec16A-positive structures exist in isolation. We then examined these more closely. Figure 4B shows that if one selects such objects from cells depleted of TFG (Figure 4B) and enhances the brightness and contrast of these images (Figure 4C), then in nearly all cases (three of four shown, arrowhead indicating a lone Sec16A puncta), one can then indeed define ERGIC-53-positive structures in close apposition.

We repeated these experiments to measure the distribution of Sec24C relative to Sec31A and of Sec16A relative to Sec31A. Our analysis defines the centroid of each object according to a 2D Gaussian function. This allowed us to measure the distance between centroids to define whether individual labels are uncoupled from one another following TFG depletion. Automated counting of individual structures was used to define those structures within 300 nm of one another. These data (Figures 5A–5C) show no statistically detectable difference in the numbers of Sec16A puncta with adjacent Sec24C (Figure 5A), Sec31A (Figure 5B), or indeed ERGIC-53 (Figure 5C) puncta following depletion of TFG. From these data, we also determined that there was no detectable change in the distance between Sec16A and ERGIC-53-positive structures in cells (Figure 5D). Our data show that depletion of TFG causes a change in ERES organization. We then used our automated analysis to measure the distance between Sec16A-positive puncta (Figure S3A). Despite a trend toward an increased separation, this was not statistically detectable as a difference (Figure S3B). This is perhaps not surprising, as an increased distance away from an original structure would result in a decrease in distance to the next closest neighbor. We do however detect a significant decrease in the size of Sec16A-positive puncta (Figure S3C) consistent with our interpretation of a dispersion of tER elements following TFG depletion. It is also possible that very small structures are below the detection limit.

To define further the effect of TFG in secretory pathway function, we then examined the trafficking of extracellular matrix proteins. Considerable previous work has demonstrated a central role for COPII function in the secretion of procollagen. Given our inability to detect any defect in canonical ER-to-Golgi traffic following depletion of TFG using the RUSH system, we took a proteomic approach to give a broader picture of the role for TFG in the secretion of extracellular matrix proteins. We used stable isotope labeling of amino acids in culture (SILAC) to compare the cell-derived matrix secreted by control and TFG-depleted cells. We considered it possible that any defects we observed here could be due to disruption of Golgi architecture as an indirect consequence of TFG depletion. Therefore, we included cells depleted of the Golgi matrix protein giantin (using a previously validated siRNA targeting giantin, Gi#1; Asante et al., 2013). Giantin is required to maintain Golgi structure but does not affect ERES organization (not evident in Koreishi et al., 2013; see Figure S4) and therefore provides an additional control. We used RPE1 cells for these experiments to complement those described above. Table S1 lists those proteins that were found in either control or giantin-depleted cell-derived matrix but that were below the level of detection following depletion of TFG (removing any proteins where only one peptide was detected). These data identify a large number of proteins associated not only with the ECM itself but also with cell adhesions. Table S2 shows a list of those proteins that were decreased in abundance >3-fold more following TFG depletion than giantin-depletion. This list is rank-ordered according to the greatest difference in abundance between TFG- and giantin-depleted cells. In addition to several unexpected proteins, including the COPII subunit Sec13 (along with other nuclear pore components that might be related to the coupling of nuclear pore complexes to adhesion complexes; Mellad et al., 2011), we observe multiple collagen subunits including those of collagens I, IV, and VI. The identification of these particular collagen isotypes likely reflects their expression in RPE-1 cells.

We then sought to validate these findings in IMR-90 human fibroblast cells, commonly used to study secretion of procollagen I. Using an antibody to the C-terminal telopeptide of collagen 1α1, we found that depletion of either giantin or TFG (Figure 6) leads to a defect in the assembly of extracellular collagen I-positive fibrils (Figure 6A). Image quantification (from experiments using two independent TFG siRNA duplexes; Figure 6B) demonstrates that this effect is significant. We included cells depleted of giantin in these experiments to further validate our proteomics and because there is clear evidence of a role of giantin in matrix assembly and collagen secretion (Katayama et al., 2011). Further analysis using widefield microscopy using an additional procollagen-specific antibody confirmed that depletion of TFG largely prevented the extracellular deposition of collagen fibrils (Figures 6C and 6D). Analysis of permeabilized cells with longer exposure times showed that procollagen accumulated inside the cells in a punctate and

OPEN
ACCESS
CellPress

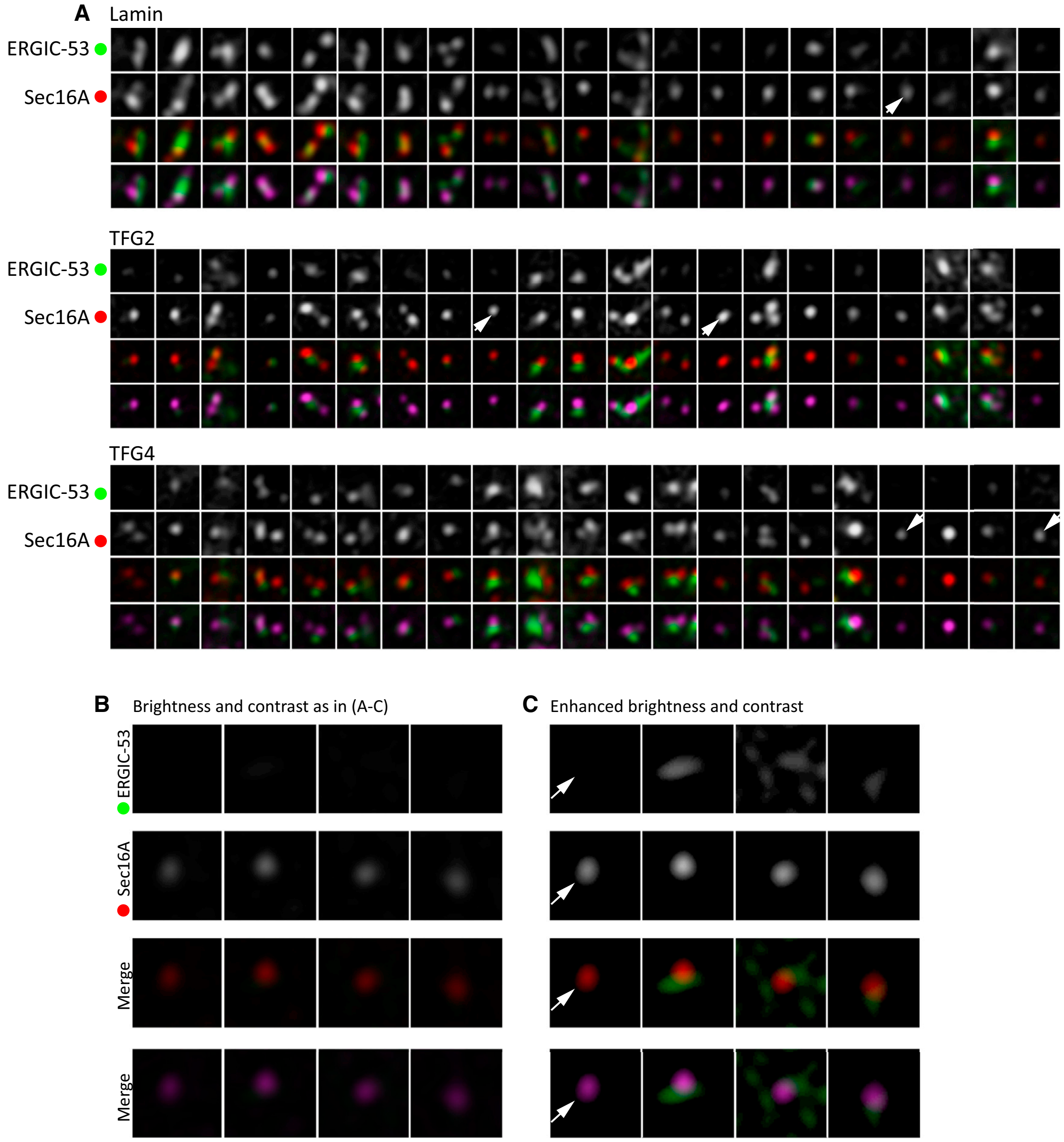

Figure 4. STED Imaging Shows Close Juxtaposition of Sec16A and ERGIC-53 Remains following TFG Depletion

(A) Enlargements of individual structures from STED imaging as in Figure 4 following TFG depletion. Arrowheads indicate cases where Sec16A appears not to have an associated ERGC-53 puncta. (B and C) Stretching brightness and contrast reveals ERGIC-53 in close proximity to Sec16A following depletion of TFG in nearly all cases; one example of a "lone" Sec16A puncta is shown (arrow).

reticular distribution consistent with an ER localization (Figure 6E). Labeling of cells for both procollagen and the ER marker calnexin demonstrates that even after the addition of ascorbate, procollagen is retained in the ER in TFG-depleted cells (Figures 6F and 6G; arrowheads indicate nuclear membrane staining which is typical of ER). We do note that not all labeling coincides perfectly, suggesting that procollagen may accumulate in a subdomain of the ER in these cells. Thus, while depletion of TFG does not appear to affect the trafficking of small soluble cargoes, these data define a clear role in the secretion and/or assembly of procollagen.

DISCUSSION

Our data suggest that TFG acts to tether or organize tER elements and identify a clear role for TFG in the secretion of type I procollagen from non-transformed human fibroblasts. We propose a model for this in Figure 7 in which TFG organizes Sec16-positive tER elements together to provide a larger, more effective platform for collagen secretion (Figure 7A). This is consistent with its identification as a Sec16-binding protein (Witte et al., 2011). The depletion of TFG results in a fragmentation of the minimal elements that make up an ERES (Figure 7B).

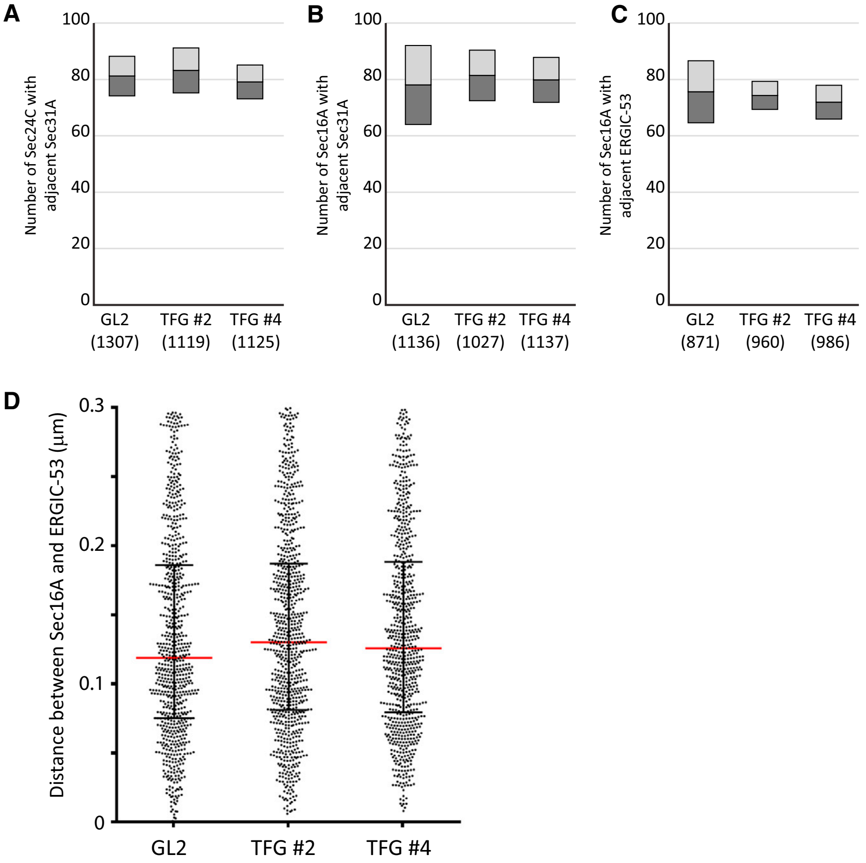

Figure 5. Automated Quantification of STED Data Show No Significant Change in Organization or Distribution of ERES Markers

(A–C) Quantification of puncta within 300 nm of one another for (A) Sec24C and Sec31A, (B) Sec16A and Sec31A, or (C) Sec16A and ERGIC-53.

(D) Measurements of the proximity of Sec16A and ERGIC-53 following depletion of TFG.

Only when in this dispersed state are these individual elements resolvable by conventional light microscopy hence why this approach gives us an apparent increase in object number. In contrast, super-resolution imaging allows us to resolve these structures (for Sec16A and ERGIC-53) in both the unperturbed and TFG-depleted states, defining the change in organization rather than object number per se here. The increase in the number of Sec24 and Sec31-coated structures that we see is in close agreement with the work of Johnson et al. (2015) and suggests that TFG serves to tether these COPII-coated post-ER structures to the underlying tER. Many studies have demonstrated an increase in ERES number with increasing cargo load (Forster et al., 2006; Guo and Linstedt, 2006). The ability to change the organization of ERES units could also be relevant under increased cargo load as a means to provide a rapid increase in ERES number. Specifically, these studies examined the ER export of small, conventional cargo. Our analysis of controlled transport assays shows no change in the rate of delivery of small soluble cargo from the ER to the Golgi following TFG depletion. Adaptation by "untethering" ERES could be beneficial for such cargoes on a more rapid timescale than biogenesis of new ERES.

Our proteomic analysis of cell-derived matrix and immunofluorescence labeling of extracellular, processed collagen demonstrates a clear defect in matrix secretion, notably that of fibrillar collagens (collagen VI in RPE-1 and collagen I in IMR-90 cells) on depletion of TFG. As discussed above, many factors have been identified that are either required for or support the ER export of one or more procollagen isotypes (Malhotra and Erlmann, 2015). The link we define here between the organization and function of ERES in collagen secretion suggests that many of these factors might act in a similar manner by optimizing the organization and/or efficiency of these sites for the assembly of large COPII cages and the transition of these cages to the ERGIC. Defects in the Sedlin component of the TRAPPI complex (Venditti et al., 2012), TANGO1 (Saito et al., 2009), cTAGE5 (Saito et al., 2011), the KLHL12 ubiquitylation machinery (Jin et al., 2012), or Sec13-Sec31 (Townley et al., 2008) have all been reported to cause defects in collagen secretion. We note that any individual defect appears sufficient to inhibit procollagen export suggesting a concerted mode of action of all such factors. Loss of function of these proteins is not necessarily linked to changes in tER or ERES organization. However, changes in the recruitment of Sec12 by cTAGE5 (Saito et al., 2014) or of the Sar1 GTPase cycle by sedlin (Venditti et al., 2012) could be lined to changes in ERES organization, although this has not been tested systematically. Loss of function of giantin also results in apparent defects in collagen secretion (Katayama et al., 2011; our data here), but we have not defined a clear role for giantin in ERES organization (Figure S4). This reinforces the fact that while loss of TFG results in a defect in both tER organization and collagen secretion, this is not necessarily a causative relationship.

Many of these components should therefore be considered, along with TFG, as key factors in a functional minimal secretion system that must act together to optimize the system for the export of atypical cargo including fibrillar procollagens. In other cases as well, defects in secretory pathway machineries appear to cause primary defects in extracellular matrix deposition, chondrogenesis, and bone formation (Boyadjiev et al., 2006; Garbes et al., 2015; Katayama et al., 2011; Lang et al., 2006; Melville et al., 2011; Smits et al., 2010).

We currently have a very detailed understanding of the mechanisms of COPII-dependent budding from the ER, much of which has come from yeast genetics, in vitro reconstitution, and biochemical experiments (Zanetti et al., 2011). This has led

OPEN ACCESS
CellPress

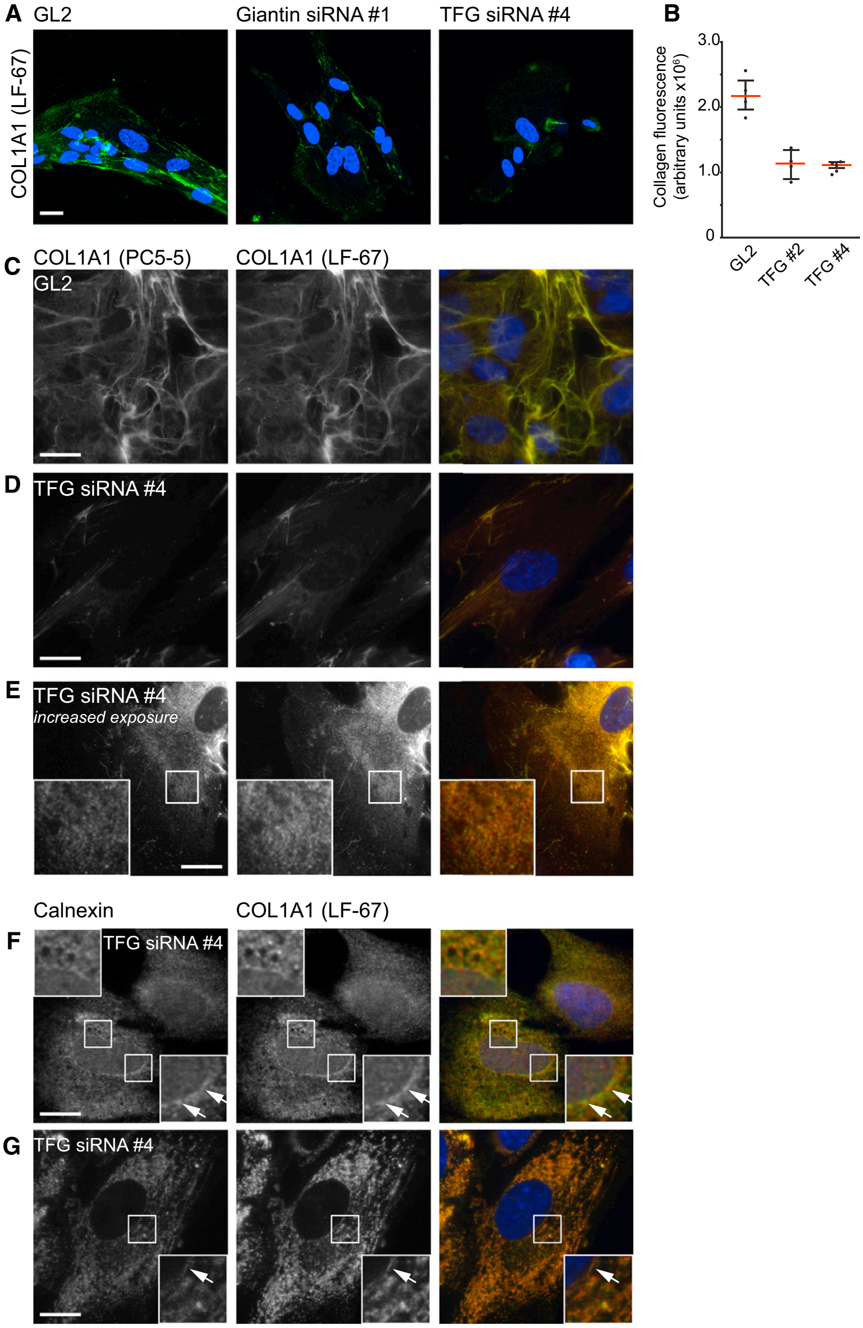

Figure 6. TFG Depletion Causes Defects in the Secretion of Procollagen

Cells were fixed, but not permeabilized, and labeled with an antibody to detect the C-terminal telopeptide of collagen type Iαl.

(A) Images of cells showing extracellular collagen fibrils.

(B) Quantification of the cellular fluorescence of extracellular procollagen.

(C–G) Intracellular localization of procollagen in RPE1 cells. Cells were depleted using (C) control or (D–F) TFG siRNA duplexes, incubated for 6 hr in cycloheximide to prevent further protein synthesis, and subsequently with ascorbate for 90 min to promote procollagen folding and trafficking. Cells were fixed using paraformaldehyde, permeabilized and labeled to detect procollagen as indicated, and imaged by widefield microscopy. (C) Extracellular collagen fibrils are evident in controls as in (A). Following depletion of TFG (D), these fibrils are largely absent. Increasing the exposure time enables visualization of intracellular collagen. (E) Accumulation in puncta and reticular structures consistent with an ER localization. (F) Procollagen colocalizes with calnexin in TFG-depleted cells even in the presence of ascorbate. Two examples are shown following incubations with ascorbate for (F) 30 min and (G) 90 min.

Scale bars represent 10 μm.

to a detailed understanding of how COPII drives the accumulation of cargo into nascent buds and the fission of transport carriers from the ER. Newly emerging data have added to the complexity of this system to explain how it is adapted for more complex situations (Malhotra and Erlmann, 2015; Miller and Schekman, 2013; Saito and Katada, 2015). Models have been proposed that posit roles for post-ER membrane, notably the ERGIC, in facilitating this process for atypical membrane remodelling steps. It is widely accepted that COPII-coated vesicles uncoat and fuse either with each other (homotypically) or with a pre-existing ERGIC (heterotypically). In yeast, high-resolution light microscopy experiments indicated that mobile Golgi elements associate with COPII-coated carriers emerging from ERESs to mediate direct, efficient transfer of secretory cargo from the ER to the Golgi (Kurokawa et al., 2014). In mammalian cells, the ERGIC might play such a role, enabling the formation of larger pre-Golgi structures at peripheral sites.

An obvious means to achieve this with high efficiency would be to physically tether the tER from where COPII vesicles bud to the ERGIC. TFG has been proposed to mediate this tethering of COPII-coated membranes to the ERGIC. TFG assembles an oligomeric mesh that surrounds COPII vesicles (Johnson et al., 2015); it was suggested that this also tethers them to the ERGIC. This model was based in part on an apparent loss of connectivity between COPII-coated ERESs and ERGIC membranes following depletion of TFG. Our data do not support this model, since we do not see any change in the juxtaposition of tER and ERGIC on depletion of TFG. Our super-resolution imaging shows that nearly all Sec16-postive tER sites sit adjacent to an ERGIC, even following depletion of TFG. No increase in the number of ERGIC-53-positive structures is seen following depletion of TFG at this higher level of resolution, nor does the average distance between these structures change.

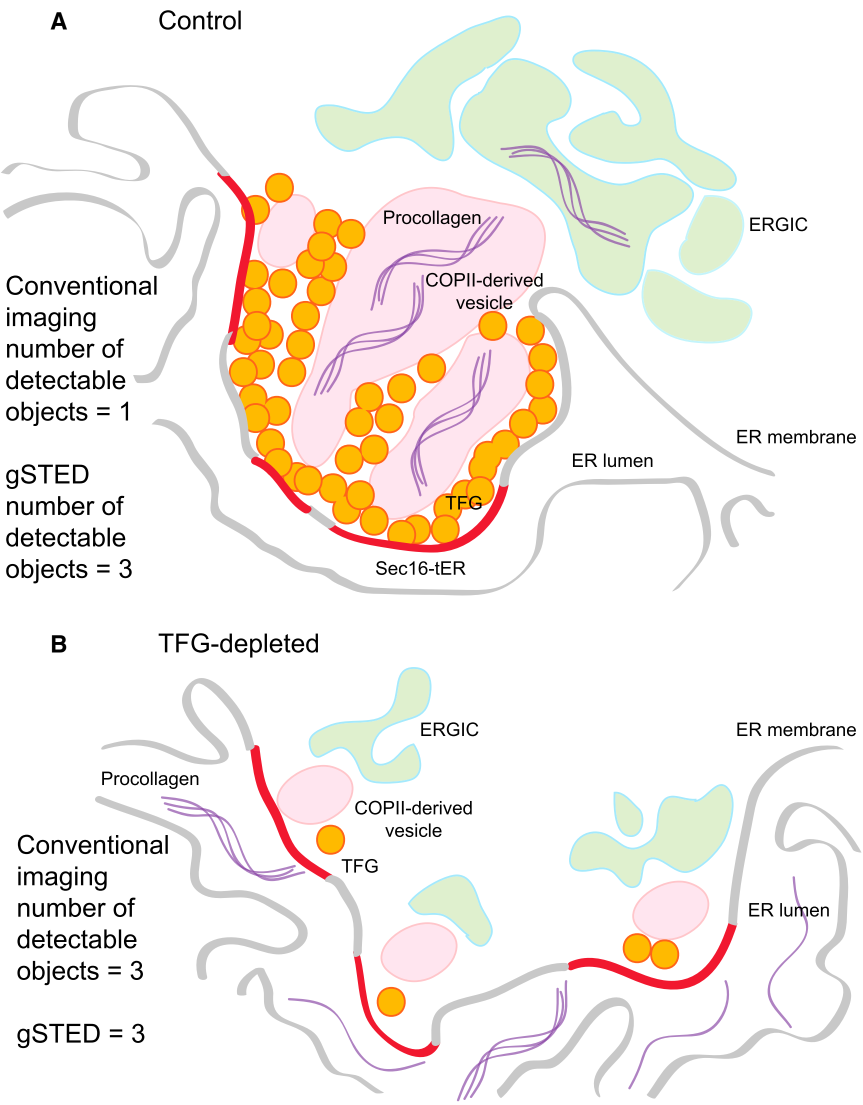

Figure 7. TFG-Dependent Higher-Order Organization of ERESs Supports Export of Procollagen from the ER

(A) Normally, TFG organizes the ERES to cluster elements of tER together. These are not resolvable by conventional light microscopy but can be discriminated using super-resolution methods. This clustering supports the formation of procollagen-containing carriers in a COPII-dependent manner. These then mature to the ERGIC.

(B) On depletion of TFG, individual tER elements become separated and detectable by conventional light microscopy and super-resolution imaging (gSTED). The proximity of ERGIC membranes to these smaller tER elements is unperturbed. Our model proposes that clustering of tER elements promotes efficient COPII assembly to support the formation of larger procollagen-containing carriers. It remains possible that tER clustering and procollagen export are not linked directly and that TFG acts in tER organization and in collagen secretion through separate mechanisms.

Combined with our extensive validation of TFG depletion, we conclude that TFG does not tether the ERGIC to the rest of the ERES. It remains possible that an even greater level of depletion of TFG than we achieved might result in a loss of tER-ERGIC coupling. However, no physical linker has been defined that could link TFG to the ERGIC. Although our data do not preclude a role for the ERGIC in providing membrane to support budding of large carriers from the ER (Santos et al., 2015), our data suggest that TFG does not mediate this directly.

Recent data implicate the ERGIC as an active player in budding from the ER, notably in what one might consider atypical ER export events. Both autophagosome biogenesis (Ge et al., 2013; Ge and Schekman, 2014; Graef et al., 2013) and procollagen export (Nogueira et al., 2014) have been shown to involve ERGIC-derived membranes. In autophagosome biogenesis, this has been defined as the site of LC3 lipidation (Ge et al., 2013). During the export of procollagen from the ER, specific SNARE proteins Sly1 and Syntaxin-18 (Nogueira et al., 2014) mediate fusion of procollagen-containing carriers from the ER with ERGIC-derived membranes to enlarge the nascent transport carrier such that it can encapsulate the atypically large cargo (Santos et al., 2015). Importantly the recruitment of ERGIC membranes during this process was found to be mediated by TANGO1, providing a physical link between the cargo (procollagen VII), the COPII coat, and the ERGIC membranes required for this step. Our data are consistent with this model but show that physical coupling of ERGIC to tER alone is not sufficient to support procollagen export in the absence of large ERESs.

In summary, we favor a model (Figure 7) in which TFG serves to organize ERES membranes, specifically the tER and COPII-coated carriers, to support procollagen export. Our data support the concept that TFG acts as a scaffold to maintain larger clusters of Sec16A and retain COPII-coated elements in close proximity to one another. In its simplest form, this model would suggest that larger platforms of Sec16 are more competent for efficient COPII assembly at the earliest stages of budding to ensure encapsulation of a nascent procollagen-containing carrier within a COPII-coated structure. Alternatively (or in addition), TFG could act to scaffold carrier expansion during emergence of procollagen from the ER or support homotypic fusion of COPII-derived vesicles to expand nascent carriers. Our data show that TFG is not required to maintain the proximity of ERGIC membranes to tER sites, and therefore, we conclude that this juxtaposition of membranes is not sufficient to support procollagen secretion. Instead, our data are consistent with models in which the higher-order organization of these membranes into a larger ERES optimizes COPII-dependent budding to facilitate procollagen packaging at the ER.

EXPERIMENTAL PROCEDURES

Widefield, Confocal, and Super-resolution Microscopy

Widefield microscopy on live cells was performed using Olympus IX81 microscope with 60× 1.42 numerical aperture (NA) oil-immersion lens, Sutter DG4 illumination with excitation filters, and multi-pass dichoric and multi-pass emission filters (Semrock). Images were collected using an Orca Flash 2.8 sCMOS controlled using Volocity 5.4.2 (PerkinElmer). Fixed cells were imaged using an Olympus IX70 microscope with 60× 1.42 NA oil-immersion lens, Exfo 120 metal halide illumination with excitation, dichroic and emission filters (Semrock), and a Photometrics Coolsnap HQ2 CCD, controlled by Volocity 5.4.1 (PerkinElmer). Chromatic shifts in images were registration corrected using TetraSpek fluorescent beads. Confocal images were obtained with Leica SP5II, and for super-resolution microscopy, a Leica Microsystems STED SPX was used. STED microscopy samples were mounted with Prolong Diamond, and Alexa-Fluor-532-and Alexa4-88-conjugated secondary antibodies were used (all from Thermo Fisher Scientific). For localization analysis, antibody combinations were imaged as two-color STED tile scans consisting of 42 tiles containing approximately ten cells in focus. The field of view was randomly picked, but if necessary, it was moved to an area with more cells in the same focal plane. For data analysis, an area of 9.65 μm^2 in the cell periphery of ten cells per sample was selected to even out the number of puncta and background noise between analyzed fields. Laser intensities were optimized for GL2 samples for each replicate. Settings among GL2, T2, and T4 samples within one set were not changed. Tile scans were performed by using the sequential scanning mode between frames, where the 488-labeled proteins were imaged first. To ensure that chromatic shift has no influence on the data analysis, four color 40-nm beads (TetraSpek, Life Technologies) were imaged with the mentioned settings and the automatic chromatic shift calculator of Volocity determined a -1 shift in y (not detectable by eye), which was adjusted for in the MATLAB code used for the data analysis.

Localization of ERES Components After TFG Depletion via Super-resolution Microscopy

With the help of a MATLAB code (see Data S1) the 9.65 μm^2 fields of view of ten cells per sample were analyzed together and the distance between the centroids of puncta from channel 0 (Ch0) and channel 1 (Ch1) was measured and the number of puncta, as well as neighbors in the other channel counted. For a distance analysis a cut off of 300 nm was chosen to prevent measurement of distances from puncta too far away from each other. This range corresponds to the estimated maximum size of large COPII vesicles (~400 nm), as the diameter of measured puncta is ~90–100 nm. Also, if a distance above 300 nm is allowed, measurements showed distances between groups of puncta, that already have a corresponding partner in the other channel.

MATLAB Code for Analysis of STED Data

Images were automatically analyzed with custom-written code (MATLAB 2014a, MathWorks). Images were first corrected for any chromatic aberrations or shifts as identified through imaging of multi-color 100-μm fluorospheres (TetraSpek, Life Technologies). For each channel, images were pre-processed using a 2D wiener with a kernel radius of 2 (denoising) and then converted to frequency space through use of a 2D Fourier transform. Data were thresholded in the frequency domain (smoothed), and after applying an inverse Fourier transform, an adaptive local intensity threshold was applied to convert the image to binary. Touching objects were then separated through use of a watershed algorithm and objects parameters were quantified (area, centroid, and intensity parameters). A k-nearest-neighbor search was performed to look for the nearest 20 neighbors within 500 nm of each ERGIC-53. A further k-nearest-neighbor search was then performed within this search area to measure the distances between Sec16A proteins. All measured distances were then outputted to a spreadsheet for further statistical analysis.

Statistical Analysis

Statistical analysis was done to estimate differences in protein localization between control and TFG-depleted samples. As the measured distances between centroids are non-parametrically distributed, statistical analysis was performed using Kruskal-Wallis test followed by Dunnett's multiple comparison test using Prism 4.03 on the grouped values of all at least three independent replicates (n = 3).

SUPPLEMENTAL INFORMATION

Supplemental Information includes Supplemental Experimental Procedures, four figures, two tables, two movies, and two data files and can be found with this article online at http://dx.doi.org/10.1016/j.celrep.2016.04.062.

AUTHOR CONTRIBUTIONS

D.J.S. conceived the project, performed experiments and data analysis, and wrote the paper. J.M. performed TFG depletion immunoblots and with D.A. performed the STED microscopy and data analysis. N.L.S. performed experiments and analyzed data relating to ERES distribution. V.J.M., J.M., and D.J.S. performed and analyzed collagen secretion experiments. A.B. and A.K.B. performed initial validation of TFG depletion and experiments in Figure 1. V.J.M. performed the CRISPR knockout experiments and all RUSH and proteomics experiments. K.J.H. helped design and perform the proteomic experiments and initial data analysis.

ACKNOWLEDGMENTS

This work was funded by the Medical Research Council UK (grant no. MR/J000604/1, MR/K018019/1, and G0801848). We thank the Wolfson Foundation and University of Bristol for funding of the Wolfson Bioimaging Facility. This work also benefited from additional equipment funded by the BBSRC (through BrisSynBio, a BBSRC/EPSRC-funded Synthetic Biology Research Centre, grant L01386X) and an ALERT 13 capital grant (BB/L014181/1). We thank Jon Audhya and the other members of the D.J.S. lab (Jon Lane, Pete Cullen, and Harry Mellor) for helpful discussions throughout the project. We thank Larry Fisher (NIH) for providing antibody LF-67, Jon Lane for GRASP65-mCherry cells, and Franck Perez and Gaelle Boncompain for sharing the RUSH system with us. We also thank the University of Bristol, Wolfson Foundation, and Wellcome Trust for funding of the University of Bristol Proteomics Facility.

Received: January 5, 2016
Revised: March 16, 2016
Accepted: April 15, 2016
Published: May 12, 2016

REFERENCES

Asante, D., Maccarthy-Morrogh, L., Townley, A.K., Weiss, M.A., Katayama, K., Palmer, K.J., Suzuki, H., Westlake, C.J., and Stephens, D.J. (2013). A role for the Golgi matrix protein giantin in ciliogenesis through control of the localization of dynein-2. J. Cell Sci. *126*, 5189–5197.

Bächinger, H.P., Doege, K.J., Petschek, J.P., Fessler, L.I., and Fessler, J.H. (1982). Structural implications from an electronmicroscopic comparison of procollagen V with procollagen I, pC-collagen I, procollagen IV, and a Drosophila procollagen. J. Biol. Chem. *257*, 14590–14592.

Bannykh, S.I., Rowe, T., and Balch, W.E. (1996). The organization of endoplasmic reticulum export complexes. J. Cell Biol. *135*, 19–35.

Beetz, C., Johnson, A., Schuh, A.L., Thakur, S., Varga, R.E., Fothergill, T., Hertel, N., Bomba-Warczak, E., Thiele, H., Nürnberg, G., et al. (2013). Inhibition of TFG function causes hereditary axon degeneration by impairing endoplasmic reticulum structure. Proc. Natl. Acad. Sci. USA *110*, 5091–5096.

Bevis, B.J., Hammond, A.T., Reinke, C.A., and Glick, B.S. (2002). De novo formation of transitional ER sites and Golgi structures in Pichia pastoris. Nat. Cell Biol. *4*, 750–756.

Bi, X., Mancias, J.D., and Goldberg, J. (2007). Insights into COPII coat nucleation from the structure of Sec23.Sar1 complexed with the active fragment of Sec31. Dev. Cell *13*, 635–645.

Boncompain, G., Divoux, S., Gareil, N., de Forges, H., Lescure, A., Latreche, L., Mercanti, V., Jollivet, F., Raposo, G., and Perez, F. (2012). Synchronization of secretory protein traffic in populations of cells. Nat. Methods *9*, 493–498.

Boyadjiev, S.A., Fromme, J.C., Ben, J., Chong, S.S., Nauta, C., Hur, D.J., Zhang, G., Hamamoto, S., Schekman, R., Ravazzola, M., et al. (2006). Cranio-lenticulo-sutural dysplasia is caused by a SEC23A mutation leading to abnormal endoplasmic-reticulum-to-Golgi trafficking. Nat. Genet. *38*, 1192–1197.

Canty, E.G., and Kadler, K.E. (2005). Procollagen trafficking, processing and fibrillogenesis. J. Cell Sci. *118*, 1341–1353.

Cheng, J.P., Betin, V.M., Weir, H., Shelmani, G.M., Moss, D.K., and Lane, J.D. (2010). Caspase cleavage of the Golgi stacking factor GRASP65 is required for Fas/CD95-mediated apoptosis. Cell Death Dis. *1*, e82.

Forster, R., Weiss, M., Zimmermann, T., Reynaud, E.G., Verissimo, F., Stephens, D.J., and Pepperkok, R. (2006). Secretory cargo regulates the turnover of COPII subunits at single ER exit sites. Curr. Biol. *16*, 173–179.

Fromme, J.C., Ravazzola, M., Hamamoto, S., Al-Balwi, M., Eyaid, W., Boyadjiev, S.A., Cosson, P., Schekman, R., and Orci, L. (2007). The genetic basis of a craniofacial disease provides insight into COPII coat assembly. Dev. Cell *13*, 623–634.

Garbes, L., Kim, K., Rieß, A., Hoyer-Kuhn, H., Beleggia, F., Bevot, A., Kim, M.J., Huh, Y.H., Kweon, H.S., Savarirayan, R., et al. (2015). Mutations in SEC24D, encoding a component of the COPII machinery, cause a syndromic form of osteogenesis imperfecta. Am. J. Hum. Genet. *96*, 432–439.

Ge, L., and Schekman, R. (2014). The ER-Golgi intermediate compartment feeds the phagophore membrane. Autophagy *10*, 170–172.

Ge, L., Melville, D., Zhang, M., and Schekman, R. (2013). The ER-Golgi intermediate compartment is a key membrane source for the LC3 lipidation step of autophagosome biogenesis. eLife *2*, e00947.

Graef, M., Friedman, J.R., Graham, C., Babu, M., and Nunnari, J. (2013). ER exit sites are physical and functional core autophagosome biogenesis components. Mol. Biol. Cell *24*, 2918–2931.

Guo, Y., and Linstedt, A.D. (2006). COPII-Golgi protein interactions regulate COPII coat assembly and Golgi size. J. Cell Biol. *174*, 53–63.

Hughes, H., Budnik, A., Schmidt, K., Palmer, K.J., Mantell, J., Noakes, C., Johnson, A., Carter, D.A., Verkade, P., Watson, P., and Stephens, D.J. (2009). Organisation of human ER-exit sites: requirements for the localisation of Sec16 to transitional ER. J. Cell Sci. *122*, 2924–2934.

Jin, L., Pahuja, K.B., Wickliffe, K.E., Gorur, A., Baumgärtel, C., Schekman, R., and Rape, M. (2012). Ubiquitin-dependent regulation of COPII coat size and function. Nature *482*, 495–500.

Johnson, A., Bhattacharya, N., Hanna, M., Pennington, J.G., Schuh, A.L., Wang, L., Otegui, M.S., Stagg, S.M., and Audhya, A. (2015). TFG clusters COPII-coated transport carriers and promotes early secretory pathway organization. EMBO J. *34*, 811–827.

Katayama, K., Sasaki, T., Goto, S., Ogasawara, K., Maru, H., Suzuki, K., and Suzuki, H. (2011). Insertional mutation in the Golgb1 gene is associated with osteochondrodysplasia and systemic edema in the OCD rat. Bone *49*, 1027–1036.

Koreishi, M., Gniadek, T.J., Yu, S., Masuda, J., Honjo, Y., and Satoh, A. (2013). The golgin tether giantin regulates the secretory pathway by controlling stack organization within Golgi apparatus. PLoS ONE *8*, e59821.

Kurokawa, K., Okamoto, M., and Nakano, A. (2014). Contact of cis-Golgi with ER exit sites executes cargo capture and delivery from the ER. Nat. Commun. *5*, 3653.

Lang, M.R., Lapierre, L.A., Frotscher, M., Goldenring, J.R., and Knapik, E.W. (2006). Secretory COPII coat component Sec23a is essential for craniofacial chondrocyte maturation. Nat. Genet. *38*, 1198–1203.

Lee, S.S., Lee, H.J., Park, J.M., Hong, Y.B., Park, K.D., Yoo, J.H., Koo, H., Jung, S.C., Park, H.S., Lee, J.H., et al. (2013). Proximal dominant hereditary motor and sensory neuropathy with proximal dominance association with mutation in the TRK-fused gene. JAMA Neurol. *70*, 607–615.

Malhotra, V., and Erlmann, P. (2011). Protein export at the ER: loading big collagens into COPII carriers. EMBO J. *30*, 3475–3480.

Malhotra, V., and Erlmann, P. (2015). The pathway of collagen secretion. Annu. Rev. Cell Dev. Biol. *31*, 109–124.

Mellad, J.A., Warren, D.T., and Shanahan, C.M. (2011). Nesprins LINC the nucleus and cytoskeleton. Curr. Opin. Cell Biol. *23*, 47–54.

Melville, D.B., Montero-Balaguer, M., Levic, D.S., Bradley, K., Smith, J.R., Hatzopoulos, A.K., and Knapik, E.W. (2011). The feelgood mutation in zebrafish dysregulates COPII-dependent secretion of select extracellular matrix proteins in skeletal morphogenesis. Dis. Model. Mech. *4*, 763–776.

Miller, E.A., and Schekman, R. (2013). COPII - a flexible vesicle formation system. Curr. Opin. Cell Biol. *25*, 420–427.

Mouw, J.K., Ou, G., and Weaver, V.M. (2014). Extracellular matrix assembly: a multiscale deconstruction. Nat. Rev. Mol. Cell Biol. *15*, 771–785.

Nogueira, C., Erlmann, P., Villeneuve, J., Santos, A.J., Martínez-Alonso, E., Martínez-Menárguez, J.A., and Malhotra, V. (2014). SLY1 and Syntaxin 18 specify a distinct pathway for procollagen VII export from the endoplasmic reticulum. eLife *3*, e02784.

Saito, K., and Katada, T. (2015). Mechanisms for exporting large-sized cargoes from the endoplasmic reticulum. Cell. Mol. Life Sci. *72*, 3709–3720.

Saito, K., Chen, M., Bard, F., Chen, S., Zhou, H., Woodley, D., Polischuk, R., Schekman, R., and Malhotra, V. (2009). TANGO1 facilitates cargo loading at endoplasmic reticulum exit sites. Cell *136*, 891–902.

Saito, K., Yamashiro, K., Ichikawa, Y., Erlmann, P., Kontani, K., Malhotra, V., and Katada, T. (2011). cTAGE5 mediates collagen secretion through interaction with TANGO1 at endoplasmic reticulum exit sites. Mol. Biol. Cell *22*, 2301–2308.

Saito, K., Yamashiro, K., Shimazu, N., Tanabe, T., Kontani, K., and Katada, T. (2014). Concentration of Sec12 at ER exit sites via interaction with cTAGE5 is required for collagen export. J. Cell Biol. *206*, 751–762.

Santos, A.J., Raote, I., Scarpa, M., Brouwers, N., and Malhotra, V. (2015). TANGO1 recruits ERGIC membranes to the endoplasmic reticulum for procollagen export. eLife *4*, e10982.

Smits, P., Bolton, A.D., Funari, V., Hong, M., Boyden, E.D., Lu, L., Manning, D.K., Dwyer, N.D., Moran, J.L., Prysak, M., et al. (2010). Lethal skeletal dysplasia in mice and humans lacking the golgin GMAP-210. N. Engl. J. Med. *362*, 206–216.

Stephens, D.J. (2003). De novo formation, fusion and fission of mammalian COPII-coated endoplasmic reticulum exit sites. EMBO Rep. *4*, 210–217.

Townley, A.K., Feng, Y., Schmidt, K., Carter, D.A., Porter, R., Verkade, P., and Stephens, D.J. (2008). Efficient coupling of Sec23-Sec24 to Sec13-Sec31 drives COPII-dependent collagen secretion and is essential for normal craniofacial development. J. Cell Sci. *121*, 3025–3034.

Tsai, P.C., Huang, Y.H., Guo, Y.C., Wu, H.T., Lin, K.P., Tsai, Y.S., Liao, Y.C., Liu, Y.T., Liu, T.T., Kao, L.S., et al. (2014). A novel TFG mutation causes Charcot-Marie-Tooth disease type 2 and impairs TFG function. Neurology *83*, 903–912.

Venditti, R., Scanu, T., Santoro, M., Di Tullio, G., Spaar, A., Gaibisso, R., Beznoussenko, G.V., Mironov, A.A., Mironov, A., Jr., Zelante, L., et al. (2012). Sedlin controls the ER export of procollagen by regulating the Sar1 cycle. Science *337*, 1668–1672.

Venditti, R., Wilson, C., and De Matteis, M.A. (2014). Exiting the ER: what we know and what we don't. Trends Cell Biol. *24*, 9–18.

Wang, J., Tan, D., Cai, Y., Reinisch, K.M., Walz, T., and Ferro-Novick, S. (2014). A requirement for ER-derived COPII vesicles in phagophore initiation. Autophagy *10*, 708–709.

Witte, K., Schuh, A.L., Hegermann, J., Sarkeshik, A., Mayers, J.R., Schwarze, K., Yates, J.R., 3rd, Eimer, S., and Audhya, A. (2011). TFG-1 function in protein secretion and oncogenesis. Nat. Cell Biol. *13*, 550–558.

Zanetti, G., Pahuja, K.B., Studer, S., Shim, S., and Schekman, R. (2011). COPII and the regulation of protein sorting in mammals. Nat. Cell Biol. *14*, 20–28.

Cell Reports, Volume *15*

Supplemental Information

TFG Promotes Organization of Transitional ER

and Efficient Collagen Secretion

Janine McCaughey, Victoria J. Miller, Nicola L. Stevenson, Anna K. Brown, Annika Budnik, Kate J. Heesom, Dominic Alibhai, and David J. Stephens

List of supplementary materials

Supplemental Experimental Procedures.

Supplementary Figure S1: Transfection of RPE1 cells with Cas9 and guide RNA to knockout TFG expression. Relates to the rationale for Figure 1.

Supplementary Figure S2: Efficacy of depletion of TFG was monitored for each STED experiment to ensure representative image data. Relates to Figure 4 and 5.

Supplementary Figure S3: Additional data in response to reviewers. Relates to multiple figures, notably 4, 5, and 6.

Supplementary Figure S4: Additional data in response to reviewers. Relates to Figures 6 and 8.

Supplementary Movie 1: Relates to Figure 3. Movie for Fig 3A.avi
ER-to-Golgi transport of mannosidase II-GFP in control (GL2 siRNA-transfected) cells.

Supplementary Movie 2: Relates to Figure 3. Movie for Fig 3B.avi
ER-to-Golgi transport of mannosidase II-GFP in TFG-depleted cells.

Supplemental Table 1: Relates to text and rationale for experiments in Figure 7.

Supplemental Table 2: Relates to text and rationale for experiments in Figure 7.

Supplemental note: Matlab code for STED image analysis. This provides an explanation of implementation of the MatLab code used in Figure 4, 5, and S3.

Analysis_of_STED_data_2015_10_29.m: The code used to analyse data in figures 4, 5, and S3 is included in this file.

Supplemental Experimental Procedures

All reagents were purchased from Sigma-Aldrich (Poole, UK) or Thermo Fisher (Paisley, UK) unless stated otherwise.

Cell culture

The hTERT-RPE-1 (human retinal pigment epithelial cells immortalised with human telomerase reverse transcriptase, (Clontech)) cells were grown in DMEM F12 supplemented with 10 % heat-inactivated FCS (Thermo Fisher, UK). HeLa cells (ATCC CCL-2) were cultured in DMEM supplemented with 10 % heat-inactivated FCS. IMR-90 cells (ATCC CCL-186) were grown in minimal essential media supplemented with 5 ml l-glutamine and 10 % heat-inactivated FCS.

Depletion in RPE-1 and HeLa cells

TFG depletion via siRNA was performed using calcium phosphate buffer. On the first day 300 µL of cell suspension were seeded into a 6-well plate and incubated overnight. On the second day cells with a confluence of about 60 – 80 % were transfected with GL2, T2 or T4 siRNA. For the transfection buffer all stated components (using either T2 or T4 anti-TFG siRNA or GL2 anti-luciferase for control samples) were mixed and incubated at RT for 20 min to enable proper complex formation. Subsequently 200 µL of transfection buffer were added drop wise with a considerate distance onto the cells while rocking the plate, to provide finer distribution of the formed granulates. Transfected cells were incubated at 37 °C and 3 % CO_2 to help formation and dispersion of the calcium phosphate precipitate (Chen and Okayama, 1987). After 15 - 16 h the media was changed to remove excessive siRNA and transfection buffer, preventing toxic effects and the cultivation was presumed at normal conditions stated above. Fixation via ice cold methanol took place after an incubation of about 72 h after transfection at a confluence of about 70 %. If cells reached confluence within this period of time, they were passaged. siRNA duplexes were synthesised by MWG Eurofins or Sigma-Aldrich). Sequences used were GL2 (targeting luciferase) 5’-CGUACGCGGAAUACUUCGAUUTT-3’; TFG #2 5’-CUUCUCAGCCUACUAAUUA-3’; TFG #4 5’-ACUUCUGAGUAAUGAUGAATT-3’. Duplexes targeting giantin are described in (Asante et al., 2013).

For localisation of ERES components a total of 8 independent transfection experiments were performed and the level of TFG depletion was determined by semi-quantitative analysis via WB and subsequent ECL. Only depletions at or above 65 % relative to control samples GL2 were considered as sufficient, labelled for proteins of interest and imaged using super resolution microscopy (STED).

Depletion in IMR-90 cells and induction of collagen secretion

For TFG depletion in IMR-90 1 ml of cell suspension was seeded in a 6 cm dish with 5 ml of media and incubated for 2 days until a confluence of about 60 % was reached. Subsequent transfection was performed using calcium phosphate buffer as mentioned before with 600 µL buffer containing 5 µL GL2, T2 or T4 siRNA. Further treatment was conducted as described previously, with the exception that cells where split 24 h before fixation into 6-well plates with small cover slips using 950 µL cell suspension in 2 ml media containing 50 $\mu g \cdot ml^{-1}$ L-ascorbate-2-phosphate to trigger collagen secretion. Therefore, the labelled collagen corresponds to 24 h after induction of collagen secretion and a total of 48 h of TFG depletion. To visualise extracellular collagen type I, TFG depleted IMR-90 were fixed with PFA without subsequent permeabilisation and stained with LF-67. Images of cells labelled with LF-67 were quantified as corrected cellular fluorescence following background subtraction.

CRISPR-Cas9

Guide RNAs used were designed using the online tool from the Broad institute. hCas9 was a gift from George Church (Addgene plasmid # 41815, (Mali et al., 2013)). The gRNAs used were taken from the work of the Church lab described in (Mali et al., 2013) and found here: http://arep.med.harvard.edu/human_crispr/. Sequences were generated by gene synthesis downstream of a U6 promoter by MWG Eurofins. Targets were on chromosome 3 as follows: chr3:100447602-100447624 (minus strand) GAAGTTCTATCAGTTCTCGACGG and chr3:100455431-100455453 (plus strand) GCTCCTGCAGAAGATCGTTCAGG.

Analysis of efficacy of depletion

Semi-quantification of TFG levels was performed using the remaining cells in the 6-wells plates after removal of 13 mm cover slips used for immunofluorescence. Cells were washed with ice cold PBS and 200 µL of lysis buffer containing protease-inhibitor cocktail against serine and cysteine proteases was added per well and plates were incubated for 15 min on ice. Cells were scraped off the dish surface and cell lysate was centrifuged at 13000 rpm and 4 °C for 10 min. The cell pellet was discarded and the supernatant was stored at -20 °C until further analysis. A BCA protein assay was performed and measured using a BioPhotometer at a wavelength of 562 nm and total protein concentrations were calculated using a calibration curve.

Immunofluorescence of ERES and Golgi

For methanol fixation, media was aspirated, cells were washed with PBS and cold (-20°C) methanol was added. The cells were fixed for 4 min at -20 °C, following repeated rinsing with PBS. For paraformaldehyde fixation, cells were washed in PBS and fixed for 20 minutes in 4% paraformaldehyde in PBS. Cells were then washed for 5 minutes in PBS containing 30 mM glycine and permeabilized using PBS containing 0.1% Triton X-100. Storage of fixed cells till further processing was performed in blocking media (3 % BSA in PBS) overnight or for 0.5 – 1 h at RT. Samples were then rinsed three times for 5 min with PBS and cells were incubated upside down in primary antibody dilutions on parafilm, to ensure even labelling, for 0.5 – 1 h at RT. Rinsing steps with PBS were repeated and incubation with secondary antibodies against rabbit, conjugated with Alexa532 or 568, or mouse, conjugated with Alexa488, (1:400 diluted in PBS-BSA) was performed as described for primary antibodies following wash with PBS.

Samples were mounted using ProLong Gold Antifade with DAPI (Thermo Fisher) or ProLong Diamond (for super resolution microscopy) mounting media and stored overnight in the dark at RT to dry. Finally, slides were stored at 4 °C in the dark till microscopic analysis.

Immunofluorescence of ERES and Golgi

For immunofluorescence, cells were grown on 0.17 mm thick cover slips (sterilised using 70 % ethanol) in 6-well plates, fixed with ice-cold methanol for 4 minutes except for procollagen detection where 4% paraformaldehyde in PBS was used and immunostained using antibodies as indicated. Antibodies used: ERGIC-53 (monoclonal clone G1/93, Alexis Biochemicals), procollagen type I (LF67, recognizing the human collagen α1 (I) carboxy-telopeptide, Larry Fisher, NIH (Bernstein et al., 1995)), procollagen type I C-peptide (clone PC5-5, QED Bioscience, San Diego, CA), giantin (polyclonal, Cambridge Bioscience, Cambridge, UK), Sec16A (KIAA0310, Bethyl Labs, Montgomery, TX), Sec24C (Stephens Lab (Townley et al., 2008)), Sec24D (Stephens Lab (Palmer et al., 2005)), Sec31A (rabbit polyclonal, Stephens Lab (Townley et al., 2008) and mouse monoclonal, Transduction Labs), calnexin

(mouse monoclonal clone (BD Transductions Labs clone 37/Calnexin)), and TFG (Novus Biologicals, Cambridge, UK).

Imaging parameters for STED microscopy

Pinhole: 1 AU; unidirectional scanning. Tile size was 1976 x 1976 pixels, pixel size of 19.62 nm, scan speed 400 Hz. For 488 nm imaging: laser power set to 10%, hybrid detector set to 457-492nm, gating 0.3-6 ns, 100.5% gain, 1x line accumulation, 2 fold frame averaging, 592nm depletion laser set to 45% power. For 532 nm imaging, laser power was 15%, hybrid detector set to 544-590 nm, gating to 1.8 – 6 ns, gain to 120%, 1x line accumulation, 2 fold frame averaging. The depletion laser (660 nm) was set to 60%.

Controlled trafficking assays using RUSH

The Retention Using Selective Hooks system (Boncompain et al., 2012) was kindly provided by Franck Perez and Gaelle Boncompain (Institut Curie, Paris, FR) and is available through Addgene. We used a plasmid (Addgene #65255) encoding invariant chain fused to SBP and the minimal targeting region of mannosaidase II fused to GFP (mannII-GFP). Both proteins are expressed from the same plasmid using an internal ribosomal entry site. Other plasmids used included the following GFP-tagged reporters VSV-glycoprotein (Addgene #65300), E-cadherin (Addgene #65288), and GalT (Addgene #65272) each within RUSH system vectors. HeLa cells stably expressing GRASP65-mCherry ((Cheng et al., 2010) a kind gift from Jon Lane, University of Bristol) were depleted with siRNA for 48 hours followed by transfection with the relevant RUSH plasmids. After a further 24 hours, cells were washed in pre-warmed imaging medium (DMEM without phenol red and supplemented with 30 nM HEPES, pH 7.4) and transferred to a live cell imaging microscope at 37°C. After equilibration for 10 mins cells were imaged by time-lapse microscopy (1 frame per minute to minimize photodamage) following the addition of biotin (40 µm final concentration).

Proteomics of cell-derived matrix

Cells were grown in SILAC medium for 6 cell division cycles prior to siRNA transfection. Amino-acids stock R0, R6 and R10 were prepared at 8.4 mg.ml^{-1} and K0, K6 and K8 at 14.6 mg.ml^{-1}. To make amino-acid stocks, 6 ml of sterile PBS for arginine or 3.5ml for lysine was added directly to the vial then filter sterilised using a 0.22 µm filter. For HeLa cells we used DMEM high glucose medium (Thermo Fisher) containing 5ml dialyzed FCS to which was added for “Light” 500 µl R0 + 500 µl K0 for “Medium” 500 µl R6 + 500 µl K4, and for “Heavy” 500 µl R10 + 500 µl K8. After siRNA transfection cells were grown for a further 72 hours. Cell derived matrix was prepared from these cells as follows. Cells were washed in PBS and extracted using pre-warmed extraction buffer (20 mM NH_4OH, 0.5% Triton X-100 in PBS; 3 ml per 6 cm plate for 2 minutes). Extraction buffer was aspirated and plates washed with PBS containing calcium and magnesium twice. Residual DNA was digested with 10 ug/ml DNase I (Roche) in PBS containing calcium and magnesium for 30 min at 37°C. Extracts were washed again twice with PBS containing calcium and magnesium and finally solubilized in 80 µl of SDS-PAGE sample buffer, resolved on precast PAGE gels (NuPAGE 4–12%; Invitrogen) and analyzed by liquid chromatography–MS/MS-based quantification as described in (Steinberg et al., 2012). Briefly, gel lanes were cut into 6 slices, each of which was digested in-gel with trypsin using an automated digestion unit (ProGest; Digilab UK) and the resulting peptides. Peptides were then fractionated using a nano-HPLC system (UltiMate 3000; DionexThermo Scientific) in 1% (vol/vol) formic acid. Peptides in 1% (vol/vol) formic acid were injected onto a C18 nanotrap column (Acclaim PepMap; Dionex, Thermo Scientific). After washing with 0.5% (vol/vol) acetonitrile, 0.1% (vol/vol) formic acid peptides were resolved on a 250 mm × 75–µm C18 reverse-phase analytical column

(Acclaim PepMap) over a 120-min organic gradient with a flow rate of 300 nl/min−1. Peptides were ionized by nano-electrospray ionization at 2.3 1 kV using a stainless steel emitter with an internal diameter of 30 μm (ES542; ProxeonThermo Scientific). Tandem MS analysis was performed on a mass spectrometer (LTQ Orbitrap Velos; Thermo Fisher Scientific). The Orbitrap was set to analyze the survey scans at 60,000 resolution, and the top six multiply charged ions in each duty cycle were selected for MS/MS in the LTQ linear ion trap. Data were acquired using the Xcalibar Xcalibur v2.1 software (Thermo Fisher Scientific). The raw data files were processed and quantified using Proteome Discoverer software v1.2 (Thermo Fisher Scientific) with searches performed against the UniProt human database by using the SEQUEST algorithm with the following criteria: Peptide precursor mass tolerance at 10ppm and MS/MS tolerance at 0.8Da peptide tolerance at 10 ppm, trypsin as the enzyme, and carboxyamidomethylation of cysteine as a fixed modification and oxidation of methionine and appropriate SILAC labels as variable modifications. The reverse database search option was enabled, and all data were filtered to satisfy a false discovery rate of <5%.

Supplemental References:

Bernstein, E.F., Fisher, L.W., Li, K., LeBaron, R.G., Tan, E.M., and Uitto, J. (1995). Differential expression of the versican and decorin genes in photoaged and sun-protected skin. Comparison by immunohistochemical and northern analyses. Lab. Invest. *72*, 662–669.

Mali, P., Yang, L., Esvelt, K.M., Aach, J., Guell, M., DiCarlo, J.E., Norville, J.E., and Church, G.M. (2013). RNA-guided human genome engineering via Cas9. Science *339*, 823–826.

Palmer, K.J., Konkel, J.E., and Stephens, D.J. (2005). PCTAIRE protein kinases interact directly with the COPII complex and modulate secretory cargo transport. J. Cell Sci. *118*, 3839–3847.

Steinberg, F., Heesom, K.J., Bass, M.D., and Cullen, P.J. (2012). SNX17 protects integrins from degradation by sorting between lysosomal and recycling pathways. J. Cell Biol. *197*, 219–230.

Legends to Supplemental Figures

Supplementary Figure S1: Transfection of RPE1 cells with Cas9 and guide RNA to knockout TFG expression. Cells were transfected with Cas9 and CRISPR guides and 72 hours later immunolabelled for giantin to identify the Golgi and TFG. The asterisk indicates an example of a cell with apparent loss of TFG expression (or at least depletion possible through one allele being hit) and concomitant disorganization of the Golgi as one might expect from a defect in ER export. Labelling of TFG is diffuse and likely non-specific in cells in which there is a sever disruption to Golgi structure. Despite repeated attempts, we were never able to clone out these cells.

Supplementary Figure S2: Efficacy of depletion of TFG was monitored for each STED experiment to ensure representative image data. The "loosening" of the Golgi was also used as a proxy for efficacy.

Supplementary Figure S3: (A) Images from the STED data showing the variation in distances between Sec16A puncta. This variability confounds the analysis of proximity. While the distance to the original nearest neighbour is increased, that object will lie closer to another (different) Sec16A-positive puncta. (B) The distance between Sec16A puncta is not statistically detectable as a significant difference. While there is a trend towards more separated objects, our automated quantification does not detect a significant difference on TFG depletion. (C) Analysis of the size of Sec16A-positive puncta shows a statistically detectable decrease in puncta size following depletion of TFG. The analysis is complicated here by the fact that brighter objects would appear larger. Data are pooled from 3 independent experiments, >600 puncta measured in each case. Asterisks indicate statistical significance.

Supplementary Figure S4: (A, B) Widefield images of control cells and cells depleted of giantin by (A) siRNA or (B) shRNA and labelled for giantin and Sec31A as indicated. Equivalent images were acquired with the same settings. (C, D) Quantification of >100 cells in each case shows no statistically detectable difference between control and giantin-depleted cells in terms of the number of individual puncta for (C) Sec24C or (D) Sec31A. Bars = 10 µm.

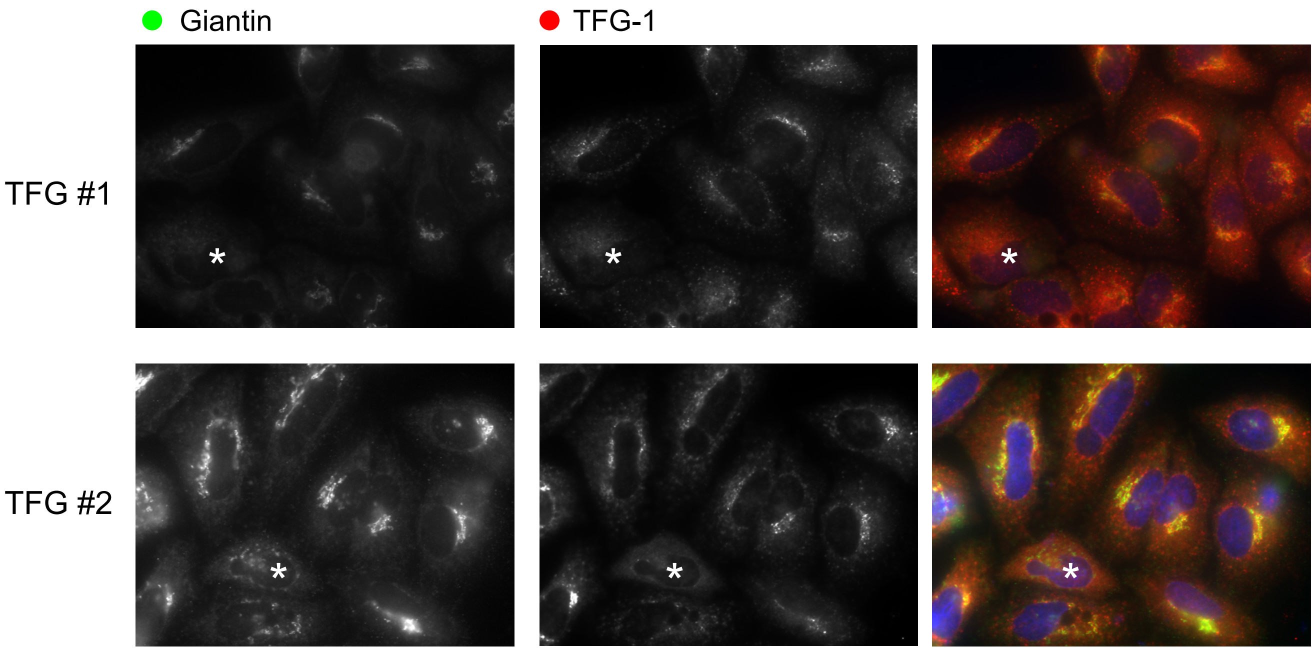
Giantin
TFG-1
TFG #1
TFG #2
*

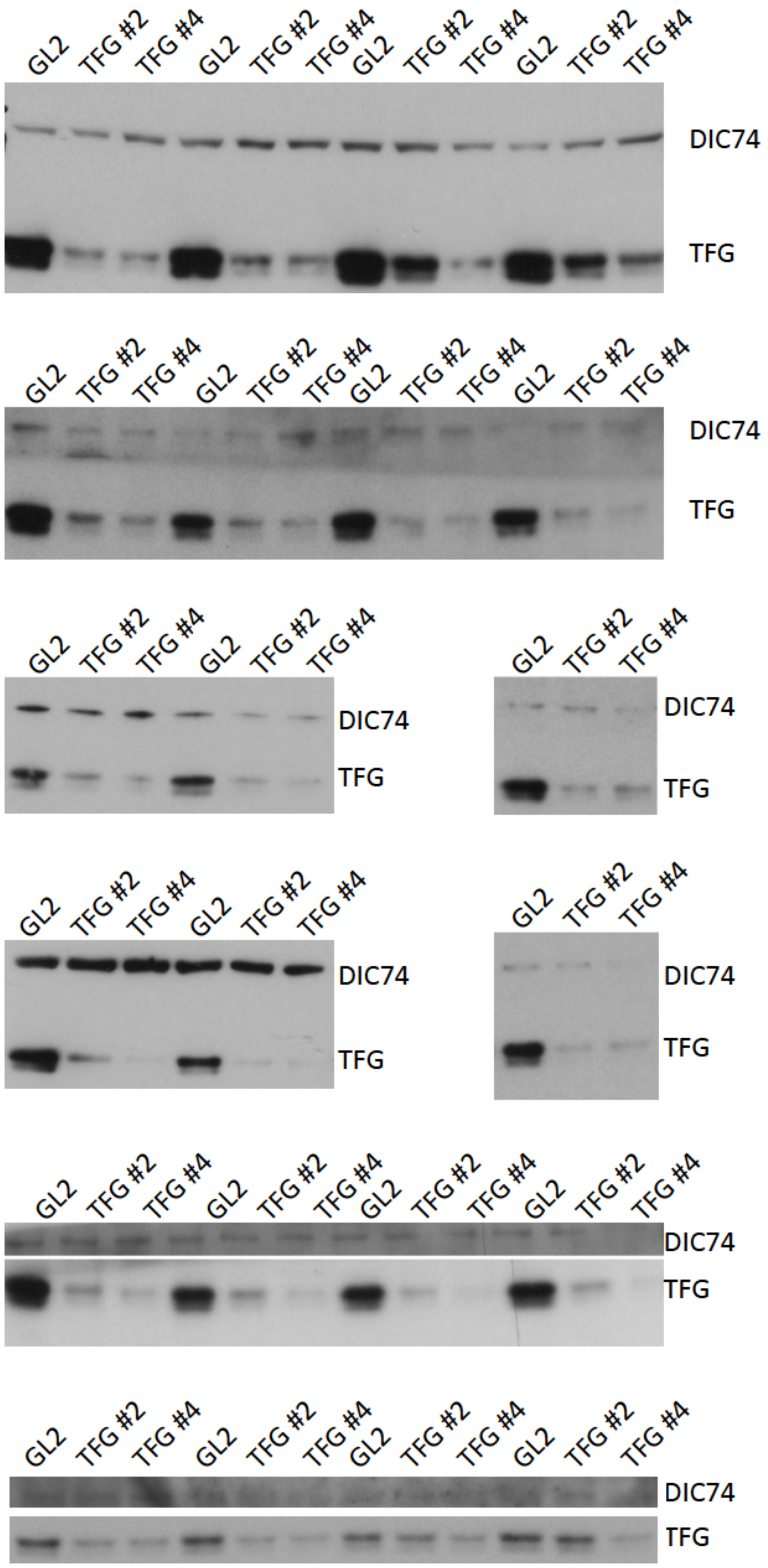
GL2
TFG #2
TFG #4
DIC74
TFG

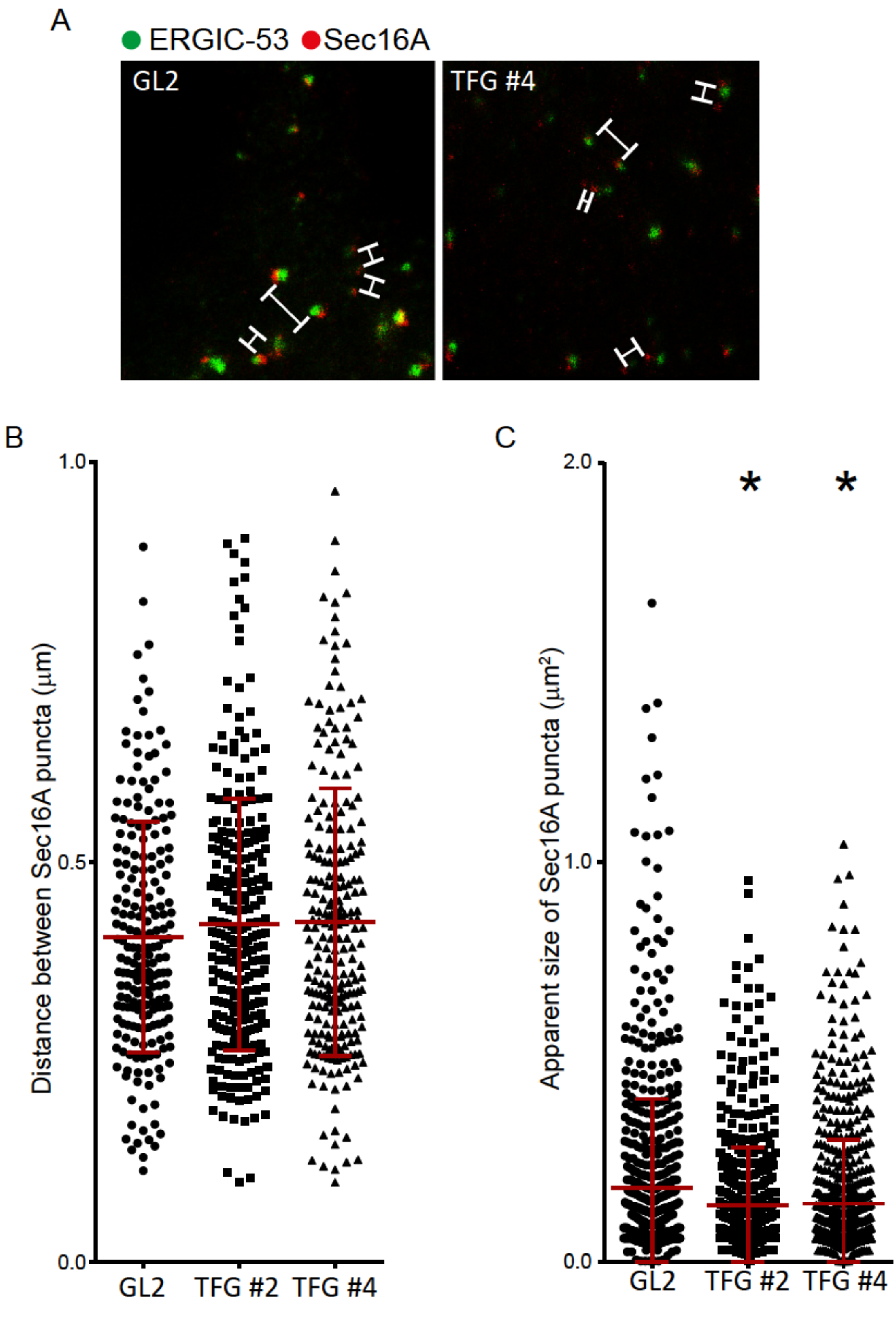
A
ERGIC-53
Sec16A
GL2
TFG #4
B
Distance between Sec16A puncta (μm)
1.0
0.5
0.0
GL2
TFG #2
TFG #4
C
Apparent size of Sec16A puncta (μm²)
2.0
1.0
0.0
*
*
GL2
TFG #2
TFG #4

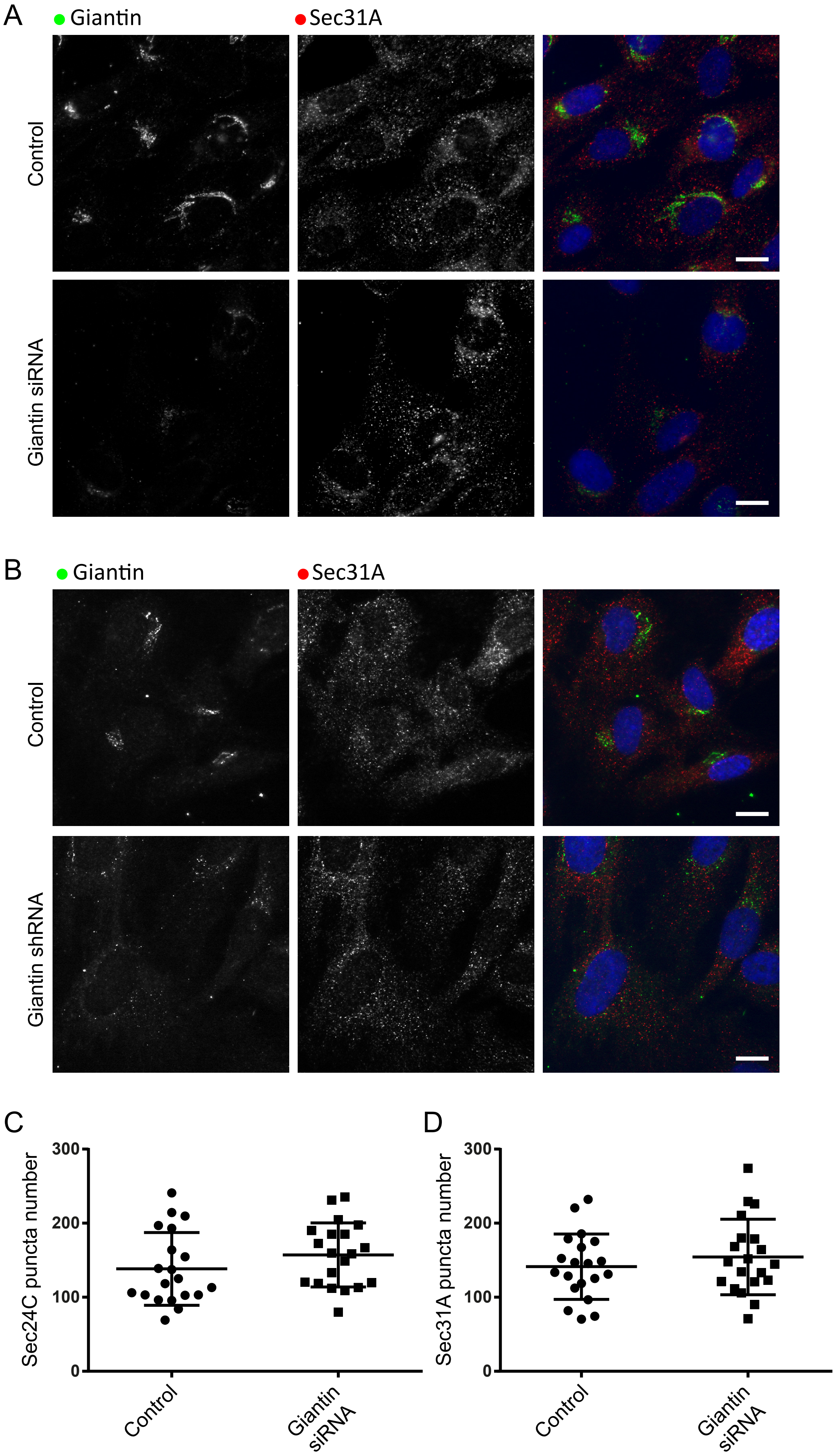
A
Giantin
Sec31A
Control
Giantin siRNA
B
Giantin
Sec31A
Control
Giantin shRNA
C
Sec24C puncta number
300
200
100
0
Control
Giantin siRNA
D
Sec31A puncta number
300
200
100
0
Control
Giantin siRNA

Legends to Supplemental Tables

Supplemental Table 1: List of proteomic hits from SILAC experiment showing those proteins identified in controls that were below the level of detection in both TFG-depleted and giantin-depleted samples. Highlighted in yellow are multiple key adhesion or matrix components. This identifies those proteins whose secretion and/or assembly into ECM is most affected by either TFG or giantin depletion. Data were filtered to remove proteins identified from a single peptide.

Supplemental Table 2: List of proteins that were decreased >3-fold more in TFG-depleted samples compared to giantin-depleted samples. This identifies those proteins who secretion and/or assembly in ECM depends more on TFG (tER organization) than giantin (Golgi organization). Highlighted in yellow are fibrillar collagens VI and I as well as collagen IV and other known collagen-binding proteins. Sec13 does appears high up on this list but we believe that this is related to its role in nuclear pore complexes for which see other components in this data set as well as other cell-derived matrix proteomes we have analysed.

Supplemental Table 1

Proteins that were detected in control but were below the level of detection following depletion of TFG.

ID	Description
TSP1_HUMAN	Thrombospondin-1 GN=THBS1 PE=1 SV=2 - [TSP1_HUMAN]
HSP7C_HUMAN	Heat shock cognate 71 kDa protein GN=HSPA8 PE=1 SV=1 - [HSP7C_HUMAN]
Q53GG0_HUMAN	Epithelial protein lost in neoplasm beta variant (Fragment) PE=2 SV=1 - [Q53GG0_HUMAN]
Q5TCU6_HUMAN	Talin 1 GN=TLN1 PE=2 SV=1 - [Q5TCU6_HUMAN]
K22E_HUMAN	Keratin, type II cytoskeletal 2 epidermal GN=KRT2 PE=1 SV=2 - [K22E_HUMAN]
K1C9_HUMAN	Keratin, type I cytoskeletal 9 GN=KRT9 PE=1 SV=3 - [K1C9_HUMAN]
K1C10_HUMAN	Keratin, type I cytoskeletal 10 GN=KRT10 PE=1 SV=6 - [K1C10_HUMAN]
B7Z2X4_HUMAN	cDNA FLJ53327, highly similar to Gelsolin PE=2 SV=1 - [B7Z2X4_HUMAN]
K1C16_HUMAN	Keratin, type I cytoskeletal 16 GN=KRT16 PE=1 SV=4 - [K1C16_HUMAN]
PLAK_HUMAN	Junction plakoglobin GN=JUP PE=1 SV=3 - [PLAK_HUMAN]
F6KPG5_HUMAN	Albumin (Fragment) PE=2 SV=1 - [F6KPG5_HUMAN]
I1VZV6_HUMAN	Hemoglobin alpha 1 GN=HBA1 PE=3 SV=1 - [I1VZV6_HUMAN]
DESP_HUMAN	Desmoplakin GN=DSP PE=1 SV=3 - [DESP_HUMAN]
DSG1_HUMAN	Desmoglein-1 GN=DSG1 PE=1 SV=2 - [DSG1_HUMAN]
F8WD96_HUMAN	Cathepsin D light chain GN=CTSD PE=3 SV=1 - [F8WD96_HUMAN]
J9ZVQ3_HUMAN	Apolipoprotein E (Fragment) GN=APOE PE=4 SV=1 - [J9ZVQ3_HUMAN]
B3KR41_HUMAN	Mitotic spindle assembly checkpoint protein MAD1 GN=MAD1L1 PE=2 SV=1 - [B3KR41_HUMAN]
K1C14_HUMAN	Keratin, type I cytoskeletal 14 GN=KRT14 PE=1 SV=4 - [K1C14_HUMAN]
K2C78_HUMAN	Keratin, type II cytoskeletal 78 GN=KRT78 PE=2 SV=2 - [K2C78_HUMAN]
TGM3_HUMAN	Protein-glutamine gamma-glutamyltransferase E GN=TGM3 PE=1 SV=4 - [TGM3_HUMAN]
Q5T985_HUMAN	Inter-alpha (Globulin) inhibitor H2 GN=ITIH2 PE=2 SV=1 - [Q5T985_HUMAN]
Q5JQ13_HUMAN	Vinculin (Fragment) GN=VCL PE=2 SV=1 - [Q5JQ13_HUMAN]
Q9NVF8_HUMAN	cDNA FLJ10762, moderately similar to CHLORINE CHANNEL PROTEIN P64 PE=2 SV=1 - [Q9NVF8_HUMAN]
Q0IIN1_HUMAN	Keratin 77 GN=KRT77 PE=2 SV=1 - [Q0IIN1_HUMAN]
FA5_HUMAN	Coagulation factor V GN=F5 PE=1 SV=4 - [FA5_HUMAN]
E9PIT3_HUMAN	Thrombin light chain GN=F2 PE=3 SV=1 - [E9PIT3_HUMAN]
LDHB_HUMAN	L-lactate dehydrogenase B chain GN=LDHB PE=1 SV=2 - [LDHB_HUMAN]
B4E054_HUMAN	cDNA FLJ58444, highly similar to Vacuolar ATP synthase subunit H PE=2 SV=1 - [B4E054_HUMAN]
B4DRW1_HUMAN	cDNA FLJ55805, highly similar to Keratin, type II cytoskeletal 4 PE=2 SV=1 - [B4DRW1_HUMAN]
Q9HB00_HUMAN	Desmocollin 1, isoform CRA_b GN=DSC1 PE=2 SV=1 - [Q9HB00_HUMAN]
S10A8_HUMAN	Protein S100-A8 GN=S100A8 PE=1 SV=1 - [S10A8_HUMAN]
Q1RMG2_HUMAN	Adenosylhomocysteinase GN=AHCY PE=2 SV=1 - [Q1RMG2_HUMAN]
PRDX1_HUMAN	Peroxiredoxin-1 GN=PRDX1 PE=1 SV=1 - [PRDX1_HUMAN]
E9PG15_HUMAN	14-3-3 protein theta (Fragment) GN=YWHAQ PE=4 SV=1 - [E9PG15_HUMAN]
I0B0K8_HUMAN	Truncated profilaggrin GN=FLG PE=3 SV=1 - [I0B0K8_HUMAN]
LPP3_HUMAN	Lipid phosphate phosphohydrolase 3 GN=PPAP2B PE=1 SV=1 - [LPP3_HUMAN]
LEG7_HUMAN	Galectin-7 GN=LGALS7 PE=1 SV=2 - [LEG7_HUMAN]
Q5HY40_HUMAN	Deoxyribonuclease-1-like 1 (Fragment) GN=DNASE1L1 PE=2 SV=1 - [Q5HY40_HUMAN]
F2Z393_HUMAN	Transaldolase GN=TALDO1 PE=3 SV=1 - [F2Z393_HUMAN]
K2C5_HUMAN	Keratin, type II cytoskeletal 5 GN=KRT5 PE=1 SV=3 - [K2C5_HUMAN]
K1C17_HUMAN	Keratin, type I cytoskeletal 17 GN=KRT17 PE=1 SV=2 - [K1C17_HUMAN]
CATA_HUMAN	Catalase GN=CAT PE=1 SV=3 - [CATA_HUMAN]
FILA2_HUMAN	Filaggrin-2 GN=FLG2 PE=1 SV=1 - [FILA2_HUMAN]
SPTB1_HUMAN	Spectrin beta chain, erythrocytic GN=SPTB PE=1 SV=5 - [SPTB1_HUMAN]
SPB12_HUMAN	Serpin B12 GN=SERPINB12 PE=1 SV=1 - [SPB12_HUMAN]
EHD3_HUMAN	EH domain-containing protein 3 GN=EHD3 PE=1 SV=2 - [EHD3_HUMAN]

FETUA_HUMAN	Alpha-2-HS-glycoprotein GN=AHSG PE=1 SV=1 - [FETUA_HUMAN]
HUTH_HUMAN	Histidine ammonia-lyase GN=HAL PE=1 SV=1 - [HUTH_HUMAN]
B4DF70_HUMAN	cDNA FLJ60461, highly similar to Peroxiredoxin-2 (EC 1.11.1.15) PE=2 SV=1 - [B4DF70_HUMAN]
F5H308_HUMAN	L-lactate dehydrogenase GN=LDHA PE=3 SV=1 - [F5H308_HUMAN]
Q8NBH6_HUMAN	cDNA PSEC0266 fis, clone NT2RP3003649, highly similar to Homo sapiens fibulin-1D mRNA PE=2 SV=1 - [Q8NBH6_HUMAN]
ARGI1_HUMAN	Arginase-1 GN=ARG1 PE=1 SV=2 - [ARGI1_HUMAN]
PSA3_HUMAN	Proteasome subunit alpha type-3 GN=PSMA3 PE=1 SV=2 - [PSA3_HUMAN]
APOC3_HUMAN	Apolipoprotein C-III GN=APOC3 PE=1 SV=1 - [APOC3_HUMAN]
B4E147_HUMAN	cDNA FLJ50342, highly similar to Myosin-5A (Fragment) PE=2 SV=1 - [B4E147_HUMAN]

Supplemental Table 2

Proteins that decreased >3-fold more following depletion of TFG than depletion of giantin.

Ranked according to the most affected first.

Gene name	Description
Q5U0B9_HUMAN	Stem cell growth factor; lymphocyte secreted C-type lectin GN=CLEC11A PE=2 SV=1 - [Q5U0B9_HUMAN]
A8MV37_HUMAN	Protein SEC13 homolog GN=SEC13 PE=4 SV=1 - [A8MV37_HUMAN]
HSPB1_HUMAN	Heat shock protein beta-1 GN=HSPB1 PE=1 SV=2 - [HSPB1_HUMAN]
B7Z641_HUMAN	V-type proton ATPase 116 kDa subunit a isoform 1 GN=ATP6V0A1 PE=2 SV=1 - [B7Z641_HUMAN]
B4DNM8_HUMAN	cDNA FLJ53395, highly similar to Prolyl 3-hydroxylase 1 (EC 1.14.11.7) PE=2 SV=1 - [B4DNM8_HUMAN]
B7Z381_HUMAN	cDNA FLJ55066, highly similar to LAS1-like protein PE=2 SV=1 - [B7Z381_HUMAN]
NOL6_HUMAN	Nucleolar protein 6 GN=NOL6 PE=1 SV=2 - [NOL6_HUMAN]
SYNEM_HUMAN	Synemin GN=SYNM PE=1 SV=2 - [SYNEM_HUMAN]
Q4W4Y1_HUMAN	Dopamine receptor interacting protein 4 GN=DRIP4 PE=2 SV=1 - [Q4W4Y1_HUMAN]
E7ESV4_HUMAN	Ras-related protein Rap-1b (Fragment) GN=RAP1B PE=4 SV=1 - [E7ESV4_HUMAN]
RAN_HUMAN	GTP-binding nuclear protein Ran GN=RAN PE=1 SV=3 - [RAN_HUMAN]
B4DUI5_HUMAN	Triosephosphate isomerase PE=2 SV=1 - [B4DUI5_HUMAN]
Q6PJT4_HUMAN	MSN protein (Fragment) GN=MSN PE=2 SV=1 - [Q6PJT4_HUMAN]
H3BTN5_HUMAN	Pyruvate kinase (Fragment) GN=PKM PE=3 SV=1 - [H3BTN5_HUMAN]
B2RDI5_HUMAN	cDNA, FLJ96627, highly similar to Homo sapiens calpain 1, (mu/I) large subunit (CAPN1), mRNA PE=2 SV=1 - [B2RDI5_HUMAN]
B4DN87_HUMAN	cDNA FLJ52569, highly similar to Collagen-binding protein 2 PE=2 SV=1 - [B4DN87_HUMAN]
RAB8A_HUMAN	Ras-related protein Rab-8A GN=RAB8A PE=1 SV=1 - [RAB8A_HUMAN]
CO6A1_HUMAN	Collagen alpha-1(VI) chain GN=COL6A1 PE=1 SV=3 - [CO6A1_HUMAN]
CO6A2_HUMAN	Collagen alpha-2(VI) chain GN=COL6A2 PE=1 SV=4 - [CO6A2_HUMAN]
B3KVN0_HUMAN	cDNA FLJ16785 fis, clone NT2RI2015342, highly similar to Solute carrier family 2, facilitated glucose transporter member 1 PE=2 SV=1 - [B3KVN0_HUMAN]
B4DT13_HUMAN	Ras-related protein Rab-11A GN=RAB11A PE=2 SV=1 - [B4DT13_HUMAN]
CO6A3_HUMAN	Collagen alpha-3(VI) chain GN=COL6A3 PE=1 SV=5 - [CO6A3_HUMAN]
TIMP3_HUMAN	Metalloproteinase inhibitor 3 GN=TIMP3 PE=1 SV=2 - [TIMP3_HUMAN]
LAMA5_HUMAN	Laminin subunit alpha-5 GN=LAMA5 PE=1 SV=8 - [LAMA5_HUMAN]
I6L965_HUMAN	KRT18 protein (Fragment) GN=KRT18 PE=2 SV=1 - [I6L965_HUMAN]
Q59GA0_HUMAN	Thy-1 cell surface antigen variant (Fragment) PE=2 SV=1 - [Q59GA0_HUMAN]
ARF6_HUMAN	ADP-ribosylation factor 6 GN=ARF6 PE=1 SV=2 - [ARF6_HUMAN]
Q59F15_HUMAN	COL4A1 protein variant (Fragment) PE=2 SV=1 - [Q59F15_HUMAN]
CO4A2_HUMAN	Collagen alpha-2(IV) chain GN=COL4A2 PE=1 SV=4 - [CO4A2_HUMAN]
NEXN_HUMAN	Nexilin GN=NEXN PE=1 SV=1 - [NEXN_HUMAN]
E9PCY7_HUMAN	Heterogeneous nuclear ribonucleoprotein H GN=HNRNPH1 PE=4 SV=1 - [E9PCY7_HUMAN]
ANXA2_HUMAN	Annexin A2 GN=ANXA2 PE=1 SV=2 - [ANXA2_HUMAN]
CO1A2_HUMAN	Collagen alpha-2(I) chain GN=COL1A2 PE=1 SV=7 - [CO1A2_HUMAN]
K7EM73_HUMAN	Calpain small subunit 1 (Fragment) GN=CAPNS1 PE=4 SV=1 - [K7EM73_HUMAN]
B4DM82_HUMAN	cDNA FLJ53060, moderately similar to Peptidyl-prolyl cis-trans isomerase A (EC 5.2.1.8) PE=2 SV=1 - [B4DM82_HUMAN]
DKC1_HUMAN	H/ACA ribonucleoprotein complex subunit 4 GN=DKC1 PE=1 SV=3 - [DKC1_HUMAN]
Q65ZQ3_HUMAN	FBRNP GN=D10S102 PE=2 SV=1 - [Q65ZQ3_HUMAN]
D6R904_HUMAN	Tropomyosin alpha-3 chain GN=TPM3 PE=4 SV=1 - [D6R904_HUMAN]
B4E3T7_HUMAN	cDNA FLJ55448, highly similar to Nuclear pore complex protein Nup133 PE=2 SV=1 - [B4E3T7_HUMAN]

DDX21_HUMAN	Nucleolar RNA helicase 2 GN=DDX21 PE=1 SV=5 - [DDX21_HUMAN]
CLH1_HUMAN	Clathrin heavy chain 1 GN=CLTC PE=1 SV=5 - [CLH1_HUMAN]
A8K7F6_HUMAN	cDNA FLJ78244, highly similar to Homo sapiens eukaryotic translation initiation factor 4A, isoform 1 (EIF4A1), mRNA PE=2 SV=1 - [A8K7F6_HUMAN]
B2R6X5_HUMAN	cDNA, FLJ93166, highly similar to Homo sapiens heat shock 70kDa protein 6 (HSP70B') (HSPA6), mRNA PE=2 SV=1 - [B2R6X5_HUMAN]
LMNB1_HUMAN	Lamin-B1 GN=LMNB1 PE=1 SV=2 - [LMNB1_HUMAN]
E9PDF6_HUMAN	Unconventional myosin-Ib GN=MYO1B PE=4 SV=1 - [E9PDF6_HUMAN]
A4QPB0_HUMAN	IQ motif containing GTPase activating protein 1 GN=IQGAP1 PE=2 SV=1 - [A4QPB0_HUMAN]
CATK_HUMAN	Cathepsin K GN=CTSK PE=1 SV=1 - [CATK_HUMAN]
PGBM_HUMAN	Basement membrane-specific heparan sulfate proteoglycan core protein GN=HSPG2 PE=1 SV=4 - [PGBM_HUMAN]
B1AK87_HUMAN	Capping protein (Actin filament) muscle Z-line, beta GN=CAPZB PE=4 SV=1 - [B1AK87_HUMAN]
GLE1_HUMAN	Nucleoporin GLE1 GN=GLE1 PE=1 SV=2 - [GLE1_HUMAN]
LMNA_HUMAN	Prelamin-A/C GN=LMNA PE=1 SV=1 - [LMNA_HUMAN]
ARC1B_HUMAN	Actin-related protein 2/3 complex subunit 1B GN=ARPC1B PE=1 SV=3 - [ARC1B_HUMAN]
NOP2_HUMAN	Putative ribosomal RNA methyltransferase NOP2 GN=NOP2 PE=1 SV=2 - [NOP2_HUMAN]
ARPC4_HUMAN	Actin-related protein 2/3 complex subunit 4 GN=ARPC4 PE=1 SV=3 - [ARPC4_HUMAN]
Q96BS4_HUMAN	FBL protein (Fragment) GN=FBL PE=2 SV=2 - [Q96BS4_HUMAN]
Q14784_HUMAN	Myosin (Fragment) PE=2 SV=1 - [Q14784_HUMAN]
ARP3_HUMAN	Actin-related protein 3 GN=ACTR3 PE=1 SV=3 - [ARP3_HUMAN]
A8K0T9_HUMAN	cDNA FLJ75422, highly similar to Homo sapiens capping protein (actin filament) muscle Z-line, alpha 1, mRNA PE=2 SV=1 - [A8K0T9_HUMAN]
B7ZAF0_HUMAN	cDNA, FLJ79164, highly similar to Tubulin beta-7 chain PE=2 SV=1 - [B7ZAF0_HUMAN]
ARP2_HUMAN	Actin-related protein 2 GN=ACTR2 PE=1 SV=1 - [ARP2_HUMAN]
ARPC2_HUMAN	Actin-related protein 2/3 complex subunit 2 GN=ARPC2 PE=1 SV=1 - [ARPC2_HUMAN]
PLEC_HUMAN	Plectin GN=PLEC PE=1 SV=3 - [PLEC_HUMAN]
SDPR_HUMAN	Serum deprivation-response protein GN=SDPR PE=1 SV=3 - [SDPR_HUMAN]
PCBP1_HUMAN	Poly(rC)-binding protein 1 GN=PCBP1 PE=1 SV=2 - [PCBP1_HUMAN]
C9J0I9_HUMAN	Nuclear-interacting partner of ALK GN=ZC3HC1 PE=4 SV=1 - [C9J0I9_HUMAN]
1433E_HUMAN	14-3-3 protein epsilon GN=YWHAE PE=1 SV=1 - [1433E_HUMAN]
EF1A1_HUMAN	Elongation factor 1-alpha 1 GN=EEF1A1 PE=1 SV=1 - [EF1A1_HUMAN]
PRP6_HUMAN	Pre-mRNA-processing factor 6 GN=PRPF6 PE=1 SV=1 - [PRP6_HUMAN]
B3GQE6_HUMAN	DEAD box polypeptide 27 GN=DDX27 PE=2 SV=1 - [B3GQE6_HUMAN]
C9J9K3_HUMAN	40S ribosomal protein SA (Fragment) GN=RPSA PE=3 SV=1 - [C9J9K3_HUMAN]
VIME_HUMAN	Vimentin GN=VIM PE=1 SV=4 - [VIME_HUMAN]
B5BU24_HUMAN	14-3-3 protein beta/alpha GN=YWHAB PE=2 SV=1 - [B5BU24_HUMAN]
B7Z1R5_HUMAN	V-type proton ATPase catalytic subunit A GN=ATP6V1A PE=2 SV=1 - [B7Z1R5_HUMAN]
H3BM89_HUMAN	60S ribosomal protein L4 GN=RPL4 PE=4 SV=1 - [H3BM89_HUMAN]
SMOC1_HUMAN	SPARC-related modular calcium-binding protein 1 GN=SMOC1 PE=1 SV=1 - [SMOC1_HUMAN]
E9PF58_HUMAN	Actin-related protein 2/3 complex subunit 1A GN=ARPC1A PE=4 SV=1 - [E9PF58_HUMAN]
RPF1_HUMAN	Ribosome production factor 1 GN=RPF1 PE=1 SV=2 - [RPF1_HUMAN]
B7Z9C0_HUMAN	cDNA FLJ52405, highly similar to Myosin Ic PE=2 SV=1 - [B7Z9C0_HUMAN]
F8VWC5_HUMAN	60S ribosomal protein L18 GN=RPL18 PE=3 SV=1 - [F8VWC5_HUMAN]
I3L1L3_HUMAN	Myb-binding protein 1A (Fragment) GN=MYBBP1A PE=4 SV=1 - [I3L1L3_HUMAN]
B3KS36_HUMAN	cDNA FLJ35376 fis, clone SKMUS2004044, highly similar to Homo sapiens ribosomal protein L3 (RPL3), transcript variant 2, mRNA PE=2 SV=1 - [B3KS36_HUMAN]
B4DT35_HUMAN	Nucleoporin p54 GN=NUP54 PE=2 SV=1 - [B4DT35_HUMAN]
FLOT1_HUMAN	Flotillin-1 GN=FLOT1 PE=1 SV=3 - [FLOT1_HUMAN]

D3DN97_HUMAN	Myosin, light polypeptide kinase, isoform CRA_d GN=MYLK PE=4 SV=1 - [D3DN97_HUMAN]
VATB2_HUMAN	V-type proton ATPase subunit B, brain isoform GN=ATP6V1B2 PE=1 SV=3 - [VATB2_HUMAN]
B3KN82_HUMAN	cDNA FLJ13913 fis, clone Y79AA1000231, highly similar to Nucleolar protein NOP5 PE=2 SV=1 - [B3KN82_HUMAN]
B7Z5H9_HUMAN	cDNA FLJ53291, highly similar to Beta-galactosidase-related protein PE=2 SV=1 - [B7Z5H9_HUMAN]
B4E0K0_HUMAN	cDNA FLJ52820, highly similar to Nucleoporin p58/p45 PE=2 SV=1 - [B4E0K0_HUMAN]
MRT4_HUMAN	mRNA turnover protein 4 homolog GN=MRTO4 PE=1 SV=2 - [MRT4_HUMAN]
B2RAX6_HUMAN	cDNA, FLJ95176, Homo sapiens CGI-48 protein (CGI-48), mRNA PE=2 SV=1 - [B2RAX6_HUMAN]
H12_HUMAN	Histone H1.2 GN=HIST1H1C PE=1 SV=2 - [H12_HUMAN]
CAV1_HUMAN	Caveolin-1 GN=CAV1 PE=1 SV=4 - [CAV1_HUMAN]
E9PMV1_HUMAN	Plectin (Fragment) GN=PLEC PE=4 SV=1 - [E9PMV1_HUMAN]
C9J592_HUMAN	Ras-related protein Rab-7a (Fragment) GN=RAB7A PE=3 SV=1 - [C9J592_HUMAN]
A4FU77_HUMAN	SNRNP200 protein (Fragment) GN=SNRNP200 PE=2 SV=1 - [A4FU77_HUMAN]
WDR12_HUMAN	Ribosome biogenesis protein WDR12 GN=WDR12 PE=1 SV=2 - [WDR12_HUMAN]
H0Y6E7_HUMAN	RNA-binding motif protein, X chromosome, N-terminally processed (Fragment) GN=RBMX PE=4 SV=2 - [H0Y6E7_HUMAN]
DDX18_HUMAN	ATP-dependent RNA helicase DDX18 GN=DDX18 PE=1 SV=2 - [DDX18_HUMAN]
IF6_HUMAN	Eukaryotic translation initiation factor 6 GN=EIF6 PE=1 SV=1 - [IF6_HUMAN]
Q9H9J5_HUMAN	cDNA FLJ12694 fis, clone NT2RP1000358, highly similar to Homo sapiens mRNA; cDNA DKFZp564C186 (from clone DKFZp564C186) PE=2 SV=1 - [Q9H9J5_HUMAN]
B4DKM5_HUMAN	Voltage-dependent anion-selective channel protein 2 GN=VDAC2 PE=2 SV=1 - [B4DKM5_HUMAN]
K7EMV3_HUMAN	Histone H3.3 GN=H3F3B PE=4 SV=1 - [K7EMV3_HUMAN]
HNRPM_HUMAN	Heterogeneous nuclear ribonucleoprotein M GN=HNRNPM PE=1 SV=3 - [HNRPM_HUMAN]
B4DHC4_HUMAN	cDNA FLJ51843, highly similar to 14-3-3 protein gamma PE=2 SV=1 - [B4DHC4_HUMAN]
RBM8A_HUMAN	RNA-binding protein 8A GN=RBM8A PE=1 SV=1 - [RBM8A_HUMAN]
Q5JR95_HUMAN	40S ribosomal protein S8 GN=RPS8 PE=4 SV=1 - [Q5JR95_HUMAN]
D9YZV7_HUMAN	Tropomyosin 1 (Alpha) isoform 6 GN=TPM1 PE=3 SV=1 - [D9YZV7_HUMAN]
B4E3M6_HUMAN	cDNA FLJ55446, highly similar to Superkiller viralicidic activity 2-like 2 (EC 3.6.1.-) PE=2 SV=1 - [B4E3M6_HUMAN]
B7Z5I6_HUMAN	cDNA FLJ56516, highly similar to Nuclear pore complex protein Nup88 PE=2 SV=1 - [B7Z5I6_HUMAN]
B1ARP8_HUMAN	Mago-nashi homolog, proliferation-associated (Drosophila) GN=MAGOH PE=4 SV=1 - [B1ARP8_HUMAN]
A8K330_HUMAN	cDNA FLJ77055, highly similar to Homo sapiens WD repeat domain 75 (WDR75), mRNA (Fragment) PE=2 SV=1 - [A8K330_HUMAN]
VATG1_HUMAN	V-type proton ATPase subunit G 1 GN=ATP6V1G1 PE=1 SV=3 - [VATG1_HUMAN]
B5BUB1_HUMAN	RuvB-like 1 (Fragment) GN=RUVBL1 PE=2 SV=1 - [B5BUB1_HUMAN]
A8K897_HUMAN	cDNA FLJ78686, highly similar to Homo sapiens nucleoporin 93kDa (NUP93), mRNA PE=2 SV=1 - [A8K897_HUMAN]
NH2L1_HUMAN	NHP2-like protein 1 GN=NHP2L1 PE=1 SV=3 - [NH2L1_HUMAN]

Legend to Supplemental Note

Supplemental note: Matlab code for STED image analysis: The code for Matlab that we used to analyse the STED data are included as a supplemental file. A short description of how to implement it follows.

Supplemental note:

Instructions for use of Matlab code from McCaughey et al.

Organisation and installation

The analysis code allows the user to perform automatic analysis over multiple datasets. Images should be in TIFF format and contain a channel identifier within the name (C=0 for channel 0 and C=1 for channel 1). Each experiment can be placed in a separate folder, with multiple images allowed within each folder. The code makes use of an adaptive thresholding routine created by Guangeli Xiong which is available through the Matlab Central file exchange (http://uk.mathworks.com/matlabcentral/fileexchange/8647-local-adaptive-thresholding/content/adaptivethreshold/adaptivethreshold.m) users should download this file, unzip it and place it somewhere on the Matlab path prior to running the analysis code.

Users should navigate (1) to show the list of folders to be analysed in the "current folder" window of Matlab (2) as shown below:

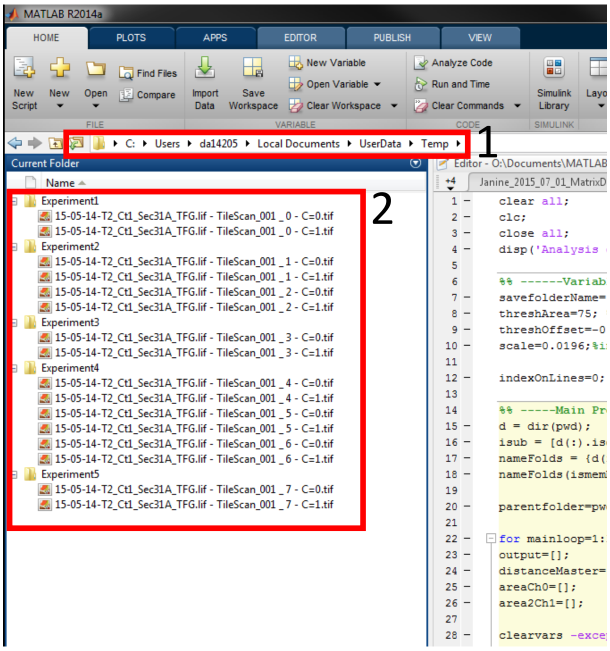

Running of code

At the top of the .m file is a section called "Variables to edit".

Editor - O:\Documents\MATLAB\Janine\Analysis_of_STED_data_2015_10_29.m

Janine_2015_07_01_MatrixData2_withAreas_imagesAllLines.m | Debbie_DEV_2015_04_14_oneExcel.m | Analysis_of_STED_da

```
clear all;
clc;
close all;
disp('Analysis of STED data.m...');

%% ------Variables to Edit--- %%
savefolderName='Analysis_New4';
threshArea=75; %kernel size for adaptive threshold
threshOffset=-0.1; %more negative = higher threshold
scale=0.0196;%in microns

indexOnLines=0; %Do you want index numbers on the 'line' images: 1 = Yes, 0=No
```

Here the user may adjust thresholding parameters used in the segmentation as well as the scale (pixel size) of the images used. Note the programme assumes the same pixel size across all images. If images with different pixel sizes were acquired, these would need to be analysed independently. Below is a summary of the adjustable variables and their meaning.

Variable name	description
savefolderName	Name of results folder (one created per experiment folder)
threshArea	Kernel size for adaptive threshold
threshOffset	Adjustment to automatic thresholding, more negative= higher threshold
scale	Pixel size in microns
indexOnLines	Display index numbers on images. 1=Yes, 0=No.

More variable can be found throughout the main programme (size of Weiner kernel etc.) which the user is free to edit, but these have been tuned for this particular dataset and were therefore kept constant.

Once the required variable have been inputted, the user simply runs the entire code by clicking “Run” whilst on the Editor tab.

<u>Data Output</u>

For each field of view the programme create three images which are saved with the field of view name plus one of the following:

1. “filteredMerge2” – shows the smoothed data, overlaid with the indexes of the detected objects and the lines joining any neighbouring objects.
2. “layered” – shows each layer of the above image on a separate page of a multipage TIFF file.
3. “originalMerge2” – shows the original (unsmoothed) data overlaid with the indexes o of the detected objects and the lines joining any neighbouring objects.

The programme also creates an excel spreadsheet for each field of view analysed containing tabs for the measure area of the objects and the distance between objects, all values are given in microns. The name given to the excel file will be the same as the folder containing the images

Legends to Supplemental Movies

Supplemental Movie 1: ER-to-Golgi transport of mannosidase II-GFP in control (GL2 siRNA-transfected) cells.

Imaging of biotin-dependent export of mannII-GFP from the ER in cells expressing GRASP65-mCherry to monitor delivery of cargo from the ER to the Golgi in control (GL2 siRNA-transfected) cells.

Supplemental Movie 2: ER-to-Golgi transport of mannosidase II-GFP in TFG-depleted cells.

Imaging of biotin-dependent export of mannII-GFP from the ER in cells expressing GRASP65-mCherry to monitor delivery of cargo from the ER to the Golgi in TFG-depleted cells.
